# Mechanistic Studies on the 1,2-Spin-Center Shift in Carbohydrate Systems with a Fluorenylcyclopropyl Radical Clock

**DOI:** 10.1021/acs.joc.3c01069

**Published:** 2023-08-22

**Authors:** Collin H. Witt, K. A. Woerpel

**Affiliations:** Department of Chemistry, New York University, 100 Washington Square East, New York, New York 10003, United States

## Abstract

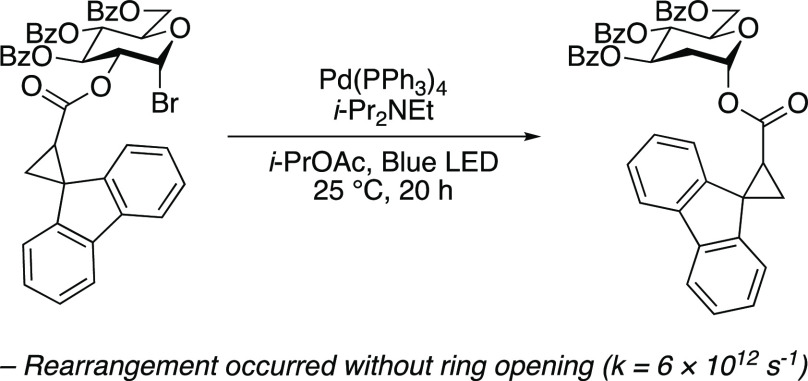

The mechanism of the 1,2-spin-center shift in carbohydrate systems was studied with a fluorenylcyclopropyl radical clock. The 1,2-rearrangement of the acyl fluorenylcyclopropane group without opening of the cyclopropane ring provides the strongest evidence that the 1,2-spin-center shift in carbohydrate systems occurs through a concerted transition state without the intermediacy of a 1,3-dioxolanyl radical.

The manipulation of carbohydrate scaffolds is important for the synthesis of bioactive compounds.^1−3^ The 1,2-spin-center shift (1,2-SCS), also termed the Surzur–Tanner rearrangement,^4−6^ has emerged as a reliable method for the functionalization of carbohydrates.^7,8^ A general scheme for the 1,2-SCS is depicted in Figure 1a: carbohydrate **A** undergoes C–X homolysis or group transfer to generate anomeric radical **B**. The anomeric radical is then translocated to the C2 position by the migration of the adjacent acyloxy group to the C1 position, which generates the more stabilized C2-alkyl radical **C**. Classically, a C2 radical would be reduced with an H atom donor to form deoxy sugars **D** (R^1^ = H).^9,10^ Recent work has shown that the C2 alkyl radical can combine with excited-state palladium^11−13^ or nickel^14^ complexes to engage in a variety of transition-metal-catalyzed transformations to form C2-functionalized products with the general structure of **D**, where R^1^ = H, D, I, CH_2_COR, or CH=CHR.

**Figure 1:**
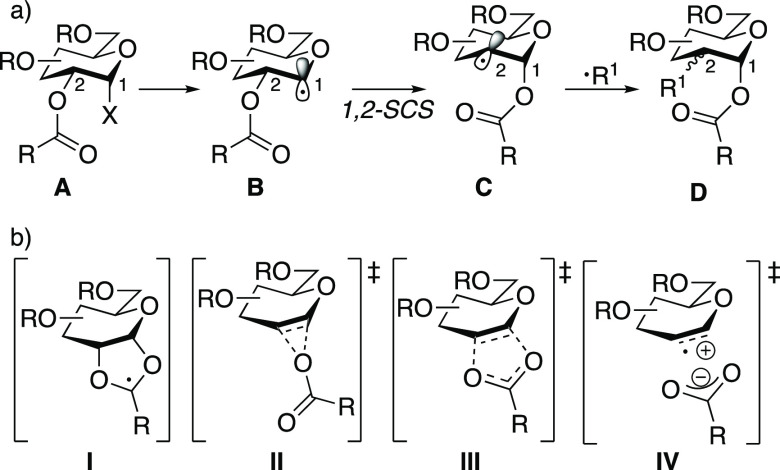
(a) Mechanism of 1,2-spin-center shift in carbohydrates. (b) Four possible modes of conversion from **B** to **C**.

The mechanism of the 1,2-SCS (i.e., the transformation **B** → **C**, Figure 1a) has been investigated extensively since its initial discovery.^6,15^ The four possible mechanisms that have received the most attention are depicted in Figure 1b.^16^ The intermediacy of dioxolanyl radical **I** has been deemed unlikely based on radical clock experiments with acyl cyclopropanes. For example, under excited-state palladium conditions, glycosyl bromide **1** underwent a 1,2-SCS without opening of the cyclopropyl ring to generate **2** (eq 1).^11^ ESR spectroscopy^17,18^ and experimental studies^19^ on dioxolanyl radical intermediates **4** and **7** showed that cyclopropyl dioxolanyl radicals prefer to open to the cyclopropane ring over the dioxolane (eqs 2 and 3). Taken together, these experiments suggest that dioxolanyl radical intermediates such as **I** are not competent intermediates for 1,2-SCS in carbohydrates. These experimental findings have also been supported by computational studies.^20^
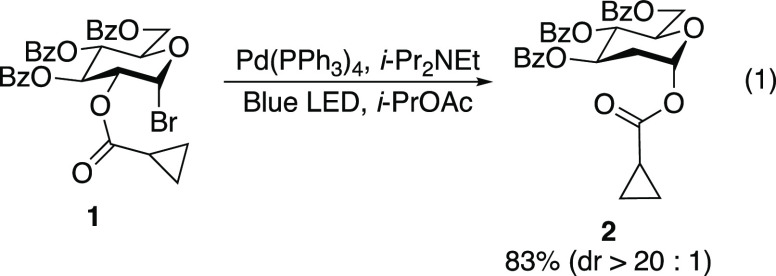
1
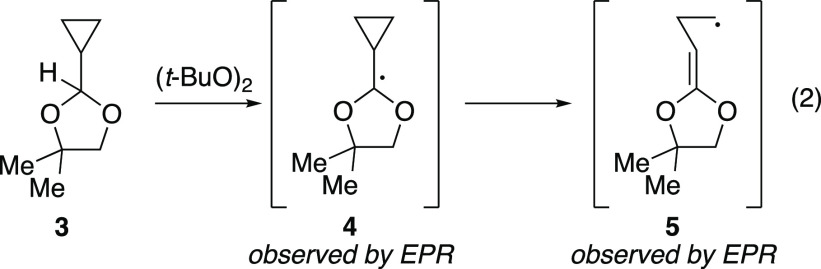
2
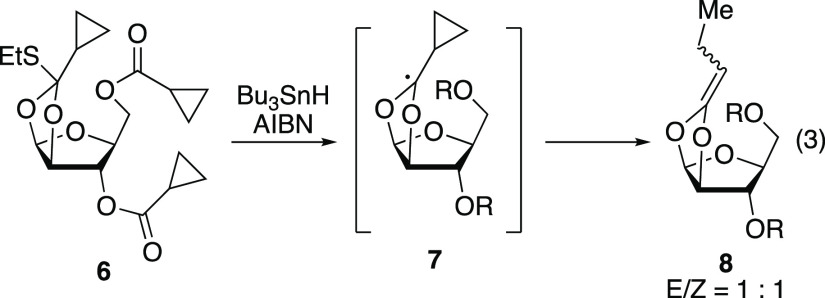
3

To account for the unlikelihood of a pathway that proceeds through dioxolanyl intermediate **I**, three concerted transition states have been proposed. A three-centered transition state such as **II** was proposed by Kočovsky,^21^ but this mechanism was subsequently disproven (for nonconformationally restricted ester substrates)^22^ by ^18^O labeling studies showing that labeled glycosyl bromide **9** underwent clean transposition to afford deoxy sugar **10a** (eq 4).^17^ A three-membered transition state like **II** would occur with retention of the ^18^O label at the carbonyl oxygen atom, which would have led to the exclusive formation of **10b**. The results from this ^18^O labeling study provided evidence for a five-electron, five-membered concerted transition state such as **III**.^17,23^
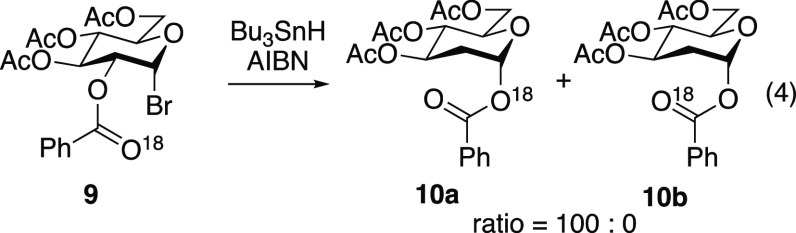
4

Doubt has been cast on transition state **III** by separate isotopic labeling studies with ^17^O in the tetrahydropyranyl systems. When ^17^O-labeled tetrahydropyran **11** was treated with tri-*n*-butyltin hydride, only 33% transposition of the carbonyl oxygen atom of the acyl group to the C2 carbon atom was observed (eq 5).^24^ This labeling experiment contradicts the possibility of a five-centered concerted transition state such as **III**.^24^ This discrepancy was reasoned by suggesting that the mechanism of 1,2-SCS is substrate-dependent and that tetrahydropyranyl radicals undergo 1,2-SCS, at least in part, through the three-membered transition state **II** or a charge-separated transition state such as **IV** where scrambling of the label would be possible. The current explanation for the observed scrambling of the isotopic label is that the lack of substituents on tetrahydropyran **11** causes this substrate to rearrange via a looser charge-separated transition state (such as **IV**) than the transition state for the analogous glucosyl substrate **9**, which has its charge-separated pathway inductively destabilized by adjacent acyloxy substituents.^25^
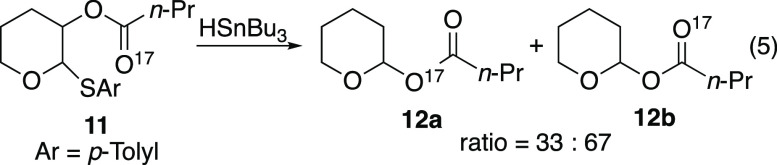
5

We sought to address the conflicting mechanistic picture for the 1,2-SCS in carbohydrate systems by formally excluding the possibility of **I** as a reactive intermediate using a fluorenylcyclopropyl radical clock. While radical clock experiments with unsubstituted acyl cyclopropyl substrates have been previously conducted for 1,2-SCS reactions of carbohydrates (for example, eqs 2 and 3),^11,18,19^ these experiments only provide accurate information if the radical intermediate in question exists for longer than the rate of ring opening of an unsubstituted cyclopropane, which is roughly 1 × 10^8^ s^–1^ at 25 °C.^26,27^ Considering that dioxolanyl intermediate **I** was not observed by ESR spectroscopy, it is unsurprising that intermediate **I** could not be trapped (if it exists) by cyclopropane ring-opening of **B** to **C** because EPR is on the nanosecond time scale.^28^ With a rate of 6 × 10^12^ s^–1^ at 25 °C,^29,30^ the cyclopropyl ring opening of a fluorenylcyclopropyl radical clock occurs sufficiently fast to trap a fleeting dioxolanyl intermediate **I** should it last for a time shorter than the EPR time scale. Moreover, it is unknown if transition metals influence the mechanism of the 1,2-SCS in carbohydrates to include dioxolanyl radical intermediates. 1,2-Spin-center shifts in acyclic systems with copper^31^ and palladium^32^ reagents have been reported to proceed through mechanisms that deviate from the five-centered concerted transition state. In addition, a reported example of a 1,2-SCS in a carbohydrate (albeit from a non-anomeric radical intermediate) that proceeded through a 1,3-dioxolanyl radical intermediate suggests that the formation of such an intermediate in this context may be feasible.^33^

Carbohydrate substrates bearing acyl fluorenylcyclopropyl radical clocks at C2 were prepared. Glycosyl bromide **13**(34,35) and fluorenylcyclopropyl acid **14**(36) were synthesized using known synthetic sequences. The DCC-mediated coupling of glycosyl bromide **13** and acid **14** proceeded sluggishly to afford acyl radical clock compound **15** as a nearly equal mixture of diastereomers (eq 6). Likewise, tetrahydropyranol **16** was coupled with acid **14** to afford the unsubstituted carbohydrate derivative **17** as an inconsequential mixture of four diastereomers (eq 7).
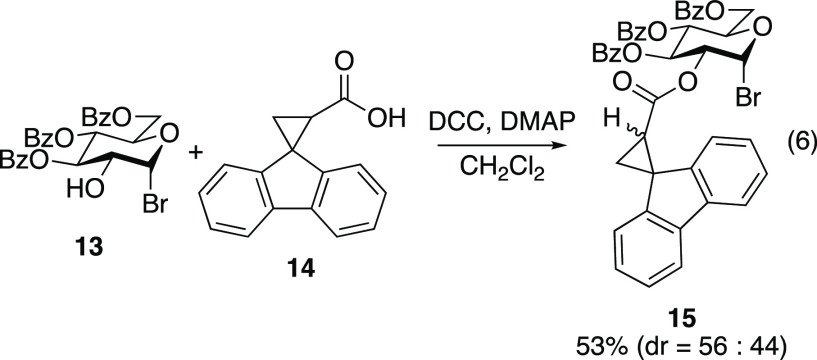
6
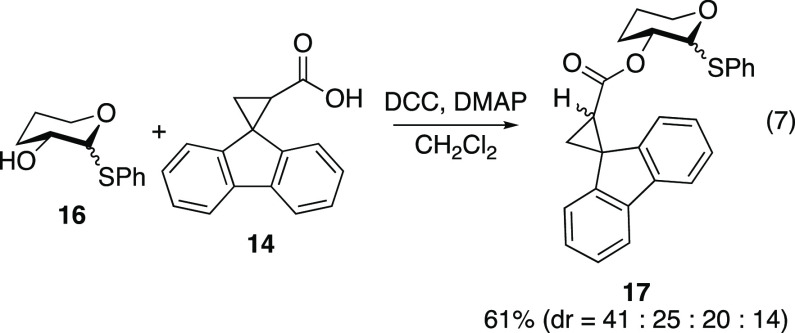
7

Both fully substituted carbohydrate **15** and unsubstituted tetrahydropyran **17** underwent 1,2-SCS without opening of the fluorenylcyclopropyl radical clock. When carbohydrate **15** was subjected to Ngai’s excited-state palladium-catalyzed C2 reduction conditions,^11^ rearrangement product **18** was observed in the crude reaction mixture (eq 8). To verify that the 1,2-SCS occurred without opening of the cyclopropane ring, rearrangement product **18** was subjected to methanolysis to afford fluorenylcyclopropyl methyl ester **19** and various benzoylated C2-deoxy sugars (eq 9). Under modified conditions to generate the anomeric radical,^37,38^ tetrahydropyran **17** was found to rearrange to product **20** without cyclopropane ring opening (eq 10). These results suggest that 1,2-SCS of both fully elaborated sugars and simple pyrans does not rearrange through dioxolanyl intermediates such as **I**.
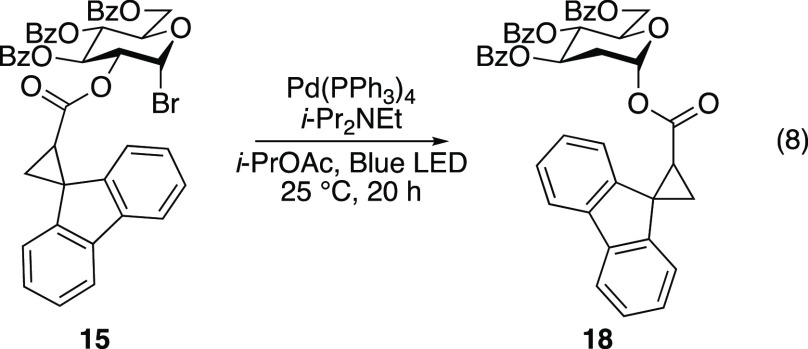
8
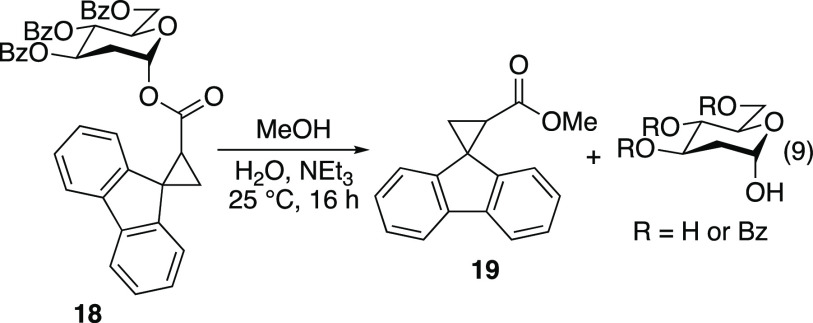
9
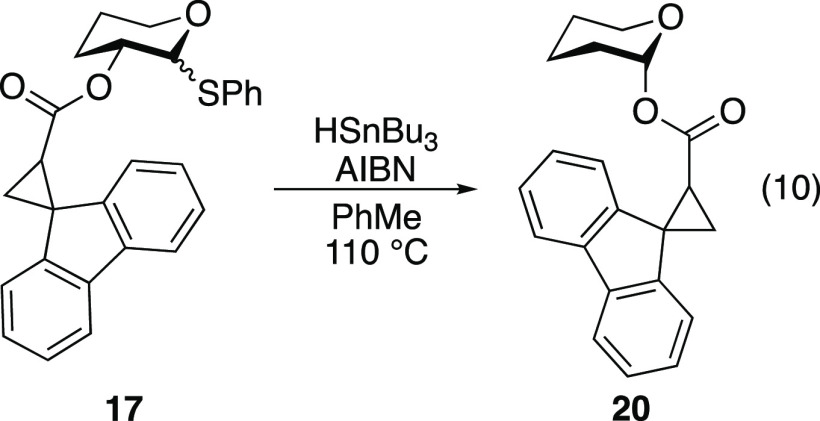
10

In conclusion, the rearrangements of radical clocks **15** and **17** via 1,2-SCS mechanisms without opening of their fluorenylcyclopropyl radical provide strong evidence that the 1,2-SCS occurs without the intermediacy of a dioxolanyl radical intermediate. While these radical clock experiments all but exclude the possibility of dioxolanyl intermediate **I**’s existence, it should be noted that concerted processes **II**, **III**, and **IV**, which have been analyzed previously,^25^ cannot be discriminated by the results of this radical clock study. We hope that by disproving the existence of **I** that future synthetic methods that leverage the 1,2-SCS need not consider the possibility of dioxolanyl radical **I** as a reactive intermediate.^11^

## Experimental Section

### General Information

^1^H NMR and ^13^C{^1^H} NMR were measured at ambient temperature using Bruker AV-400 (400 and 100 MHz, respectively) and Bruker AVIII-400 (400 and 100 MHz, respectively) spectrometers unless otherwise noted. All spectroscopic data were reported as follows: chemical shifts in parts per million on the δ scale referenced from residual solvent peaks (^1^H NMR: CDCl_3_ δ 7.26 ppm; ^13^C{^1^H} NMR: CDCl_3_ δ 77.16 ppm), multiplicity (s = singlet, br = broad, d = doublet, t = triplet, q = quartet, m = multiplet, AB = AB system, and ABX = ABX system), coupling constants (Hz), and integration. Multiplicity of carbon peaks was determined using HSQC experiments. Ratios of products were determined by ^13^C{^1^H} NMR experiments^39^ and confirmed by ^1^H NMR experiments. Infrared (IR) spectra were acquired by a Nicolet 6700 FT-IR spectrometer through attenuated total reflectance (ATR). High-resolution mass spectra (HRMS) were recorded using an Agilent 6224 accurate-mass time-of-flight spectrometer with atmospheric pressure chemical ionization (APCI) or electrospray ionization (ESI) ionization sources. Analytical thin layer chromatography was performed on silica gel 60 Å F_254_ plates. All reactions were performed under a nitrogen atmosphere in glassware that had been flame-dried under a vacuum unless otherwise noted. Nondeuterated solvents were dried and purified through alumina prior to use. Aqueous solutions were prepared from nanopore water with a resistivity over 18 MΩ-cm. Light-promoted reactions were performed in 1 dram borosilicate glass vials without the use of any additional filters unless otherwise noted. All reagents were commercially available, unless otherwise stated.

#### Synthesis of Fluorenylcyclopropyl Carboxylic Acid **14**

Methyl ester **19** had previously been prepared for other radical clock studies.^36^ Methyl ester **19** (2.07 g, 8.28 mmol) was dissolved in THF (20 mL) and 2 N LiOH (20 mL). The reaction mixture was stirred vigorously at 25 °C for 16 h. THF was removed *in vacuo*, and the aqueous residue was acidified with 1 N HCl until a pH of 1 was observed. The acidified aqueous layer was extracted with EtOAc (3 × 40 mL). The combined organic layers were dried over MgSO_4_, filtered, and concentrated *in vacuo* to afford carboxylic acid **14** as an orange solid (1.70 g, 87%): mp = 223–227 °C; ^1^H NMR (400 MHz, CDCl_3_) δ 10.29 (br s, 1H), 7.81 (dd, *J* = 7.5, 3.5 Hz, 2H), 7.56 (d, *J* = 7.8 Hz, 1H), 7.39 (tdd, *J* = 7.5, 3.2, 0.9 Hz, 2H), 7.32 (td, *J* = 7.5, 0.9 Hz, 1H), 7.26 (td, *J* = 7.6, 0.3 Hz, 1H), 7.05 (d, *J* = 7.5 Hz, 1H), 2.75 (t, *J* = 7.9 Hz, 1H), 2.43 (dd, *J* = 7.5, 5.4 Hz, 1H), 2.19 (dd, *J* = 8.3, 5.3 Hz, 1H); ^13^C{^1^H} NMR (100 MHz, CDCl_3_) δ 173.7 (C), 146.1 (C), 142.0 (C), 141.1 (C), 139.9 (C), 127.25 (CH), 127.21 (CH), 126.98 (CH), 122.9 (CH), 119.9 (CH), 118.7 (CH), 37.8 (C), 32.2 (CH), 21.0 (CH_2_); IR (ATR) 2573, 1693, 1435, 1235, 1090, 937, 732 cm^–1^; HRMS (ESI) *m*/*z* calcd for C_16_H_11_O (M+H)^+^[-H_2_O] 219.0804, found 219.0808.

#### Synthesis of Radical Clock **15**

To a stirred solution of glycosyl bromide **13** (1.78 g, 3.20 mmol) and carboxylic acid **14** (0.755 g, 3.20 mmol) in CH_2_Cl_2_ (32 mL) at 0 °C were added DMAP (0.0391 g, 0.320 mmol) and *N,N*′-dicyclohexylcarbodiimide (0.792 g, 3.84 mmol). The reaction mixture was stirred at 0 °C and was allowed to warm to 25 °C with stirring for 24 h. The reaction mixture was filtered, and the filtrate was concentrated *in vacuo*. The residue was redissolved in EtOAc (60 mL). The organic layer was washed with brine (1 × 30 mL), 1 N HCl (1 × 30 mL), brine (1 × 30 mL), saturated aqueous NaHCO_3_ (1 × 30 mL), and brine (1 × 30 mL). The organic layer was dried over MgSO_4_, filtered, and concentrated *in vacuo*. Purification by flash chromatography (10:90 to 20:80 EtOAc/hexanes) afforded inseparable 54:46 mixture radical clocks **15a** and **15b** as a white solid (1.31 g, 53%). Characterization was performed on a 54:46 mixture of diastereomers (stereochemistry not assigned): mp = 82–112 °C; Major diastereomer: ^1^H NMR (400 MHz, CDCl_3_, distinctive peaks) 6.27 (d, *J* = 3.9 Hz, 1H), 5.96 (t, *J* = 9.8 Hz, 2H), 5.68 (t, *J* = 9.8 Hz, 1H), 5.17 (dd, *J* = 10.0, 4.0 Hz, 1H), 2.70 (t, *J* = 7.8 Hz, 1H), 2.08 (dd, *J* = 8.2, 5.5 Hz, 1H); ^13^C{^1^H} NMR (100 MHz, CDCl_3_, distinctive peaks) δ 168.5 (C), 165.96 (C), 165.3 (C), 165.1 (C), 145.7 (C), 141.7 (C), 141.1 (C), 139.9 (C), 133.6 (CH), 133.4 (CH), 133.23 (CH), 129.3 (CH), 122.2 (CH), 120.1 (CH), 120.0 (CH), 118.8 (CH), 86.1 (CH), 72.4 (CH), 71.0 (CH), 70.4 (CH), 68.2 (CH), 37.7 (C), 32.1 (CH), 20.2 (CH_2_). Minor diastereomer: ^1^H NMR (400 MHz, CDCl_3_, distinctive peaks): δ 6.83 (d, *J* = 3.9 Hz, 1H), 5.95 (t, *J* = 9.8 Hz, 2H), 5.62 (t, *J* = 9.9 Hz, 1H), 5.02 (dd, *J* = 10.1, 4.0 Hz, 1H), 2.77 (t, *J* = 7.9 Hz, 1H), 2.15 (dd, *J* = 8.3, 5.4 Hz, 1H); ^13^C{^1^H} NMR (100 MHz, CDCl_3_, distinctive peaks) δ 168.8 (C), 165.95 (C), 165.2 (C), 165.0 (C), 145.5 (C), 141.4 (C), 140.7 (C), 139.6 (CH), 133.5 (CH), 133.22 (CH), 132.7 (CH), 129.4 (CH), 122.4 (CH), 119.76 (CH), 119.75 (CH), 118.7 (CH), 86.9 (CH), 72.6 (CH), 71.5 (CH), 70.0 (CH), 68.0 (CH), 37.8 (C), 31.8 (CH), 21.1 (CH_2_). IR (ATR) 1724, 1450, 1260, 1091, 1067, 1024 705 cm^–1^; [α]_D_^23^+70.9 (*c* 1.00, CHCl_3_); HRMS (ESI) *m*/*z* calcd for C_43_H_33_BrNaO_9_ (M+Na)^+^ 797.1189, found 797.1229.

#### Synthesis of Radical Clock **17**

To a stirred solution of thiols *cis*-**16** and *trans*-**16** (0.319 g, 1.52 mmol) and carboxylic acid **14** (0.358 g, 1.52 mmol) in CH_2_Cl_2_ (15 mL) at 0 °C were added DMAP (0.0185 g, 0.152 mmol) and *N,N*′-dicyclohexylcarbodiimide (0.376 g, 1.82 mmol). The reaction mixture was stirred at 0 °C and was allowed to warm to 25 °C while stirring for 24 h. The reaction mixture was filtered, and the filtrate was concentrated *in vacuo*. The residue was redissolved in EtOAc (30 mL). The organic layer was washed with brine (1 × 15 mL), 1 N HCl (1 × 15 mL), brine (1 × 15 mL), saturated aqueous NaHCO_3_ (1 × 15 mL), and brine (1 × 15 mL). The organic layer was dried over MgSO_4_, filtered, and concentrated *in vacuo*. Purification by flash chromatography (5:95 to 10:90 EtOAc/hexanes) afforded an inseparable 41:25:20:14 mixture of radical clocks *cis*-**17a**, *cis*-**17b**, *trans*-**17a**, and *trans*-**17b** as an off-white solid (0.397 g, 61%). Characterization was performed on a 41:25:20:14 mixture of diastereomers (stereochemistry not assigned, labeled as diastereomers 1–4 in decreasing order of abundance): mp = 48–65 °C; ^1^H NMR (400 MHz, CDCl_3_, distinctive peaks) δ 5.40 (d, *J* = 5.40 Hz, 1H, diastereomer 4), 5.22–5.20 (m, 2H, overlap of diastereomers 2 and 3), 5.10–5.04 (m, 2H, overlap of diastereomers 3 and 4), 4.96–4.91 (m, 2H, overlap of diastereomers 1 and 2), 4.86 (d, *J* = 3.8 Hz, 1H, diastereomer 1), 4.02 (ddd, *J* = 11.6, 8.0, 3.7 Hz, 1H, diastereomer 4), 2.61 (dd, *J* = 7.3, 5.6 Hz, 1H, diastereomer 3); ^13^C{^1^H} NMR (126 MHz, CDCl_3_, distinctive peaks) δ 168.0 (C), 168.8 (C), 142.3 (C), 140.0 (C), 134.1 (C), 131.7 (CH), 131.6 (CH), 131.44 (CH), 131.40 (CH), 128.9 (CH), 128.8 (CH), 128.7 (CH), 127.3 (CH), 127.1 (CH), 126.9 (CH), 123.2 (CH), 122.8 (CH), 120.1 (CH), 119.90 (CH), 119.86 (CH), 118.8 (CH), 118.7 (CH), 87.3 (CH), 86.9 (CH), 86.4 (CH), 70.31 (CH), 70.25 (CH), 63.2 (CH_2_), 62.7 (CH_2_), 62.6 (CH_2_), 37.5 (C), 32.7 (CH), 25.9 (CH_2_), 25.8 (CH_2_), 25.6 (CH_2_), 21.6 (CH_2_), 21.3 (CH_2_), 21.0 (CH_2_), 20.9 (CH_2_), 20.7 (CH_2_); IR (ATR) 2929, 1725, 1447, 1393, 1169, 935, 748 cm^–1^; HRMS (ESI) *m*/*z* calcd for C_27_H_24_NaO_3_S (M+Na)^+^ 451.1338, found 451.1346.

#### Synthesis of Authentic Sample of 1,2-Rearrangement Product **18**

To a stirred solution of (2*R*,3*S*,4*R*,6*S*)-2-((benzoyloxy)methyl)-6-hydroxytetrahydro-2*H*-pyran-3,4-diyl dibenzoate (details of synthesis are provided in the Supporting Information; 0.200 g, 0.419 mmol) and carboxylic acid **14** (0.0989 g, 0.419 mmol) in CH_2_Cl_2_ (4 mL) at 0 °C were added DMAP (0.0051 g, 0.042 mmol) and *N,N*′-dicyclohexylcarbodiimide (0.104 g, 0.503 mmol). The reaction mixture was stirred at 0 °C and was allowed to warm to 25 °C while stirring for 24 h. The reaction mixture was filtered, and the filtrate was concentrated *in vacuo*. The residue was redissolved in EtOAc (5 mL). The organic layer was washed with brine (1 × 5 mL), 1 N HCl (1 × 5 mL), salt (1 × 5 mL), saturated aqueous NaHCO_3_ (1 × 5 mL), and salt (1 × 5 mL). The organic layer was dried over MgSO_4_, filtered, and concentrated *in vacuo*. Purification by flash chromatography (20:80 EtOAc/hexanes) afforded an inseparable 58:42 mixture of rearranged radical clocks **18a** and **18b** as a pale-yellow solid (0.149 g, 51%). Characterization was performed on a 52:42 mixture of diastereomers (stereochemistry not assigned): mp = 87–102 °C; ^1^H NMR (400 MHz, CDCl_3_, distinctive peaks) δ 6.35 (dd, *J* = 7.7, 2.6 Hz, 1H), 5.99 (dd, *J* = 9.7, 2.3 Hz, 1H), 4.43 and 4.33 (ABX, *J*_AB_ = 12.2 Hz, *J*_AX_ = 3.1 Hz, *J*_BX_ = 4.9 Hz, 2H), 4.04 and 3.98 (ABX, *J*_AB_ = 12.5 Hz, *J*_AX_ = 3.1 Hz, *J*_BX_ = 2.8 Hz, 2H), 3.65 (dt, *J* = 9.8, 2.8 Hz, 1H), 2.94 (t, *J* = 7.8 Hz, 1H), 2.78 (t, *J* = 7.9 Hz, 1H), 2.69 (ddd, *J* = 12.5, 4.8, 2.3 Hz, 1H); ^13^C{^1^H} NMR (100 MHz, CDCl_3_, distinctive peaks) δ 167.71 (C), 167.66 (C), 166.1 (C), 166.0 (C), 165.8 (C), 165.7 (C), 165.3 (C), 165.1 (C), 146.1 (C), 146.0 (C), 145.9 (C), 141.81 (C), 141.76 (C), 141.0 (C), 148.8 (C), 140.0 (C), 133.4 (CH), 133.3 (CH), 133.23 (CH), 133.21 (CH), 133.15 (CH), 133.06 (CH), 132.94 (CH), 132.91 (CH), 129.8 (CH), 129.73 (CH), 129.69 (CH), 129.63 (CH), 129.3 (CH), 129.2 (CH), 129.02 (CH), 128.95 (CH), 128.41 (CH), 128.39 (CH), 128.37 (CH), 128.34 (CH), 128.32 (CH), 128.27 (CH), 128.24 (CH), 127.5 (CH), 127.33 (CH), 127.30 (CH), 127.25 (CH), 127.19 (CH), 127.13 (CH), 127.10 (CH), 126.9 (CH), 126.8 (CH), 122.89 (CH), 122.5 (CH), 120.05 (CH), 120.00 (CH), 119.99 (CH), 119.92 (CH), 119.86 (CH), 119.0 (CH), 118.6 (CH), 91.9 (CH), 91.4 (CH), 72.9 (CH), 70.9 (CH), 69.9 (CH), 69.5 (CH), 69.1 (CH), 69.0 (CH), 63.1 (CH_2_), 62.2 (CH_2_), 37.8 (C), 37.3 (C), 34.9 (CH_2_), 34.0 (CH_2_), 32.5 (CH), 32.3 (CH), 21.0 (CH_2_), 20.4 (CH_2_); IR (ATR) 1721, 1449, 1264, 1172, 1069, 741 cm^–1^; [α]_D_^24^+55.6 (*c* 1.00, CHCl_3_); HRMS (ESI) *m*/*z* calcd for C_43_H_34_NaO_9_ (M+Na)^+^ 717.2095, found 717.2131.

#### Synthesis of Authentic Sample of 1,2-Rearrangement Product **20**

To a stirred solution of hemiacetal tetrahydro-2*H*-pyran-2-ol (details of synthesis are provided in the Supporting Information; 0.0473 g, 0.463 mmol) and carboxylic acid **14** (0.164 g, 0.695 mmol) in CH_2_Cl_2_ (5 mL) at 0 °C were added DMAP (0.0057 g, 0.046 mmol) and *N,N*′-dicyclohexylcarbodiimide (0.115 g, 0.556 mmol). The reaction mixture was stirred at 0 °C and was allowed to warm to 25 °C while stirring for 24 h. The reaction mixture was filtered, and the filtrate was concentrated *in vacuo*. The residue was redissolved in EtOAc (5 mL). The organic layer was washed with brine (1 × 5 mL), 1 N HCl (1 × 5 mL), brine (1 × 5 mL), saturated aqueous NaHCO_3_ (1 × 5 mL), and brine (1 × 5 mL). The organic layer was dried over MgSO_4_, filtered, and concentrated *in vacuo*. Purification by flash chromatography (5:95 EtOAc/hexanes) afforded an inseparable 62:38 mixture of radical clocks **20a** and **20b** as a yellow oil (0.0306 g, 21%). Characterization was performed on a 62:38 mixture of diastereomers (stereochemistry not assigned): Major diastereomer: ^1^H NMR (400 MHz, CDCl_3_, distinctive peak) δ 5.96 (t, *J* = 2.6 Hz, 1H); ^13^C{^1^H} NMR (100 MHz, CDCl_3_, distinctive peaks) δ 168.4 (C), 142.3 (C), 140.8 (C), 140.0 (C), 122.8 (CH), 119.81 (CH), 118.7 (CH), 93.2 (CH), 63.0 (CH_2_), 37.0 (C), 32.81 (CH), 28.95 (CH_2_), 24.7 (CH_2_), 20.6 (CH_2_), 18.1 (CH_2_). Minor diastereomer: ^1^H NMR (400 MHz, CDCl_3_, distinctive peak) δ 5.93 (t, *J* = 2.8 Hz, 1H); ^13^C{^1^H} NMR (100 MHz, CDCl_3_, distinctive peaks) δ 168.3 (C), 142.4 (C), 140.9 (C), 139.9 (C), 123.1 (CH), 119.83 (CH), 118.8 (CH), 93.4 (CH), 62.2 (CH_2_), 37.4 (C), 32.77 (CH), 29.04 (CH_2_), 24.8 (CH_2_), 20.0 (CH_2_), 18.3 (CH_2_). IR (ATR) 2943, 1735, 1450, 1164, 1123, 904, 865, 647 cm^–1^; HRMS (ESI) *m*/*z* calcd for C_21_H_20_NaO_3_ (M+Na)^+^ 343.1305, found 343.1312.

#### 1,2-Spin-Center Shift of Radical Clock **15**

A reported procedure^11^ was followed for the 1,2-spin-center shift of radical clock **15**. Glucosyl bromide **15** (0.152 g, 0.197 mmol), Pd(PPh_3_)_4_ (0.011 g, 0.0098 mmol), diisopropylethylamine (0.0684 mL, 0.393 mmol), and isopropyl acetate (degassed, 4 mL) were added to a 4 mL vial equipped with a stir bar in a glovebox. The vial was capped and was removed from the glovebox, where the cap was immediately wrapped in Parafilm to prevent exposure to air. The reaction mixture was irradiated with blue light using a Feit Electric 7 W blue LED lamp (SKU: PAR38/B/10KLED/BX) and a 34 W Kessil Lamp (456 nm), which were each positioned 8 cm from the reaction vessel at approximately 45° angles. The reaction mixture was stirred vigorously under blue light irradiation for 20 h. The reaction mixture was concentrated *in vacuo*. Careful purification by flash chromatography permitted the isolation of rearranged products **18a** and **18b** (0.0435 g). The spectroscopic data for rearranged products **18a** and **18b** are consistent with those of the authentic sample (*vide supra*). Analysis of the crude reaction mixture by ^1^H and ^13^C{^1^H} NMR spectroscopy revealed the presence of unreacted glucosyl bromide **15** and rearranged products **18a** and **18b** (Figures S1 and S2 in the Supporting Information). The presence of rearranged products **18a** and **18b** provides strong evidence that the reaction does not proceed via a dioxolanyl radical intermediate.

#### 1,2-Spin-Center Shift of Radical Clock **17**

A reported procedure^37^ was followed for the 1,2-spin-center shift of radical clock **17**. A solution of thiol **17** (0.0681 g, 0.159 mmol) in toluene (1 mL) was added dropwise over 15 min to a refluxing (110 °C oil bath) solution of AIBN (0.0026 g, 0.016 mmol) and HSnBu_3_ (0.206 mL, 0.763 mmol) in toluene (2.5 mL). The reaction mixture was stirred at reflux for 16 h. The reaction mixture was cooled to 25 °C and concentrated *in vacuo*. Analysis of the crude reaction mixture revealed poor conversion of starting material to product, but the presence of rearranged products **20a** and **20b** suggested that the rearrangement occurred without rearrangement of the fluorenylcyclopropyl group, which implies there is no intermediacy of a dioxolanyl radical intermediate (Figure S3 in the Supporting Information).

## Data Availability

The data underlying this study are available in the published article and its Supporting Information.

## Supporting Information Available

Full experimental details and ^1^H, ^13^C{^1^H}, and HSQC spectra for new compounds (PDF)
