# Supplementary material for: Mechanistic Studies on the 1,2-Spin-Center Shift in Carbohydrate Systems with a Fluorenylcyclopropyl Radical Clock
Source: J Org Chem. 2023 Aug 22;88(17):12802–7. doi: 10.1021/acs.joc.3c01069 (PMC10476153; doi:10.1021/acs.joc.3c01069)
Supplement: Supplementary file 1 — jo3c01069_si_001.pdf [file jo3c01069_si_001.pdf]

Supporting Information

Mechanistic Studies on the 1,2-Spin-Center Shift in Carbohydrate Systems with a Fluorenylcyclopropyl Radical Clock

Collin H. Witt and K. A. Woerpel*

Department of Chemistry, New York University
100 Washington Square East, New York, NY 10003, United States

*Email: kw63@nyu.edu

Table of Contents

Synthesis of Glycosyl Bromide 13 2
Synthesis of Tetrahydropyranyl Alcohol 16 4
Syntheses of Precursors of Authentic Samples of Rearrangement Products 6
1,2-Spin-Center Shift Reactions of Radical Clocks 8
Methanolysis of 1,2-Spin-Center Shift Rearrangement Product 18 11
References 12
^{1}H and ^{13}C NMR Spectra for New Compounds 13

Synthesis of Glycosyl Bromide 13

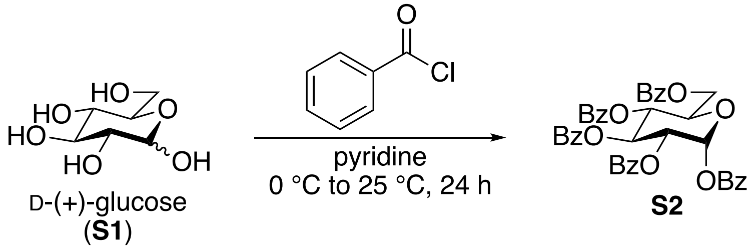

1,2,3,4,6-Penta-*O*-benzoyl-α-D-glucopyranose (S2). A reported procedure[2] was adapted for the synthesis of benzoylated glucose **S2**. To a stirred solution of D-(+)-glucose **S1** (10.1 g, 56.5 mmol) in pyridine (120 mL) at 0 °C was added benzoyl chloride (42.0 mL, 363 mmol) dropwise by syringe pump over a period of 30 min. The reaction mixture was allowed to warm to 25 °C and was stirred for 24 h. MeOH (60 mL) was added and the reaction mixture was stirred for an additional 15 min. The mixture was diluted with CH_2Cl_2 (200 mL). The organic layer was washed with 1 N aqueous HCl (1 × 200 mL), H_2O (1 × 200 mL), and brine (1 × 200 mL). The organic layer was dried over $MgSO_4$, filtered, and concentrated *in vacuo*. Recrystallization from a mixture of ethyl acetate/hexanes afforded 17.65 g of benzoylated glucose **S2** as a white solid. Recrystallization of the mother liquor afforded an additional 6.17 g crop of **S2** as a white solid (23.8 g total, 61%). The spectroscopic data are consistent with previously reported data for benzoylated glucose **S2**:[2]

^{1}H NMR (400 MHz, $CDCl_3$) δ 8.18–8.16 (m, 2H), 8.04–8.02 (m, 2H), 7.96–7.94 (m, 2H), 7.89 (d, *J* = 8.1 Hz, 4H), 7.69–7.65 (m, 1H), 7.57–7.29 (m, 9H), 6.86 (d, *J* = 3.7 Hz, 1H), 6.32 (t, *J* = 10.0 Hz, 1H), 5.86 (t, *J* = 9.8 Hz, 1H), 5.69 (dd, *J* = 10.2, 3.7 Hz, 1H), 4.65–4.60 (m, 2H), 4.51–4.47 (m, 1H);

^{13}C{^{1}H} NMR (100 MHz, $CDCl_3$) δ 166.1, 165.9, 165.4, 165.1, 164.4, 133.9, 133.54, 133.50, 133.4, 133.1, 130.0, 129.89, 129.85, 129.79, 129.74, 129.5, 129.0, 128.8, 128.7, 128.5, 128.45, 128.42, 128.38, 90.0, 70.49, 70.47, 70.42, 68.8, 62.5.

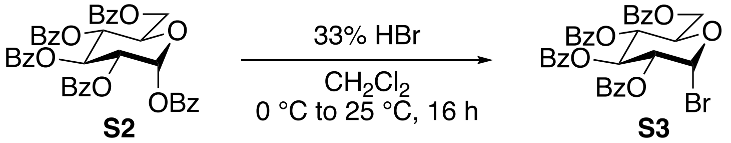

(2*R*,3*R*,4*S*,5*R*,6*R*)-2-((Benzoyloxy)methyl)-6-bromotetrahydro-2*H*-pyran-3,4,5-triyl tribenzoate (S3). A reported procedure[2] was adapted for the synthesis of glycosyl bromide intermediate **S3**. HBr (33 wt % in AcOH, 71 mL) was slowly added to a stirred solution of benzoylated glucose **S2** in CH_2Cl_2 (150 mL) at 0 °C. The reaction mixture was allowed to warm to 25 °C and was stirred for 16 h. The reaction mixture was poured over a stirring solution of ice water (150 mL) and the resulting biphasic mixture was stirred for 15 min. The layers were separated, and the aqueous layer was extracted with CH_2Cl_2 (2 × 150 mL). The combined organic layers were washed with saturated aqueous $NaHCO_3$ (1 × 150 mL) and brine (1 × 150 mL). The organic phase was dried over $MgSO_4$, filtered, and concentrated *in vacuo* to afford crude **S3**, which was used

directly without purification. The spectroscopic data are consistent with previously reported data for glycosyl bromide **S3**:[3]

^{1}H NMR (800 MHz, $CDCl_3$) δ 8.07 (dd, *J* = 8.3, 1.2 Hz, 1H), 8.00 (dd, *J* = 8.4, 1.3 Hz, 2H), 7.95 (dd, *J* = 8.4, 1.2 Hz, 2H), 7.88 (dd, *J* = 8.4, 1.2 Hz, 2H), 7.59–7.51 (m, 3H), 7.46–7.44 (m, 3H), 7.42–7.37 (m, 4H), 7.32–7.30 (m, 2H), 6.87 (d, *J* = 4.0 Hz, 1H), 6.26 (t, *J* = 9.8 Hz, 1H), 5.82 (t, *J* = 10.0 Hz, 1H), 5.33 (dd, *J* = 10.0, 4.0 Hz, 1H), 4.73 (ddd, *J* = 10.2, 4.3, 2.7 Hz, 1H), 4.67 (dd, *J* = 12.5, 2.6 Hz, 1H), 4.51 (dd, *J* = 12.5, 4.6 Hz, 1H);

^{13}C{^{1}H} NMR (201 MHz, $CDCl_3$) δ 166.0, 165.6, 165.3, 165.1, 133.8, 133.7, 133.4, 133.3, 130.1, 129.9, 129.8, 129.8, 128.6, 128.50, 128.47, 128.4, 86.9, 72.7, 71.5, 68.0, 61.9.

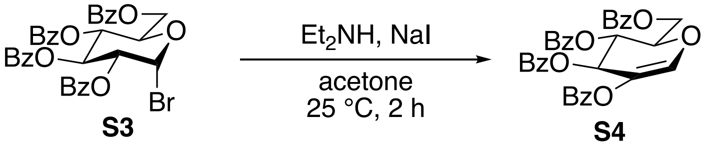

(2*R*,3*R*,4*S*)-2-((Benzoyloxy)methyl)-3,4-dihydro-2*H*-pyran-3,4,5-triyl tribenzoate (S4). A reported procedure[4] was adapted for the synthesis of glucal **S4**. NaI (5.10 g, 34.0 mmol) was added to a stirred solution of glycosyl bromide intermediate **S3** in acetone (30 mL) at 25 °C. The suspension was stirred at 25 °C for 15 min. Diethylamine (10.6 mL, 102 mmol) was added dropwise and the reaction mixture was stirred at 25 °C for 1 h. The reaction mixture was diluted with CH_2Cl_2 (60 mL). The organic phases were washed with H_2O (2 × 60 mL), 1 N HCl (2 × 30 mL), $NaHCO_3$ (1 × 30 mL), and H_2O (1 × 30 mL). The organic layer was dried over $MgSO_4$, filtered, and concentrated *in vacuo* to afford pure glucal **S4** as a tan solid (15.8 g, 80%). The spectroscopic data are consistent with previously reported data for glucal **S4**:[5]

^{1}H NMR (400 MHz, $CDCl_3$) δ 8.12–8.10 (m, 2H), 8.04–8.00 (m, 4H), 7.98–7.95 (m, 2H), 7.62–7.36 (m, 12H), 6.97 (s, 1H), 6.10 (d, *J* = 3.8 Hz, 1H), 5.83 (t, *J* = 4.1 Hz, 1H), 4.93–4.86 (m, 2H), 4.73–4.69 (m, 1H);

^{13}C{^{1}H} NMR (100 MHz, $CDCl_3$) δ 166.1, 165.5, 165.4, 165.0, 139.8, 133.6, 133.5, 133.4, 133.2, 130.09, 130.08, 129.8, 129.7, 128.6, 128.48, 128.47, 128.4, 127.4, 73.8, 68.2, 66.6, 61.5.

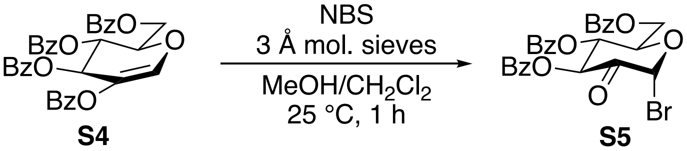

(2*R*,3*R*,4*S*,6*R*)-2-((Benzoyloxy)methyl)-6-bromo-5-oxotetrahydro-2*H*-pyran-3,4-diyl dibenzoate (S5). A reported procedure[6] was adapted for the synthesis of ketone **S5**. A solution of glucal **S4** (15.8 g, 27.3 mmol) in CH_2Cl_2 (275 mL) was added to a flask containing 3 Å molecular sieves and MeOH (1.3 mL) at 25 °C. The mixture was stirred at 25 °C for 30 min. *N*-Bromosuccinimide (5.83 g, 32.8 mmol) was added in a single

portion. The progress of the reaction was closely monitored by TLC, and the reaction was complete after stirring at 25 °C for 20 min. The reaction mixture was diluted with CH_2Cl_2 (100 mL) and saturated aqueous $Na_2S_2O_3$ (150 mL) was added. The layers were separated and the organic layer was washed with H_2O (2 × 100 mL). The organic layer was dried over $MgSO_4$, filtered, and concentrated *in vacuo* to afford ketone **S5** as an amorphous yellow solid, which was used immediately for the next step without purification. The spectroscopic data are consistent with previously reported data for ketone **S5**:[6]

^{1}H NMR (400 MHz, $CDCl_3$) δ 8.08–7.94 (m, 6H), 7.60–7.52 (m, 3H), 7.47–7.38 (m, 6H), 6.52 (d, *J* = 10.8 Hz, 1H), 6.50 (s, 1H), 6.03 (t, *J* = 10.4 Hz, 1H), 4.92 (ddd, *J* = 10.3, 4.1, 2.5 Hz, 1H), 4.75 (dd, *J* = 12.7, 2.5 Hz, 1H), 4.55 (dd, *J* = 12.7, 4.5 Hz, 1H);

^{13}C{^{1}H} NMR (100 MHz, $CDCl_3$) δ 188.4, 165.9, 165.1, 164.5, 133.9, 133.8, 133.4, 130.1, 130.0, 129.8, 128.6, 128.54, 128.51, 83.0, 73.04, 72.96, 68.2, 61.5.

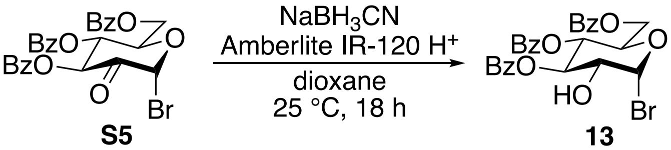

S5 13

(2*R*,3*R*,4*R*,5*R*,6*R*)-2-((Benzoyloxy)methyl)-6-bromo-5-hydroxytetrahydro-2*H*-pyran-3,4-diyl dibenzoate (13). A reported procedure[7] was adapted for the synthesis of alcohol **13**. Crude ketone **S5** was dissolved in dry 1,4-dioxane (150 mL). Amberlite® IR-120 (H^+ form, 15.3 g) and $NaBH_3CN$ (1.72 g, 27.3 mmol) were added at 25 °C. The reaction mixture was stirred at 25 °C for 18 h. The reaction mixture was filtered over Celite and the filtrate was concentrated *in vacuo*. Purification of the crude residue by flash chromatography (20:80 to 35:65 EtOAc/hexanes) afforded alcohol **13** as a white solid (3.76 g, 25% over two steps). The spectroscopic data are consistent with previously reported data for alcohol **13**:[7]

^{1}H NMR (400 MHz, $CDCl_3$) δ 8.06–8.04 (m, 2H), 7.98–7.94 (m, 4H), 7.60–7.50 (m, 3H), 7.46–7.35 (m, 6H), 6.64 (d, *J* = 3.8 Hz, 1H), 5.80–5.72 (m, 2H), 4.68–4.62 (m, 2H), 4.48 (dd, *J* = 12.8, 4.9 Hz, 1H), 3.94–3.88 (m, 1H), 2.63 (d, *J* = 10.6 Hz, 1H);

^{13}C{^{1}H} NMR (100 MHz, $CDCl_3$) δ 167.2, 166.0, 165.1, 133.7, 133.6, 133.3, 129.94, 129.88, 129.80, 129.5, 128.8, 128.52, 128.47, 94.3, 74.3, 72.9, 72.0, 67.2, 61.9.

Synthesis of Tetrahydropyranyl Alcohol 16

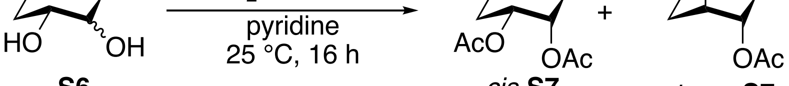

S6 *cis*-**S7** *trans*-**S7**

(2*R,3*R**)-Tetrahydro-2*H*-pyran-2,3-diyl diacetate (*cis*-S7) and (2*R**,3*S**)-tetrahydro-2*H*-pyran-2,3-diyl diacetate (*trans*-S7).** A reported procedure[8] was adapted for the

synthesis of diacetates *cis*-**S7** and *trans*-**S7**. To a stirred solution of diol **S6** (1.02 g, 8.67 mmol) in pyridine (9 mL) was added DMAP (0.0110 g, 0.0867 mmol) at 25 °C. Acetic anhydride (3.28 mL, 34.7 mmol) was added dropwise at 25 °C. The reaction mixture was stirred at 25 °C for 16 h. Pyridine was removed *in vacuo* and the resulting residue was dissolved in EtOAc (30 mL). The organic layer was washed with 1 N HCl (2 × 20 mL), saturated aqueous $NaHCO_3$ (2 × 20 mL), and brine (2 × 20 mL). The organic layer was dried over $MgSO_4$, filtered, and concentrated *in vacuo* to afford acetates *cis*-**S7** and *trans*-**S7** in a 25:75 ratio (0.948 g, 54%), which were used directly in the next step without purification. Characterization was performed on an analytical sample of an inseparable 25:75 mixture of diastereomers, which was obtained by purification with flash chromatography (10:90 EtOAc/hexanes):

Major diastereomer *trans*-**S7**:
^{1}H NMR (400 MHz, $CDCl_3$, distinctive peaks) δ 5.82 (d, *J* = 3.5 Hz, 1H), 4.75–4.72 (m, 1H), 3.89 (ddd, *J* = 11.6, 9.7, 3.1 Hz, 1H), 2.11 (s, 3H), 2.10 (s, 3H);
$^{13}C\{^1H\}$ NMR (100 MHz, $CDCl_3$) δ 170.05 (C), 169.2 (C), 91.4 (CH), 67.7 (CH), 62.8 (CH_2), 24.3 (CH_2), 21.1 (CH_3), 21.01 (CH_3), 20.8 (CH_2);

Minor diastereomer *trans*-**S7**:
^{1}H NMR (400 MHz, $CDCl_3$, distinctive peaks) δ 6.09 (d, *J* = 3.0 Hz, 1H), 4.95–4.91 (m, 1H), 3.80 (ddd, *J* = 11.4, 9.6, 4.8 Hz, 1H), 2.14 (s, 3H), 2.03 (s, 3H);
$^{13}C\{^1H\}$ NMR (100 MHz, $CDCl_3$) δ 170.13 (C), 169.6 (C), 90.1 (CH), 68.6 (CH), 61.5 (CH_2), 23.7 (CH_2), 23.6 (CH_2), 21.02 (CH_3), 20.95 (CH_3);

IR (neat) 2960, 1739, 1369, 1227, 1995, 1009, 938, 801 cm^{-1};

HRMS (ESI) *m* / *z* calcd for $C_9H_{14}NaO_5$ $(M+H)^+$ 225.0733, found 225.0728.

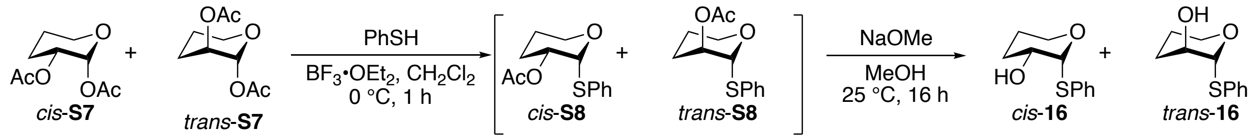

(2*R,3*R**)-2-(Phenylthio)tetrahydro-2*H*-pyran-3-yl acetate (*cis*-S8) and (2*R**,3*S**)-2-(phenylthio)tetrahydro-2*H*-pyran-3-yl acetate (*trans*-S8).** A reported procedure[9] was adapted for the synthesis of acetylated thiols *cis*-**S8** and *trans*-**S8**. To a stirred solution of diacetates *cis*-**S7** and *trans*-**S7** (0.948 g, 4.69 mmol) in CH_2Cl_2 (25 mL) at 0 °C were added thiophenol (0.526 mL, 5.16 mmol) and BF_3•OEt_2 (1.74 mL, 14.1 mmol). The reaction mixture was stirred at 0 °C for 1 h. Saturated aqueous $NaHCO_3$ (25 mL) was added and the layers were separated. The aqueous layer was extracted with CH_2Cl_2 (3 × 25 mL). The combined organic layers were dried over $MgSO_4$, filtered, and concentrated *in vacuo*. The crude acetylated thiols *cis*-**S8** and *trans*-**S8** were used immediately without further purification.
(2*R,3*R**)-2-(Phenylthio)tetrahydro-2*H*-pyran-3-ol (*cis*-16) and (2*R**,3*S**)-2-(phenylthio)tetrahydro-2*H*-pyran-3-ol (*trans*-16).** The crude acetylated thiols *cis*-**S8** and *trans*-**S8** were dissolved in MeOH (25 mL) and the resulting solution was stirred at 25 °C. To this stirring solution was added NaOMe (0.0013 g, 0.23 mmol). The reaction

mixture was stirred at 25 °C for 16 h. MeOH was removed *in vacuo* and the crude residue was purified by flash chromatography (20:80 EtOAc/hexanes) to afford alcohols *cis*-**16** and *trans*-**16** in a 34:66 ratio as a colorless oil (0.983 g, 100% over two steps). The relative stereochemistry of the products was determined by comparing the *J*-coupling constants of the anomeric protons as previously described[10]:

Major diastereomer *trans*-**16**:
^{1}H NMR (400 MHz, $CDCl_3$, distinctive peaks) δ 4.68 (d, *J* = 7.3 Hz, 1H), 2.39 (d, *J* = 3.7 Hz, 1H);
^{13}C{^{1}H} NMR (100 MHz, $CDCl_3$, distinctive peaks) δ 133.2 (C), 132.1 (CH), 128.99 (CH), 127.7 (CH), 91.5 (CH), 68.0 (CH), 66.7 (CH_2), 30.0 (CH_2), 23.6 (CH_2);

Minor diastereomer *cis*-**16**:
^{1}H NMR (400 MHz, $CDCl_3$, distinctive peaks) δ 5.35 (d, *J* = 3.6 Hz, 1H), 2.18 (d, *J* = 9.6 Hz, 1H);
^{13}C{^{1}H} NMR (100 MHz, $CDCl_3$, distinctive peaks) δ 134.7 (C), 131.6 (CH), 129.03 (CH), 127.3 (CH), 93.1 (CH), 68.5 (CH), 62.6 (CH_2), 29.8 (CH_2), 23.3 (CH_2);

IR (neat) 3046, 2941, 2861, 1478, 1438, 1046, 875 cm^{-1};

HRMS (ESI) *m* / *z* calcd for $C_{11}H_{14}NaO_2S$ $(M+H)^+$ 233.0607, found 233.0612.

Syntheses of Precursors of Authentic Samples of Rearrangement Products

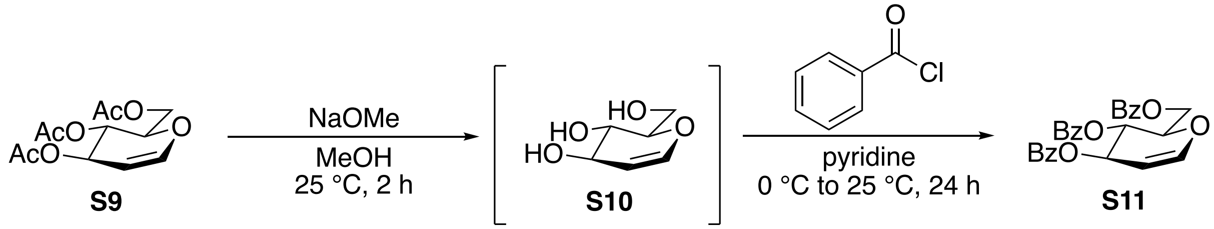

(2*R*,3*S*,4*R*)-2-(Hydroxymethyl)-3,4-dihydro-2*H*-pyran-3,4-diol (S10). NaOMe (0.002 g, 0.03 mmol) was added to a stirred solution of acetylated glucal **S9** (0.313 g, 1.15 mmol) in MeOH (2 mL) at 25 °C. The reaction mixture was stirred at 25 °C for 2 h. MeOH was removed *in vacuo*. The residue was filtered over a pad of silica with 30:70 acetone/diethyl ether and was concentrated *in vacuo* to afford triol **S10** as a colorless oil, which was used immediately without purification.

(2*R*,3*S*,4*R*)-2-((Benzoyloxy)methyl)-3,4-dihydro-2*H*-pyran-3,4-diyl dibenzoate (S11). The crude glucal **S10** (0.216 g, 1.15 mmol) was dissolved in pyridine (3 mL) and the solution was cooled to 0 °C. Benzoyl chloride (0.534 mL, 4.58 mmol) was added dropwise by syringe pump over a period of 30 min. The reaction mixture was allowed to warm to 25 °C and was stirred for 24 h. MeOH (1 mL) was added and the reaction mixture was stirred for an additional 15 min. The mixture was diluted with CH_2Cl_2 (10 mL). The organic layer was washed with 1 N aqueous HCl (1 × 10 mL), H_2O (1 × 10 mL), and brine (1 × 10 mL). The organic layer was dried over $MgSO_4$, filtered, and concentrated *in vacuo*. Purification by flash chromatography (15:85 EtOAc/hexanes) afforded glucal **S11** as a viscous, colorless oil (0.313 g, 59%). The spectroscopic data are consistent with previously reported data for glucal **S11**:[12]

^{1}H NMR (400 MHz, $CDCl_3$) δ 8.12–7.99 (m, 6H), 7.59–7.51 (m, 3H), 7.46–7.39 (m, 6H), 6.61 (dd, J = 6.2 1.2 Hz, 1H), 5.82–5.79 (m, 1H), 5.73–5.70 (m, 1H), 5.12 (dd, J = 6.2, 3.5 Hz, 1H), 4.71–4.68 (m, 3H);

^{13}C{^{1}H} NMR (100 MHz, $CDCl_3$) δ 166.2, 165.8, 165.1, 145.9, 133.5, 133.3, 133.2, 129.9, 129.8, 129.7, 129.57, 129.54, 129.53, 128.49, 128.46, 128.42, 98.8, 73.9, 67.8, 67.5, 62.1.

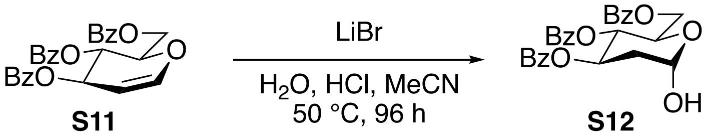

(2*R*,3*S*,4*R*,6*S*)-2-((Benzoyloxy)methyl)-6-hydroxytetrahydro-2*H*-pyran-3,4-diyl dibenzoate (S12). A reported procedure[13] was adapted for the synthesis of hemiacetal **S12**. To a stirred solution of glucal **S11** (0.313 g, 0.683 mmol) and LiBr (0.184 g, 2.12 mmol) in a mixture of H_2O (0.22 mL) and MeCN (6.0 mL) at 0 °C was added concentrated HCl (0.010 mL). The reaction mixture was heated to 50 °C in an oil bath and was stirred for 96 h. The reaction mixture was cooled to 25 °C and saturated aqueous $NaHCO_3$ (10 mL) was added. The biphasic mixture was concentrated *in vacuo* to remove MeCN. The remaining aqueous residue was extracted with CH_2Cl_2 (3 × 10 mL). The organic layers were combined, dried over $MgSO_4$, filtered, and concentrated *in vacuo* to afford hemiacetal **S12** as a white foam (0.200 g, 61%). The spectroscopic data are consistent with previously reported data for hemiacetal **S12**:[13]

^{1}H NMR (400 MHz, $CDCl_3$) δ 8.06–8.02 (m, 3H), 7.98–7.93 (m, 3H), 7.56–7.48 (m, 4H), 7.44–7.35 (m, 5H), 5.83–5.78 (m, 1H), 5.62 (t, J = 9.9 Hz, 1H), 5.54 (app s, 1H), 4.64–4.60 (m, 2H), 4.48–4.42 (m, 1H), 3.08 (br s, 1H), 2.56 (dd, J = 12.9, 5.0 Hz, 1H), 2.04–1.98 (m, 1H);

^{13}C{^{1}H} NMR (100 MHz, $CDCl_3$) δ 166.3, 165.8, 165.6, 133.3, 133.09, 133.05, 129.8 (2 CH overlap), 129.7, 128.40, 128.35 (2 CH overlap), 91.9, 70.3, 69.7, 68.4, 63.3, 35.4.

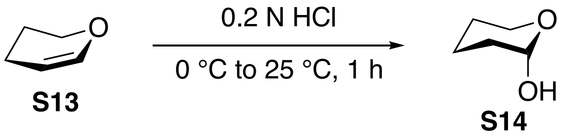

Tetrahydro-2*H*-pyran-2-ol (S14). Dihydropyran **S13** (0.300 mL, 3.29 mmol) was added to a stirred solution of 0.2 N HCl (1 mL) at 0 °C. The reaction mixture was warmed to 25 °C and was stirred for 1 h. The reaction mixture was extracted with CH_2Cl_2 (3 × 1 mL). The combined organic layers were washed with saturated aqueous $NaHCO_3$ (1 × 2 mL) and brine (1 × 2 mL), dried over $MgSO_4$, filtered, and concentrated *in vacuo* to afford hemiacetal **S14** as a colorless oil (0.0534 g, 16%). The spectroscopic data are consistent with previously reported data for hemiacetal **S14**.[14]

^{1}H NMR (400 MHz, $CDCl_3$) δ 4.91–4.88 (m, 1H), 4.04–3.99 (m, 1H), 3.56–3.50 (m, 1H), 2.96 (br s, 1H), 1.89–1.74 (m, 2H), 1.59–1.47 (m, 4H);

^{13}C{^{1}H} NMR (100 MHz, $CDCl_3$) δ 94.4, 63.9, 31.9, 25.2, 20.2.

1,2-Spin-Center Shift Reactions of Radical Clocks

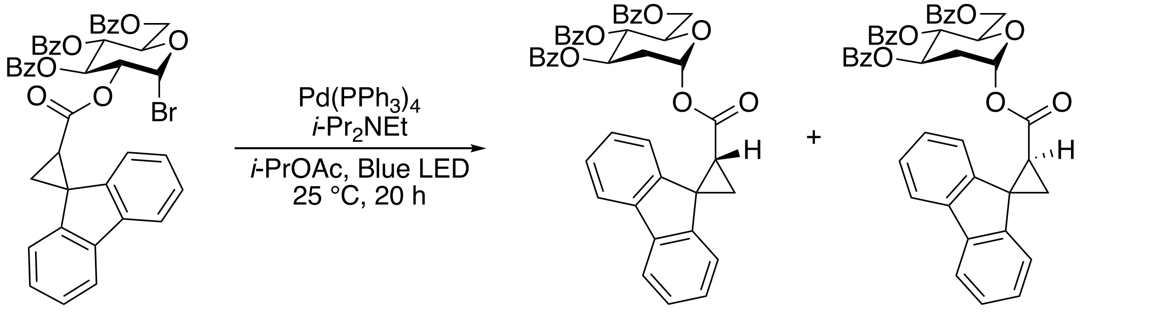

15 **18a** **18b**

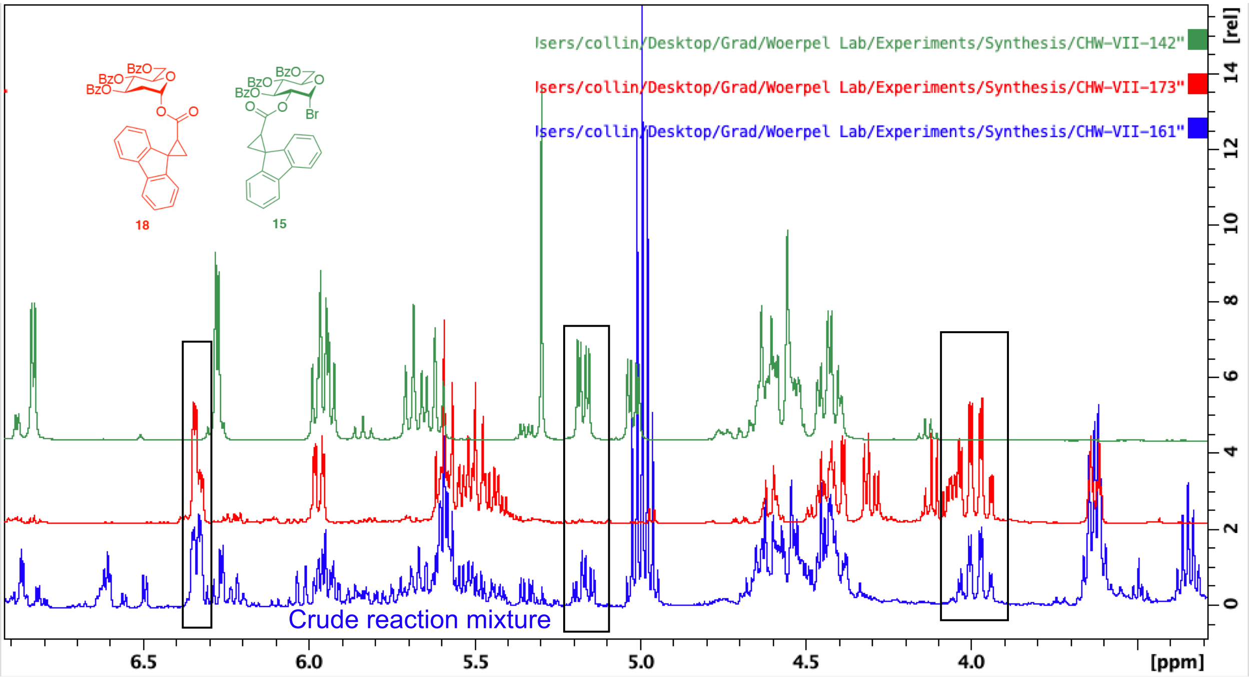

Figure S1: Crude ^{1}H spectrum (from δ 7.0 to 3.3 ppm, 400 MHz) of 1,2-SCS of glycosyl bromide **15** to form rearranged product **18**. Diagnostic overlapping signals between the authentic sample and the crude spectra are highlighted with black boxes.

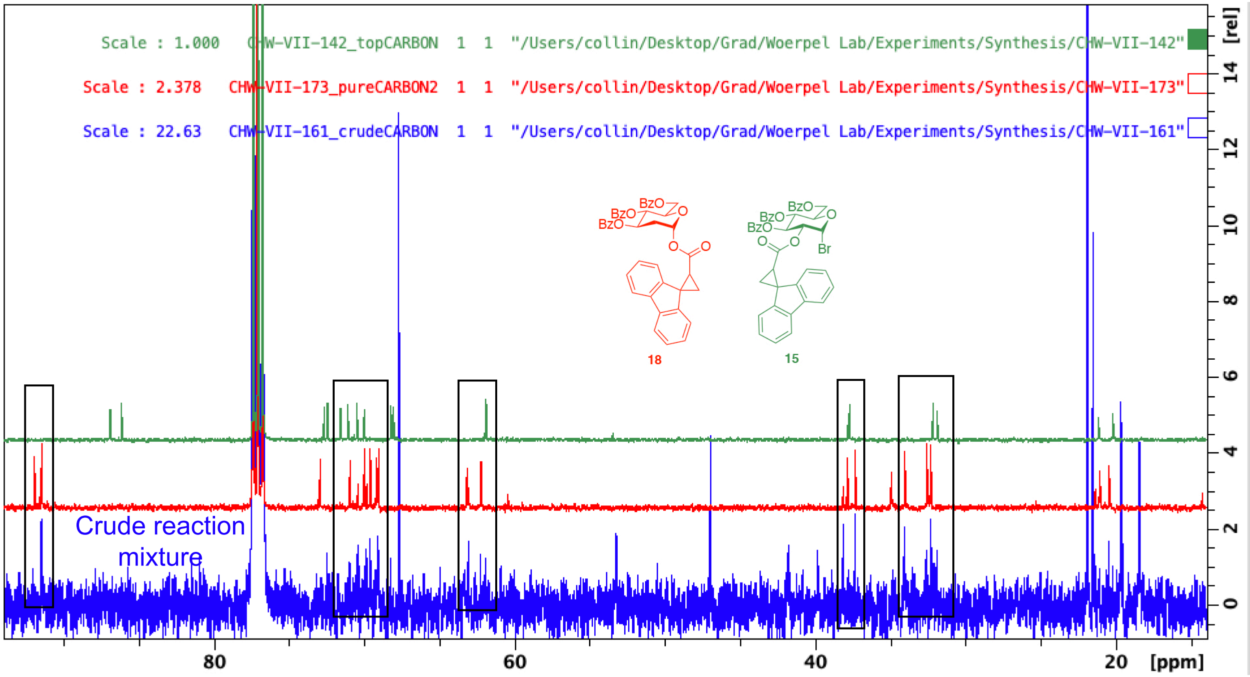

Figure S2: Crude $^{13}C\{^{1}H\}$ spectrum (from δ 95 to 15 ppm, 100 MHz) of 1,2-SCS of glycosyl bromide **15** to form rearranged product **18**. Diagnostic overlapping signals between the authentic sample and the crude spectra are highlighted with black boxes.

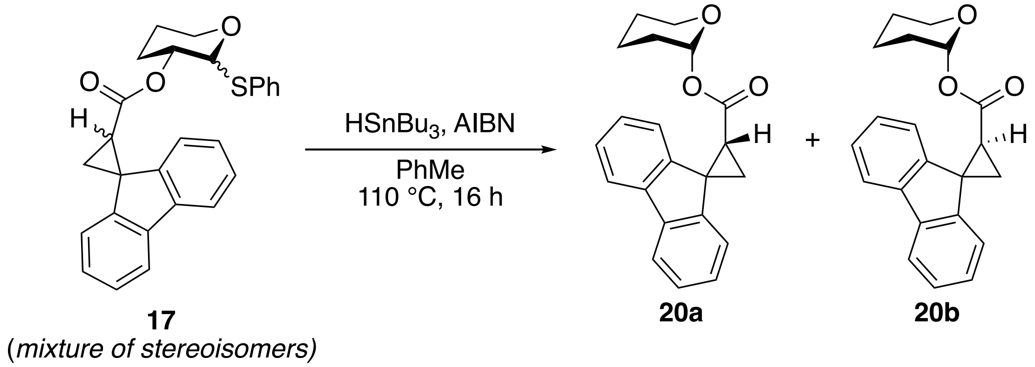

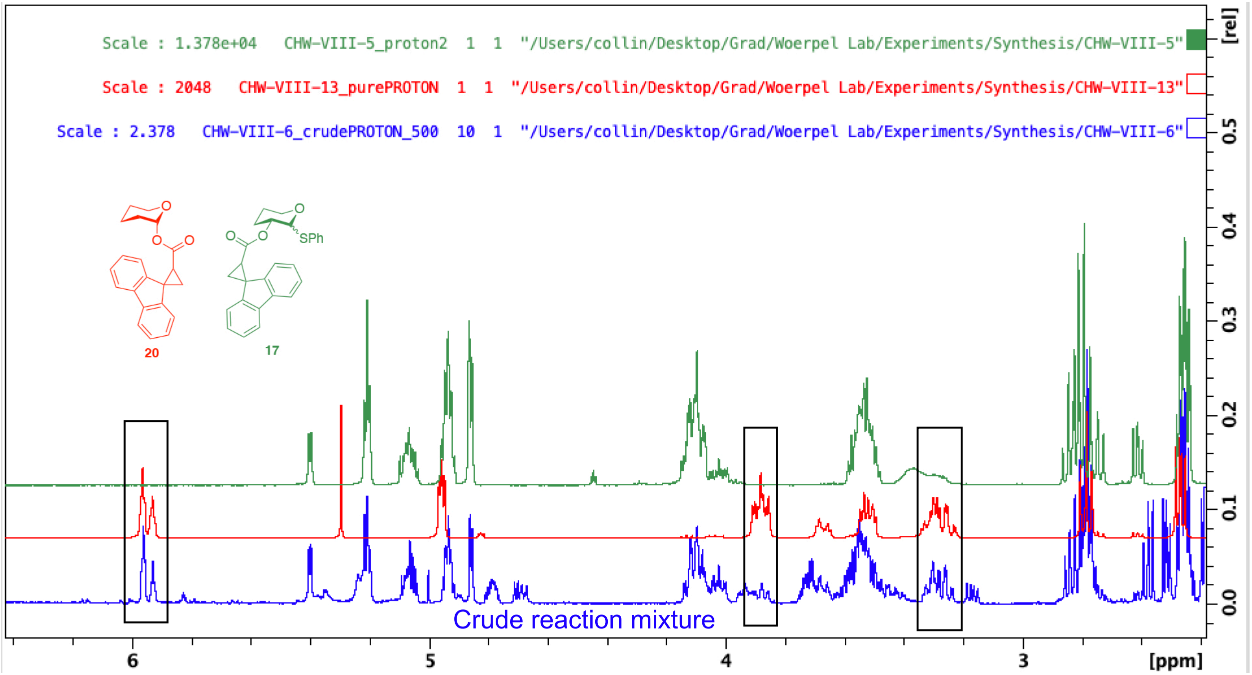

Figure S3: Crude ^{1}H spectrum (from δ 6.5 to 3.4 ppm, 500 MHz) of 1,2-SCS of thiol **17** to form rearranged product **20**. Diagnostic overlapping signals between the authentic sample and the crude spectra are highlighted with black boxes.

Methanolysis of 1,2-Spin-Center Shift Rearrangement Product 18

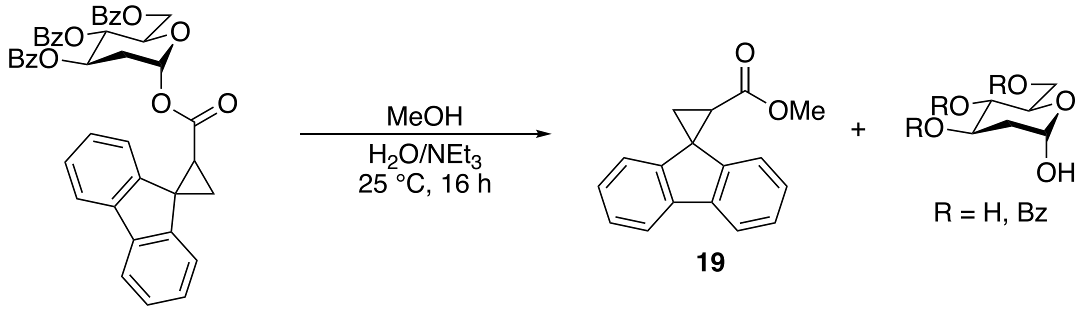

18
(*obtained from 1,2-SCS* of **15**)

Methyl spiro[cyclopropane-1,9'-fluorene]-2-carboxylate (19). Rearranged product **18** (0.0435, 0.0626 mmol), which was chromatographically separated following the 1,2-spin-center shift of radical clock **15**, was dissolved in a mixture of MeOH (2.8 mL), H_2O (0.80 mL), and triethylamine (0.40 mL). The reaction mixture was stirred vigorously at 25 °C for 16 h. The reaction mixture was concentrated *in vacuo*. Purification by flash chromatography (0:100 to 5:95 MeOH/CH_2Cl_2) afforded methyl ester **19** as a white solid (0.0081 g, 55%) and a polar fraction of partially deprotected C2-deoxy glucose derivatives. The spectroscopic data for methyl ester **19** are consistent with previously reported data for this compound.[11] The isolation of C2-deoxy sugars is evident by the HSQC spectrum for this mixture of compounds, which indicates the presence of upfield (δ 2.25–2.02 ppm) methylene proton signals that would not be present in the product of methanolysis of non-rearranged direct C1 reduction products (Figure S4). The presence of a rearranged carbohydrate scaffold and an unopened radical clock provides further evidence that the 1,2-SCS proceeds without the intermediacy of a dioxolanyl radical:

Methyl ester **19**:

^{1}H NMR (400 MHz, $CDCl_3$) δ 7.82–7.79 (m, 2H), 7.59 (d, *J* = 7.7 Hz, 1H), 7.40–7.37 (m, 2H), 7.33–7.27 (m, 2H), 7.04 (d, *J* = 7.5 Hz, 1H), 3.64 (s, 3H), 2.76 (dd, *J* = 8.3, 7.7 Hz, 1H), 2.46 (dd, *J* = 7.5, 5.2 Hz, 1H), 2.14 (dd, *J* = 8.4, 5.2 Hz, 1H);
^{13}C{^{1}H} NMR (100 MHz, $CDCl_3$) δ 169.9, 146.4, 142.5, 141.0, 139.8, 127.1, 127.0, 126.9, 122.8, 119.87, 119.85, 118.6, 52.0, 37.0, 32.7, 20.9.

HSQC for rearranged sugars:

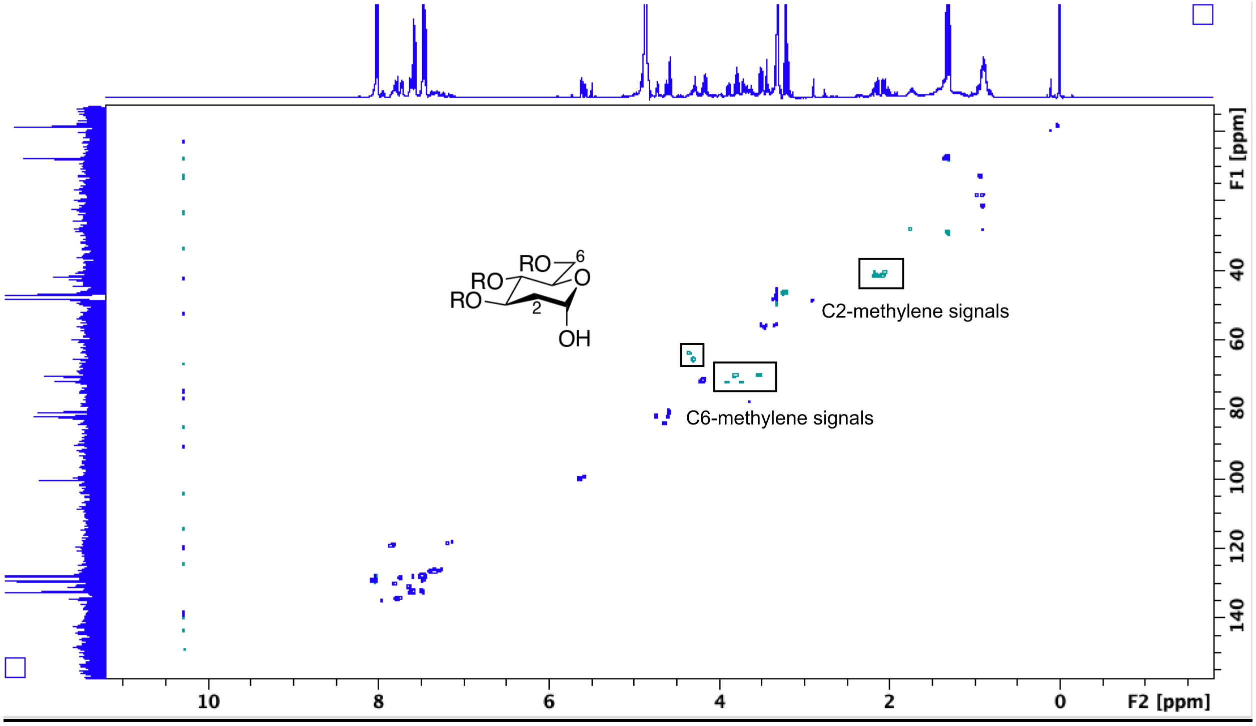

Figure S4: HSQC spectrum for the polar fraction of methanolysis of 1,2-SCS rearranged product **18**, which shows the distinctive upfield ^{1}H and ^{13}C methylene resonances for a C2-deoxy sugar.

References

(1) Otte, D. A.; Borchmann, D. E.; Lin, C.; Weck, M.; Woerpel, K. A. ^{13}C NMR Spectroscopy for the Quantitative Determination of Compound Ratios and Polymer End Groups. *Org. Lett.* **2014**, *16*, 1566–1569.

(2) Doyle, L. M.; O'Sullivan, S.; Di Salvo, C.; McKinney, M.; McArdle, P.; Murphy, P. V. Stereoselective Epimerizations of Glycosyl Thiols. *Org. Lett.* **2017**, *19*, 5802–5805.

(3) Zhao, G.; Yao, W.; Mauro, J. N.; Ngai, M.-Y. Excited-State Palladium-Catalyzed 1,2-Spin-Center Shift Enables Selective C-2 Reduction, Deuteration, and Iodination of Carbohydrates. *J. Am. Chem. Soc.* **2021**, *143*, 1728–1734.

(4) Brehm, M.; Göckel, V. H.; Jarglis, P.; Lichtenthaler, F. W. Expedient Conversion of D-Glucose into 1,5-Anhydro-D-Fructose and into Single Stereogenic-Center Dihydropyranones, Suitable Six-Carbon Scaffolds for Concise Syntheses of the Soft-Coral Constituents (−)-bissetone and (−)-palythazine. *Tetrahedron: Asymmetry* **2008**, *19*, 358–373.

(5) Khan, K. M.; Perveen, S.; Al-Qawasmeh, R. A. S.; Shekhani, M. S.; Ali Shah, S. T.; Voelter, W. A Method for the Syntheses of Enopyranosides. *Lett. Org. Chem.* **2009**, *6*, 191–196.

(6) Lichtenthaler, F. W.; Kläres, U.; Lergenmüller, M.; Schwidetzky, S. Various Glycosyl Donors with a Ketone or Oxime Function next to the Anomeric Centre: Facile Preparation and Evaluation of their Selectivities in Glycosidations. *Synthesis* **1992**, 179–184.
(7) Lichtenthaler, F. W.; Köhler, B. An Expedient Route to Acylated Glucosyl Halides with a Free 2-OH Group. *Carbohydr. Res.* **1994**, *258*, 77–85.
(8) Long, D. E.; Karmakar, P.; Wall, K. A.; Sucheck, S. J. Synthesis of α-L-Rhamnosyl Ceramide and Evaluation of its Binding with Anti-Rhamnose Antibodies. *Bioorg. Med. Chem.* **2014**, *22*, 5279–5289.
(9) Kurosu, M.; Li, K.; Crick, D. C. Concise Synthesis of Capuramycin. *Org. Lett.* **2009**, *11*, 2393–2396.
(10) Beckwith, A. L. J.; Duggan, P. J. The Quasi-Homo-Anomeric Interaction in Substituted Tetrahydropyranyl Radicals: Structure and Kinetics of Formation. *Tetrahedron* **1998**, *54*, 4623–4632.
(11) Bartolo, N. D.; Woerpel, K. A. Evidence Against Single-Electron Transfer in the Additions of Most Organomagnesium Reagents to Carbonyl Compounds. *J. Org. Chem.* **2020**, *85*, 7848–7862.
(12) Chen, H.; Xian, T.; Zhang, W.; Si, W.; Luo, X.; Zhang, B.; Zhang, M.; Wang, Z.; Zhang, J. An Efficient Method for the Synthesis of Pyranoid Glycals. *Carbohydr. Res.* **2016**, *431*, 42–46.
(13) Ghosh, T.; Mukherji, A.; Srivastava, H. K.; Kancharla, P. K. Secondary Amine Salt Catalyzed Controlled Activation of 2-Deoxy Sugar Lactols Towards Alpha-Selective Dehydrative Glycosylation. *Org. Biomol. Chem.* **2018**, *16*, 2870–2875.
(14) Clayman, P. D.; Hyster, T. K. Photoenzymatic Generation of Unstabilized Alkyl Radicals: An Asymmetric Reductive Cyclization. *J. Am. Chem. Soc.* **2020**, *142*, 15673–15677.
(15) Mayoralas-Fernandez, A.; Marra, A.; Trumtel, M.; Veyriéres, A.; Sinaÿ, P. Preparation of Pyranoid Glycal Derivatives from Phenyl Thioglycosides and Glycosyl Phenyl Sulphones. *Carbohydr. Res.* **1989**, *188*, 81–95.

^{1}H and ^{13}C{^{1}H} NMR Spectra for New Compounds

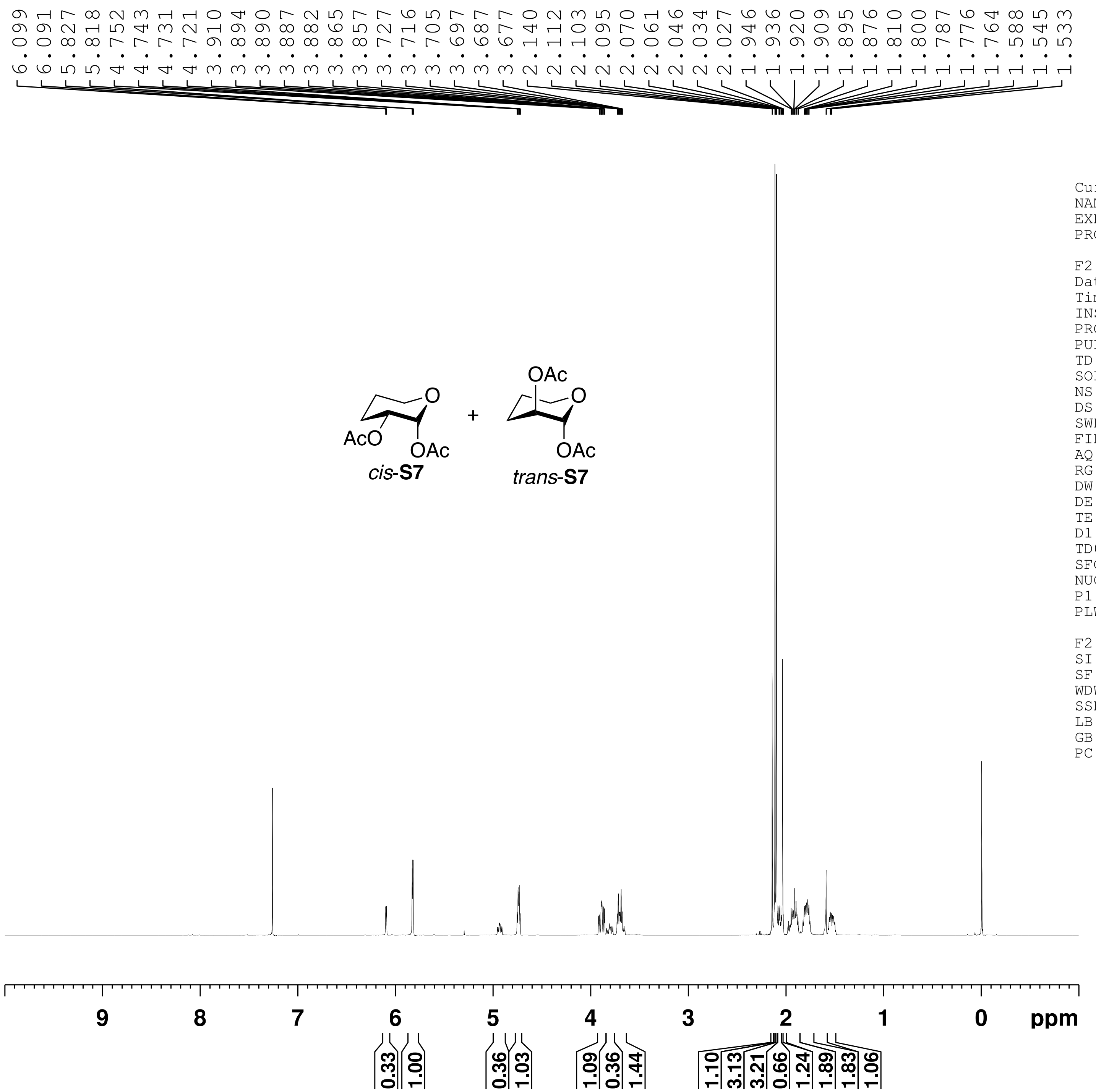


```
Current Data Parameters
NAME     CHW-VIII-1_purePROTON
EXPNO                   1
PROCNO                  1

F2 - Acquisition Parameters
Date_          20230221
Time              16.01 h
INSTRUM           spect
PROBHD   Z150354_0001 (
PULPROG            zg30
TD                65536
SOLVENT           CDCl3
NS                   16
DS                    2
SWH            8012.820 Hz
FIDRES         0.244532 Hz
AQ            4.0894465 sec
RG               117.47
DW               62.400 usec
DE                30.00 usec
TE                298.0 K
D1           1.00000000 sec
TD0                   1
SFO1        400.3024719 MHz
NUC1                 1H
P1                12.00 usec
PLW1         4.64209986 W

F2 - Processing parameters
SI                65536
SF          400.3000096 MHz
WDW                  EM
SSB                   0
LB                 0.30 Hz
GB                    0
PC                 1.00
```

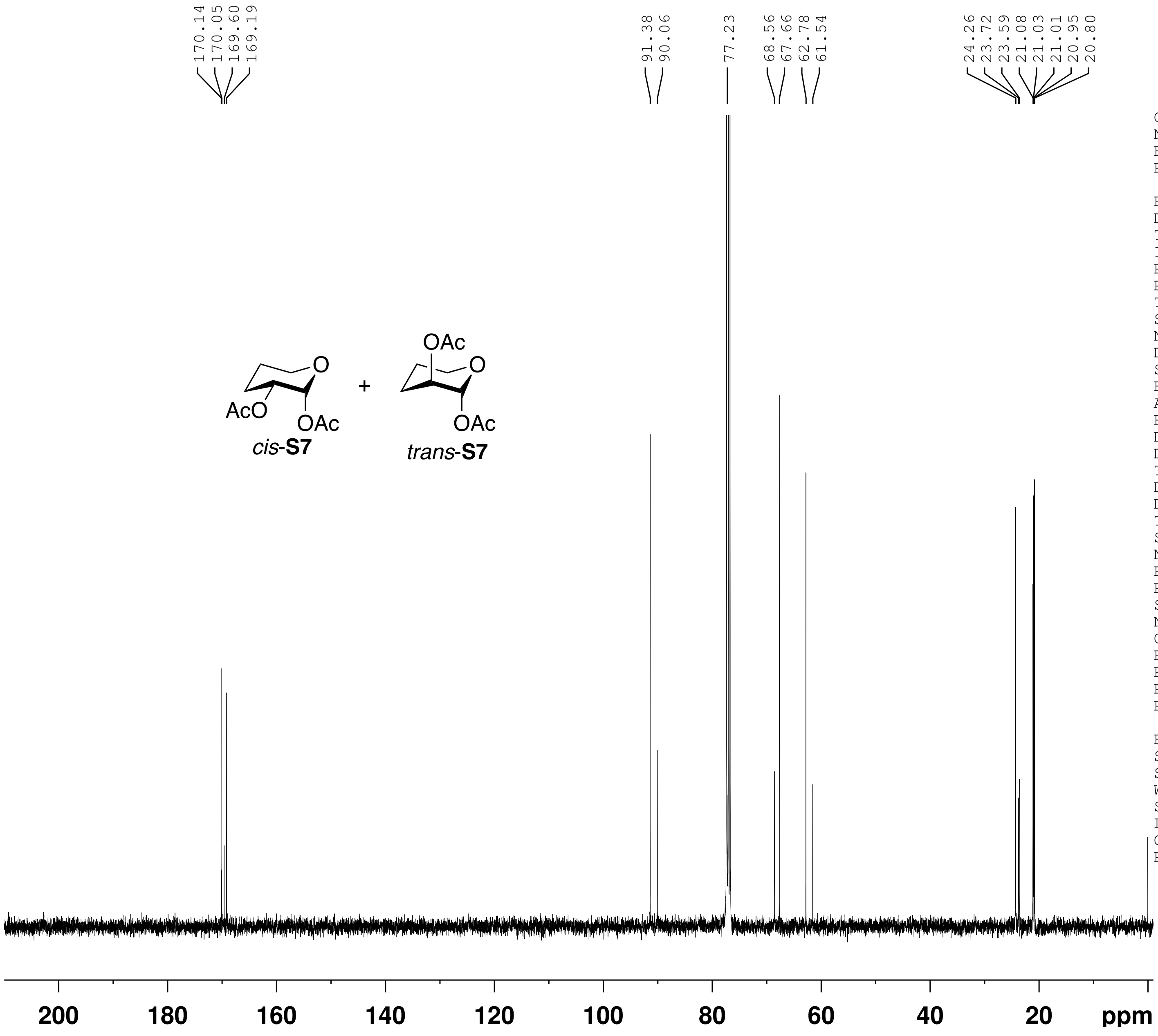

cis-**S7** + *trans*-**S7**

Current Data Parameters	
NAME	CHW-VIII-1_pureCARBON
EXPNO	1
PROCNO	1
F2 - Acquisition Parameters	
Date_	20230221
Time	18.38 h
INSTRUM	spect
PROBHD	Z150354_0001 (
PULPROG	zgpg30
TD	65356
SOLVENT	CDCl3
NS	128
DS	4
SWH	24038.461 Hz
FIDRES	0.735616 Hz
AQ	1.3594048 sec
RG	25.77
DW	20.800 usec
DE	25.00 usec
TE	298.0 K
D1	2.00000000 sec
D11	0.03000000 sec
TD0	1
SFO1	100.6655806 MHz
NUC1	13C
P1	10.00 usec
PLW1	17.99699974 W
SFO2	400.3016012 MHz
NUC2	1H
CPDPRG[2	waltz16
PCPD2	80.00 usec
PLW2	4.64209986 W
PLW12	0.10445000 W
PLW13	0.05245300 W
F2 - Processing parameters	
SI	32768
SF	100.6555148 MHz
WDW	EM
SSB	0
LB	1.00 Hz
GB	0
PC	1.40

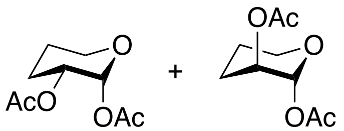

cis-**S7** + *trans*-**S7**

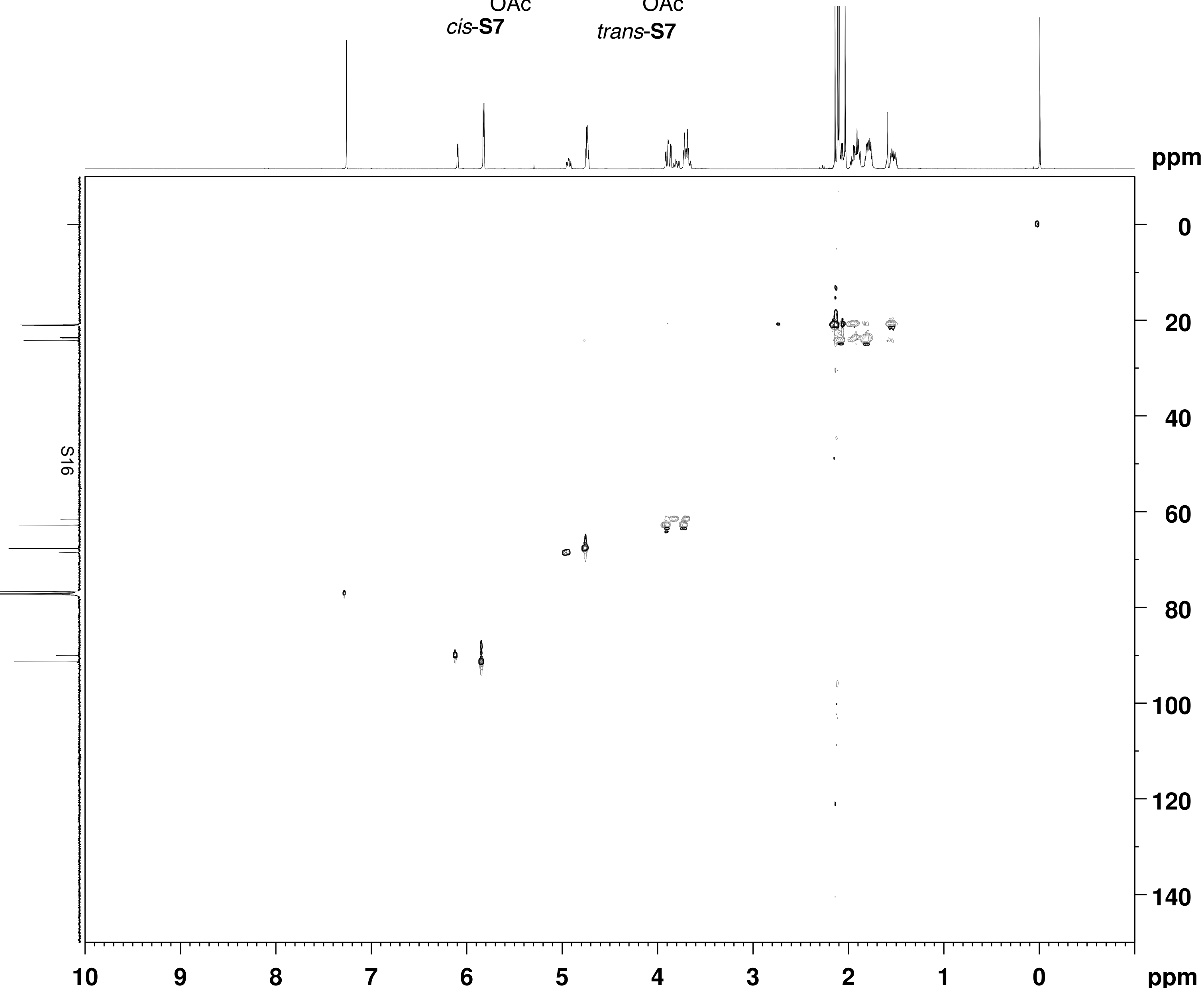


```
Current Data Parameters
NAME      CHW-VIII-1_hsqc
EXPNO                   1
PROCNO                  1

F2 - Acquisition Parameters
Date_            20230221
Time                19.31 h
INSTRUM             spect
PROBHD   Z150354_0001 (
PULPROG  hsqcedetgpsisp2.3
TD                   2048
SOLVENT             CDC13
NS                      2
DS                     32
SWH              5208.333 Hz
FIDRES           5.086263 Hz
AQ              0.1966080 sec
RG                 207.85
DW                 96.000 usec
DE                  30.00 usec
TE                  298.0 K
CNST2         145.0000000
CNST17         -0.5000000
D0             0.00000300 sec
D1             1.50000000 sec
D4             0.00172414 sec
D11            0.03000000 sec
D16            0.00020000 sec
D21            0.00344800 sec
D24            0.00086200 sec
IN0            0.00003010 sec
TDav                    1
SFO1          400.3018814 MHz
NUC1                   1H
P1                  12.00 usec
P2                  24.00 usec
P28                     0 usec
PLW1           4.64209986 W
SFO2          100.6630642 MHz
NUC2                  13C
CPDPRG[2            garp4
P3                  10.00 usec
P14                500.00 usec
P24               2000.00 usec
P31               2119.00 usec
PCPD2               65.00 usec
PLW0                    0 W
PLW2          17.99699974 W
PLW12          0.42594999 W
SPNAM[3] Crp60,0.5,20.1
SPOAL3              0.500
SPOFFS3                 0 Hz
SPW3           2.74970007 W
SPNAM[7]     Crp60comp.4
SPOAL7              0.500
SPOFFS7                 0 Hz
SPW7           2.74970007 W
SPNAM[18  Crp60_xfilt.2
SPOAL18             0.500
SPOFFS18                0 Hz
SPW18          0.52970999 W
GPNAM[1]      SMSQ10.100
GPZ1                80.00 %
GPNAM[2]      SMSQ10.100
GPZ2                20.10 %
GPNAM[3]      SMSQ10.100
GPZ3                11.00 %
GPNAM[4]      SMSQ10.100
GPZ4                -5.00 %
P16               1000.00 usec
P19                600.00 usec

F1 - Acquisition parameters
TD                    256
SFO1             100.6631 MHz
FIDRES         129.775742 Hz
SW                165.019 ppm
FnMODE    Echo-Antiecho

F2 - Processing parameters
SI                   1024
SF            400.3000000 MHz
WDW                 QSINE
SSB                     2
LB                      0 Hz
GB                      0
PC                   1.40

F1 - Processing parameters
SI                   1024
MC2          echo-antiecho
SF            100.6555151 MHz
WDW                 QSINE
SSB                     2
LB                      0 Hz
GB                      0
```

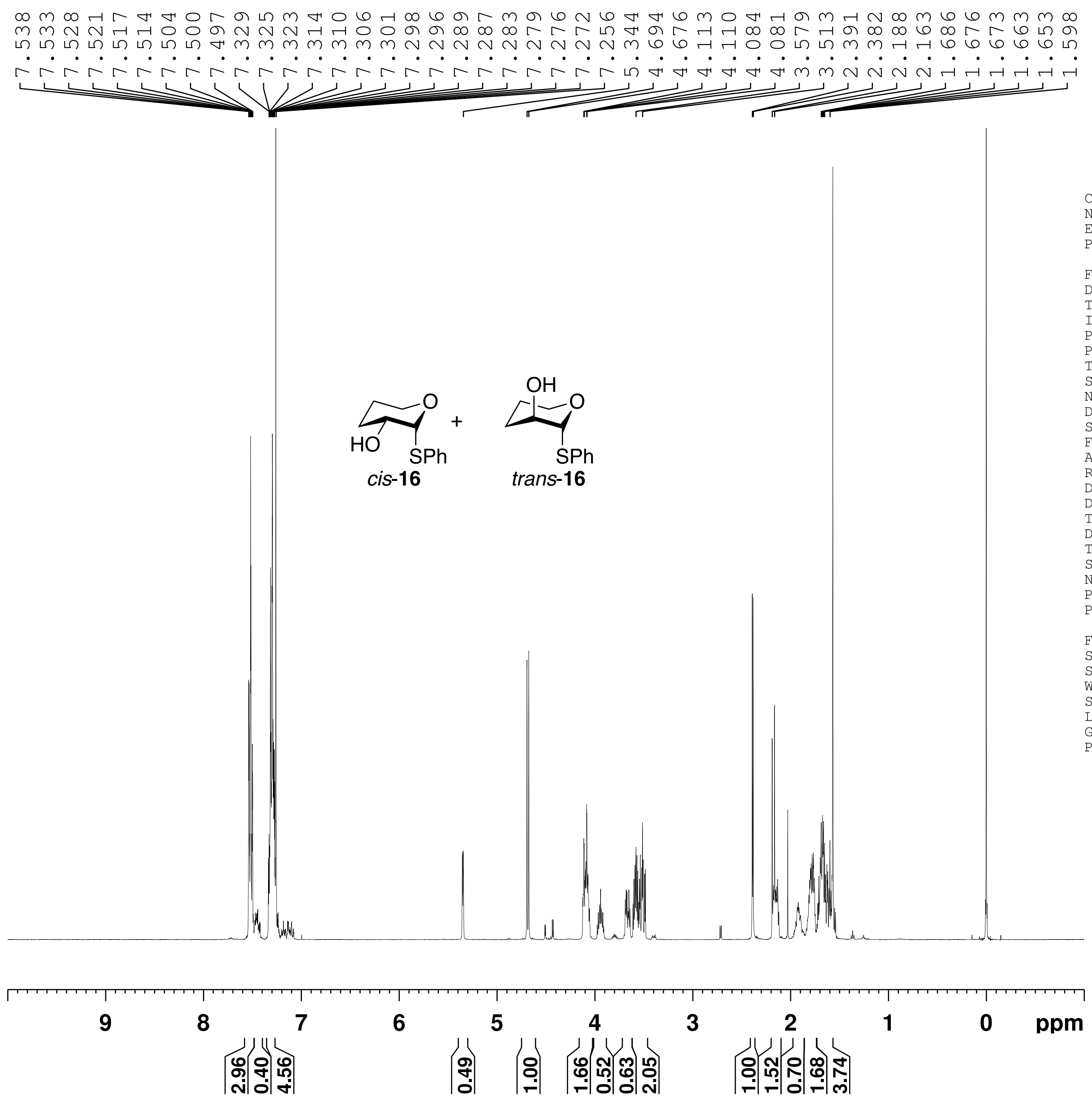


```
Current Data Parameters
NAME     CHW-VIII-4_purePROTON2
EXPNO                   1
PROCNO                  1

F2 - Acquisition Parameters
Date_          20230221
Time              11.37 h
INSTRUM           spect
PROBHD   Z150354_0001 (
PULPROG            zg30
TD                65536
SOLVENT           CDCl3
NS                   16
DS                    2
SWH            8012.820 Hz
FIDRES         0.244532 Hz
AQ            4.0894465 sec
RG               125.92
DW               62.400 usec
DE                30.00 usec
TE                298.0 K
D1           1.00000000 sec
TD0                   1
SFO1        400.3024719 MHz
NUC1                 1H
P1                12.00 usec
PLW1         4.64209986 W

F2 - Processing parameters
SI                65536
SF          400.3000095 MHz
WDW                  EM
SSB                   0
LB                 0.30 Hz
GB                    0
PC                 1.00
```

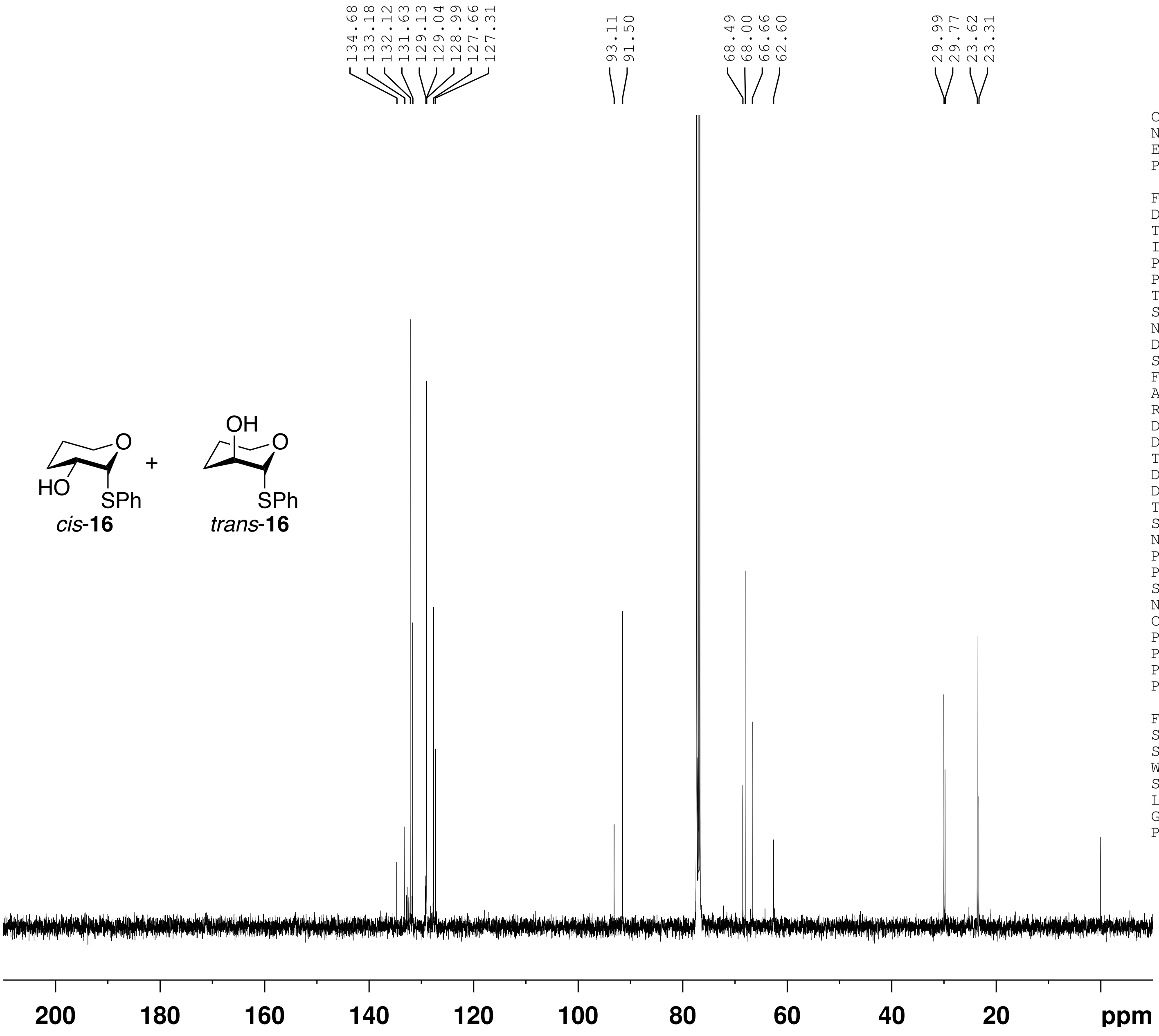


```
Current Data Parameters
NAME     CHW-VIII-4_pureCARBON2
EXPNO                 1
PROCNO                1

F2 - Acquisition Parameters
Date_          20230221
Time              12.57 h
INSTRUM           spect
PROBHD   Z150354_0001 (
PULPROG         zgpg30
TD               65356
SOLVENT          CDCl3
NS                 128
DS                   4
SWH          24038.461 Hz
FIDRES        0.735616 Hz
AQ           1.3594048 sec
RG               51.78
DW              20.800 usec
DE               25.00 usec
TE               298.0 K
D1          2.00000000 sec
D11         0.03000000 sec
TD0                  1
SFO1      100.6655806 MHz
NUC1               13C
P1               10.00 usec
PLW1       17.99699974 W
SFO2      400.3016012 MHz
NUC2                1H
CPDPRG[2       waltz16
PCPD2            80.00 usec
PLW2        4.64209986 W
PLW12       0.10445000 W
PLW13       0.05245300 W

F2 - Processing parameters
SI               32768
SF        100.6555157 MHz
WDW                 EM
SSB                  0
LB                1.00 Hz
GB                   0
PC                1.40
```

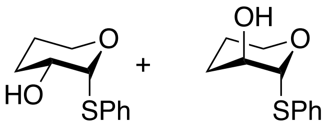

cis-**16** + *trans*-**16**

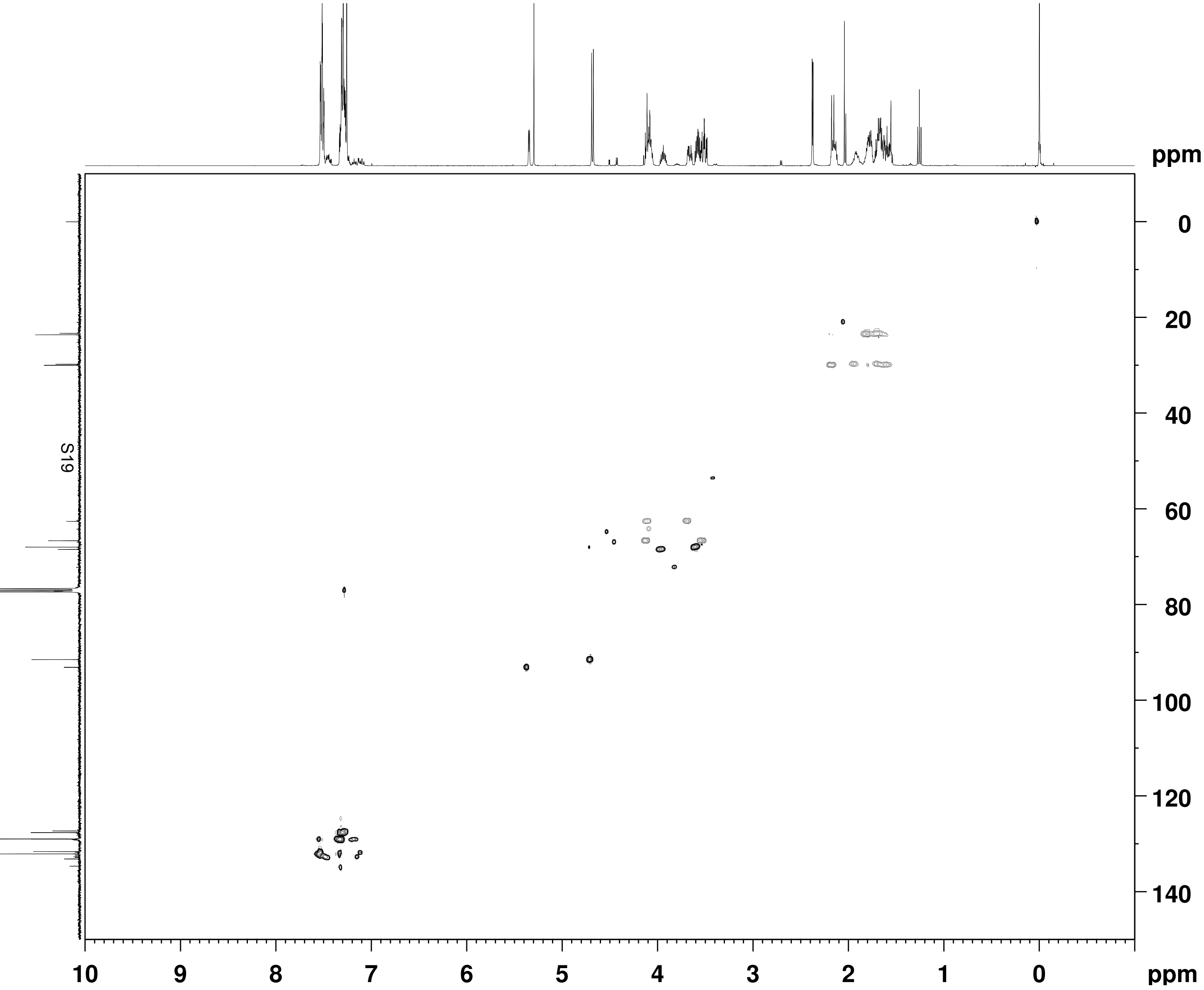


```
Current Data Parameters
NAME     CHW-VIII-4_hsqc
EXPNO              2
PROCNO             1

F2 - Acquisition Parameters
Date_          20230221
Time              13.28 h
INSTRUM           spect
PROBHD   Z150354_0001 (
PULPROG  hsqcedetgpsisp2.3
TD                 2048
SOLVENT            CDCl3
NS                    2
DS                   32
SWH            5208.333 Hz
FIDRES         5.086263 Hz
AQ            0.1966080 sec
RG               207.85
DW               96.000 usec
DE                30.00 usec
TE                298.0 K
CNST2       145.0000000
CNST17       -0.5000000
D0           0.00000300 sec
D1           1.50000000 sec
D4           0.00172414 sec
D11          0.03000000 sec
D16          0.00020000 sec
D21          0.00344800 sec
D24          0.00086200 sec
IN0          0.00003010 sec
TDav                  1
SFO1        400.3018814 MHz
NUC1                 1H
P1                12.00 usec
P2                24.00 usec
P28                   0 usec
PLW1         4.64209986 W
SFO2        100.6630642 MHz
NUC2                13C
CPDPRG[2          garp4
P3                10.00 usec
P14              500.00 usec
P24             2000.00 usec
P31             2119.00 usec
PCPD2             65.00 usec
PLW0                  0 W
PLW2        17.99699974 W
PLW12        0.42594999 W
SPNAM[3] Crp60,0.5,20.1
SPOAL3            0.500
SPOFFS3               0 Hz
SPW3         2.74970007 W
SPNAM[7]    Crp60comp.4
SPOAL7            0.500
SPOFFS7               0 Hz
SPW7         2.74970007 W
SPNAM[18  Crp60_xfilt.2
SPOAL18           0.500
SPOFFS18              0 Hz
SPW18        0.52970999 W
GPNAM[1]     SMSQ10.100
GPZ1              80.00 %
GPNAM[2]     SMSQ10.100
GPZ2              20.10 %
GPNAM[3]     SMSQ10.100
GPZ3              11.00 %
GPNAM[4]     SMSQ10.100
GPZ4              -5.00 %
P16             1000.00 usec
P19              600.00 usec

F1 - Acquisition parameters
TD                  256
SFO1           100.6631 MHz
FIDRES       129.775742 Hz
SW              165.019 ppm
FnMODE     Echo-Antiecho

F2 - Processing parameters
SI                 1024
SF          400.3000000 MHz
WDW               QSINE
SSB                   2
LB                    0 Hz
GB                    0
PC                 1.40

F1 - Processing parameters
SI                 1024
MC2        echo-antiecho
SF          100.6555151 MHz
WDW               QSINE
SSB                   2
LB                    0 Hz
GB                    0
```

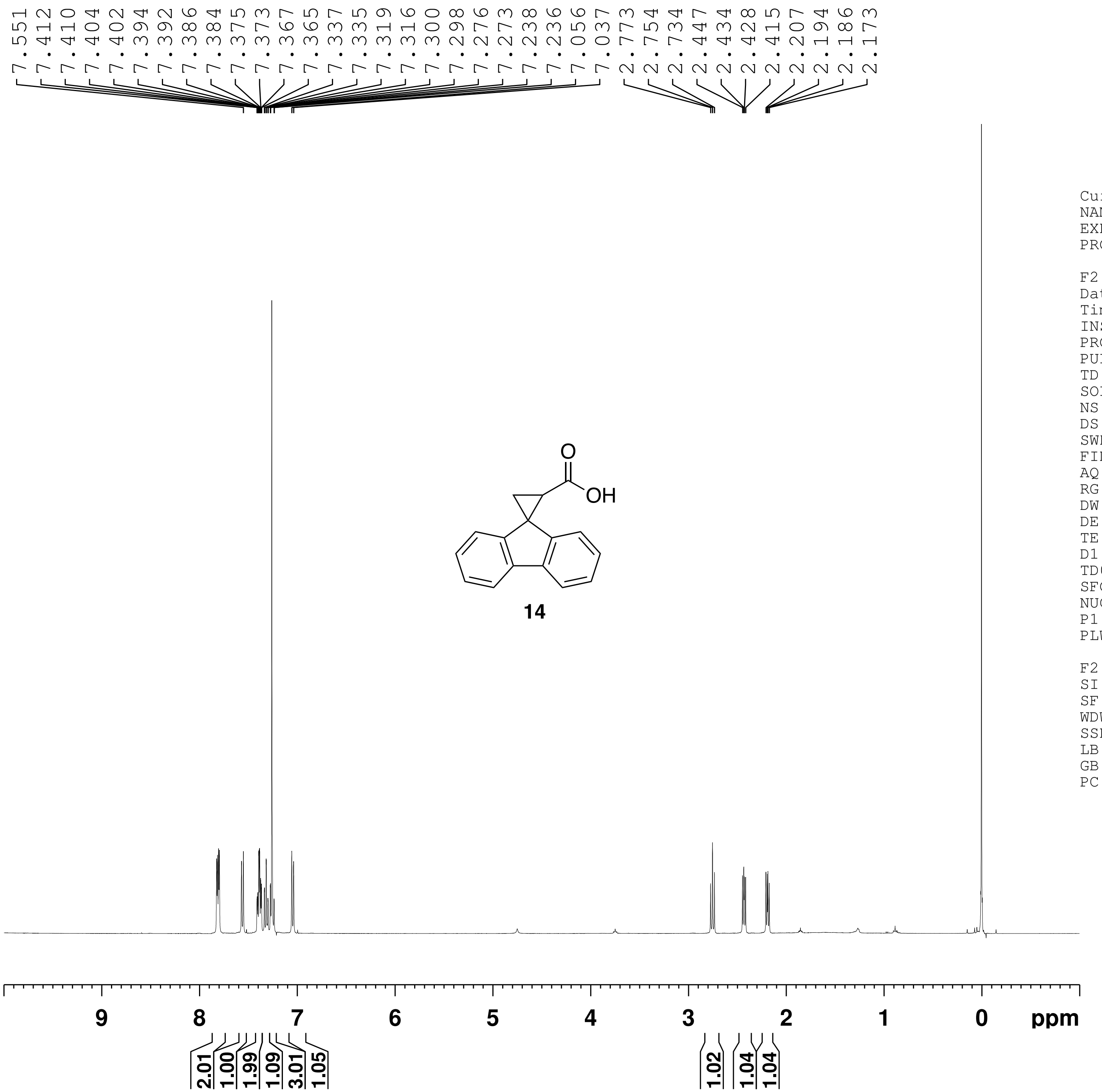


```
Current Data Parameters
NAME     CHW-VI-93_purePROTON
EXPNO                   1
PROCNO                  1

F2 - Acquisition Parameters
Date_           20220511
Time               10.18 h
INSTRUM            spect
PROBHD   Z150354_0001 (
PULPROG             zg30
TD                 65536
SOLVENT            CDCl3
NS                    16
DS                     2
SWH             8012.820 Hz
FIDRES          0.244532 Hz
AQ             4.0894465 sec
RG                 164.8
DW                62.400 usec
DE                 30.00 usec
TE                 298.0 K
D1            1.00000000 sec
TD0                    1
SFO1         400.3024719 MHz
NUC1                  1H
P1                 12.00 usec
PLW1          4.64209986 W

F2 - Processing parameters
SI                 65536
SF           400.3000094 MHz
WDW                   EM
SSB                    0
LB                  0.30 Hz
GB                     0
PC                  1.00
```

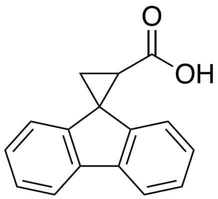

14

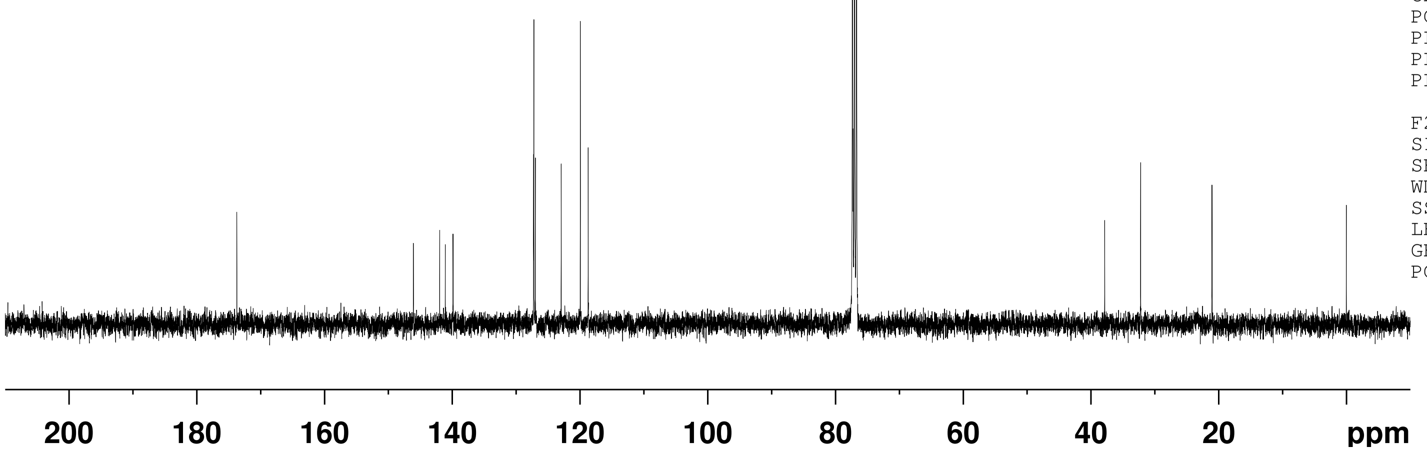


```
Current Data Parameters
NAME      CHW-VI-93_pureCARBON
EXPNO                    1
PROCNO                   1

F2 - Acquisition Parameters
Date_            20220511
Time                10.35 h
INSTRUM             spect
PROBHD    Z150354_0001 (
PULPROG           zgpg30
TD                 65356
SOLVENT            CDCl3
NS                   128
DS                     4
SWH            24038.461 Hz
FIDRES          0.735616 Hz
AQ             1.3594048 sec
RG                 25.77
DW                20.800 usec
DE                 25.00 usec
TE                 298.0 K
D1            2.00000000 sec
D11           0.03000000 sec
TD0                    1
SFO1         100.6655806 MHz
NUC1                 13C
P1                 10.00 usec
PLW1         17.99699974 W
SFO2         400.3016012 MHz
NUC2                  1H
CPDPRG[2         waltz16
PCPD2              80.00 usec
PLW2          4.64209986 W
PLW12         0.10445000 W
PLW13         0.05245300 W

F2 - Processing parameters
SI                 32768
SF           100.6555165 MHz
WDW                   EM
SSB                    0
LB                  1.00 Hz
GB                     0
PC                  1.40
```

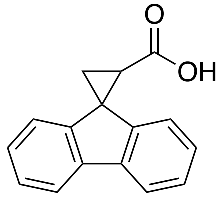

14

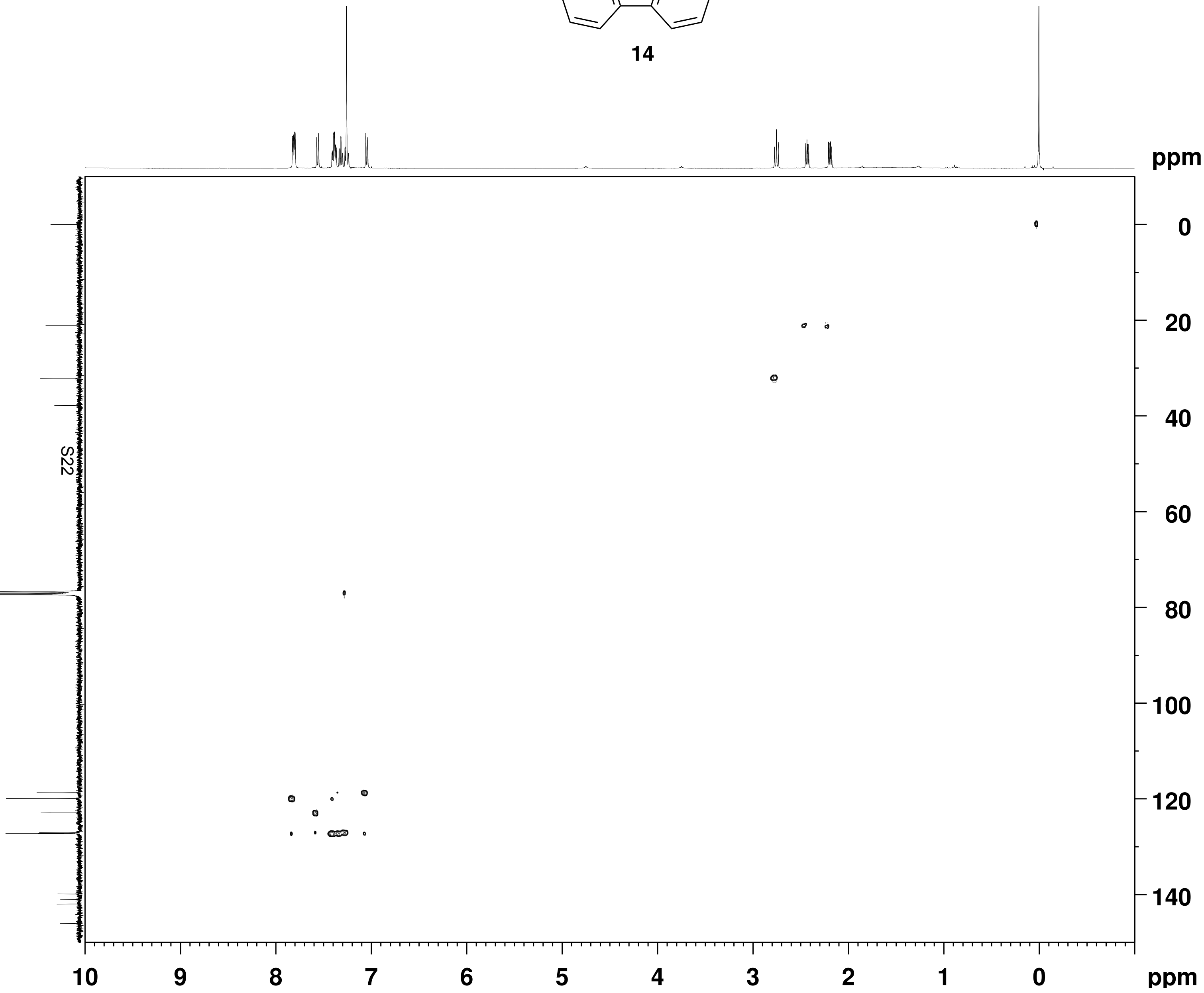


```
Current Data Parameters
NAME       CHW-VI-93_hsqc
EXPNO                   1
PROCNO                  1

F2 - Acquisition Parameters
Date_            20220511
Time                10.37 h
INSTRUM             spect
PROBHD    Z150354_0001 (
PULPROG  hsqcedetgpsisp2.3
TD                   2048
SOLVENT             CDCl3
NS                      2
DS                     32
SWH              5208.333 Hz
FIDRES           5.086263 Hz
AQ              0.1966080 sec
RG                 207.85
DW                 96.000 usec
DE                  30.00 usec
TE                  298.0 K
CNST2         145.0000000
CNST17         -0.5000000
D0             0.00000300 sec
D1             1.50000000 sec
D4             0.00172414 sec
D11            0.03000000 sec
D16            0.00020000 sec
D21            0.00344800 sec
D24            0.00086200 sec
IN0            0.00003010 sec
TDav                    1
SFO1          400.3018814 MHz
NUC1                   1H
P1                  12.00 usec
P2                  24.00 usec
P28                     0 usec
PLW1           4.64209986 W
SFO2          100.6630642 MHz
NUC2                  13C
CPDPRG[2            garp4
P3                  10.00 usec
P14                500.00 usec
P24               2000.00 usec
P31               2119.00 usec
PCPD2               65.00 usec
PLW0                    0 W
PLW2          17.99699974 W
PLW12          0.42594999 W
SPNAM[3] Crp60,0.5,20.1
SPOAL3              0.500
SPOFFS3                 0 Hz
SPW3           2.74970007 W
SPNAM[7]     Crp60comp.4
SPOAL7              0.500
SPOFFS7                 0 Hz
SPW7           2.74970007 W
SPNAM[18  Crp60_xfilt.2
SPOAL18             0.500
SPOFFS18                0 Hz
SPW18          0.52970999 W
GPNAM[1]      SMSQ10.100
GPZ1                80.00 %
GPNAM[2]      SMSQ10.100
GPZ2                20.10 %
GPNAM[3]      SMSQ10.100
GPZ3                11.00 %
GPNAM[4]      SMSQ10.100
GPZ4                -5.00 %
P16               1000.00 usec
P19                600.00 usec

F1 - Acquisition parameters
TD                    256
SFO1             100.6631 MHz
FIDRES         129.775742 Hz
SW                165.019 ppm
FnMODE     Echo-Antiecho

F2 - Processing parameters
SI                   1024
SF            400.3000000 MHz
WDW                 QSINE
SSB                     2
LB                      0 Hz
GB                      0
PC                   1.40

F1 - Processing parameters
SI                   1024
MC2          echo-antiecho
SF            100.6555151 MHz
WDW                 QSINE
SSB                     2
LB                      0 Hz
GB                      0
```

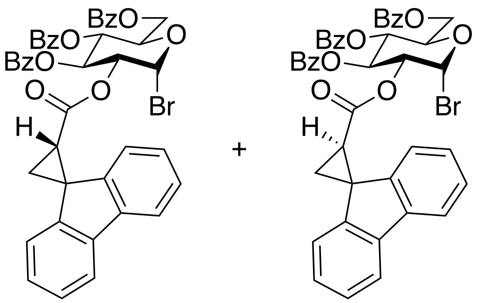

15a + **15b**

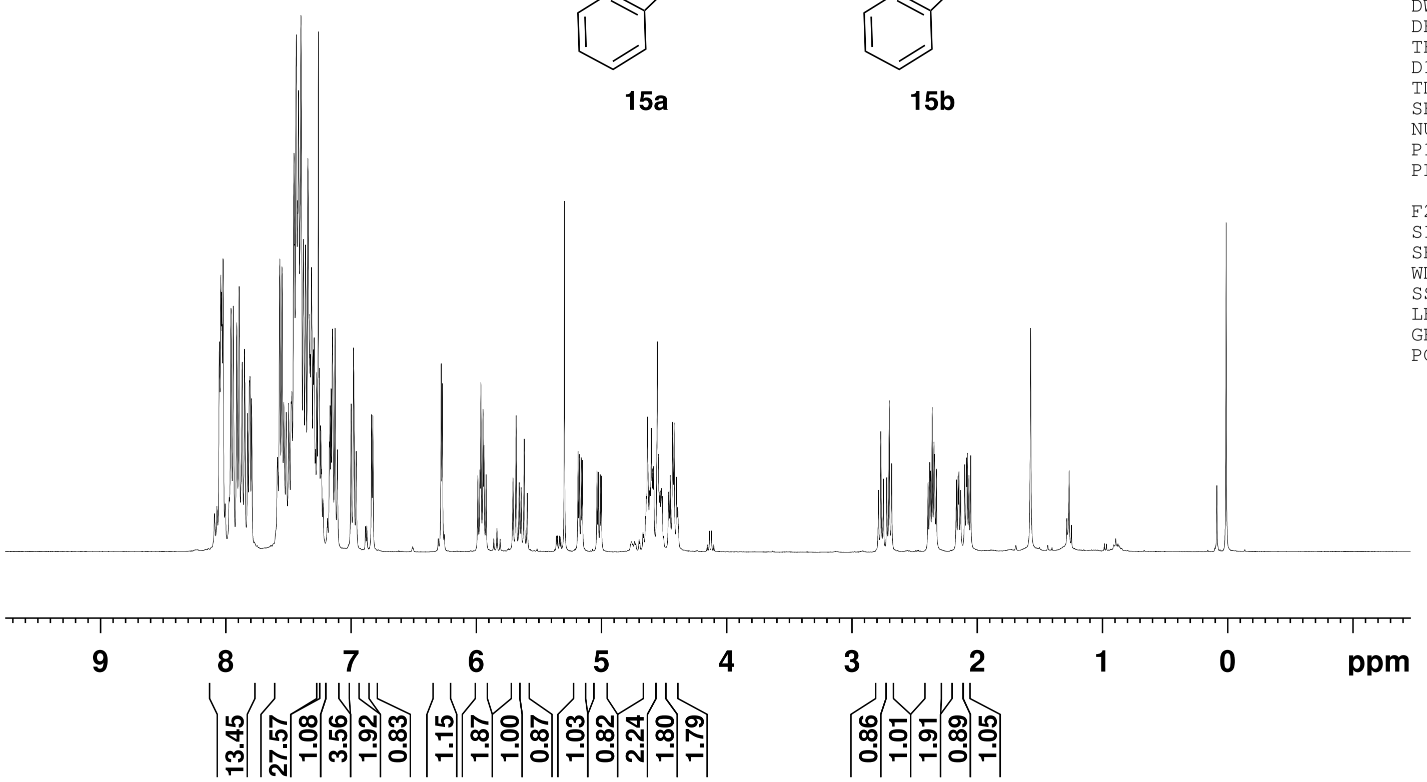


```
Current Data Parameters
NAME     CHW-VII-142_topPROTON
EXPNO                   1
PROCNO                  1

F2 - Acquisition Parameters
Date_          20221201
Time              16.15 h
INSTRUM           spect
PROBHD   Z150354_0001 (
PULPROG            zg30
TD                65536
SOLVENT           CDCl3
NS                   16
DS                    2
SWH           8012.820 Hz
FIDRES        0.244532 Hz
AQ           4.0894465 sec
RG               81.45
DW              62.400 usec
DE               30.00 usec
TE               298.0 K
D1          1.00000000 sec
TD0                   1
SFO1       400.3024719 MHz
NUC1                 1H
P1               12.00 usec
PLW1        4.64209986 W

F2 - Processing parameters
SI                65536
SF         400.3000093 MHz
WDW                  EM
SSB                   0
LB                 0.30 Hz
GB                    0
PC                 1.00
```

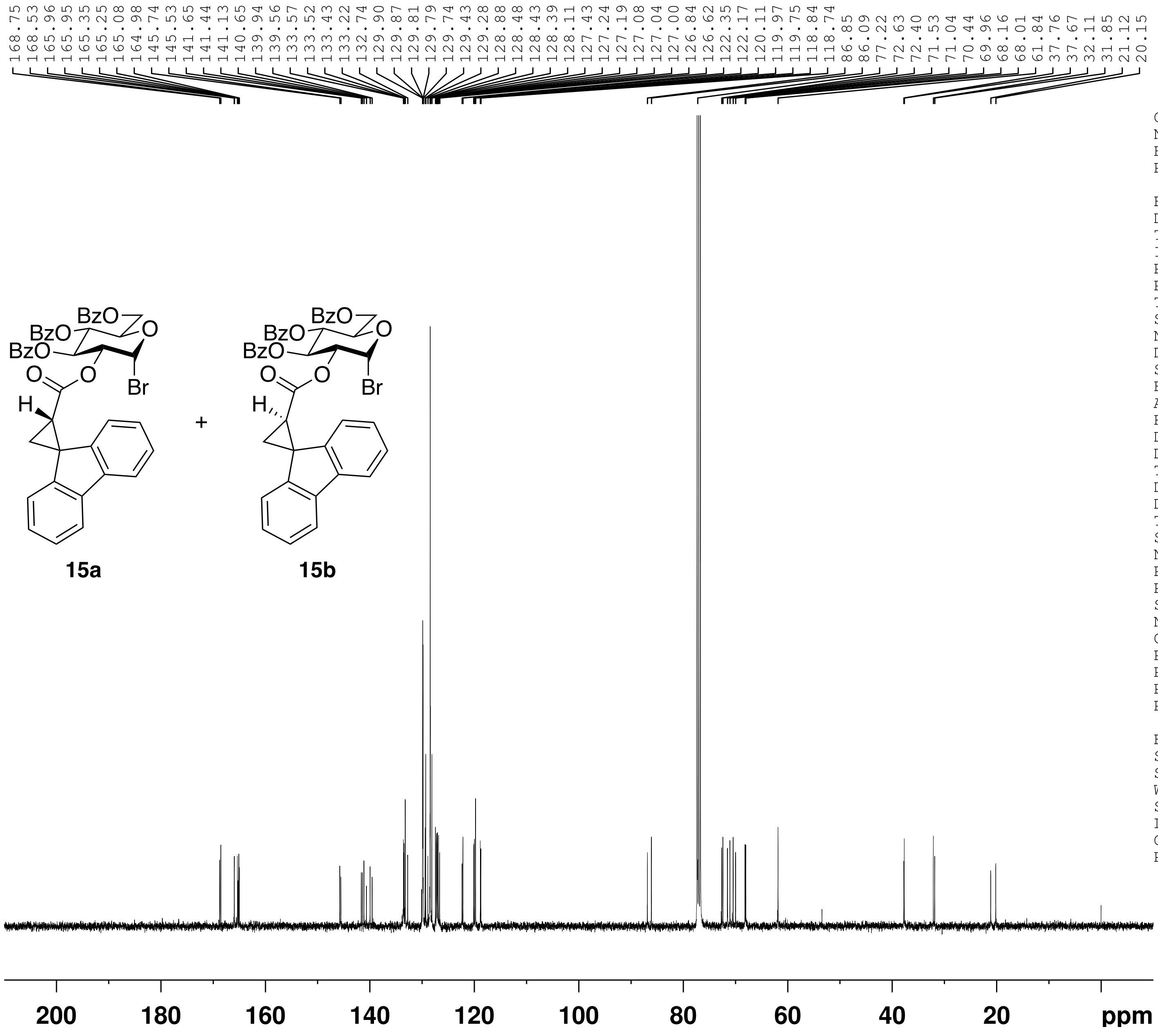

15a + **15b**


```
Current Data Parameters
NAME      CHW-VII-142_topCARBON
EXPNO                     1
PROCNO                    1

F2 - Acquisition Parameters
Date_           20221201
Time               16.31 h
INSTRUM            spect
PROBHD    Z150354_0001 (
PULPROG          zgpg30
TD                65356
SOLVENT           CDCl3
NS                  128
DS                    4
SWH           24038.461 Hz
FIDRES         0.735616 Hz
AQ            1.3594048 sec
RG                45.21
DW               20.800 usec
DE                25.00 usec
TE                298.0 K
D1           2.00000000 sec
D11          0.03000000 sec
TD0                   1
SFO1        100.6655806 MHz
NUC1                13C
P1                10.00 usec
PLW1        17.99699974 W
SFO2        400.3016012 MHz
NUC2                 1H
CPDPRG[2        waltz16
PCPD2             80.00 usec
PLW2         4.64209986 W
PLW12        0.10445000 W
PLW13        0.05245300 W

F2 - Processing parameters
SI                32768
SF          100.6555175 MHz
WDW                  EM
SSB                   0
LB                 1.00 Hz
GB                    0
PC                 1.40
```

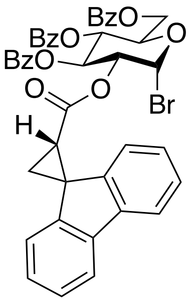

15a

+

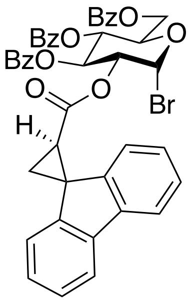

15b

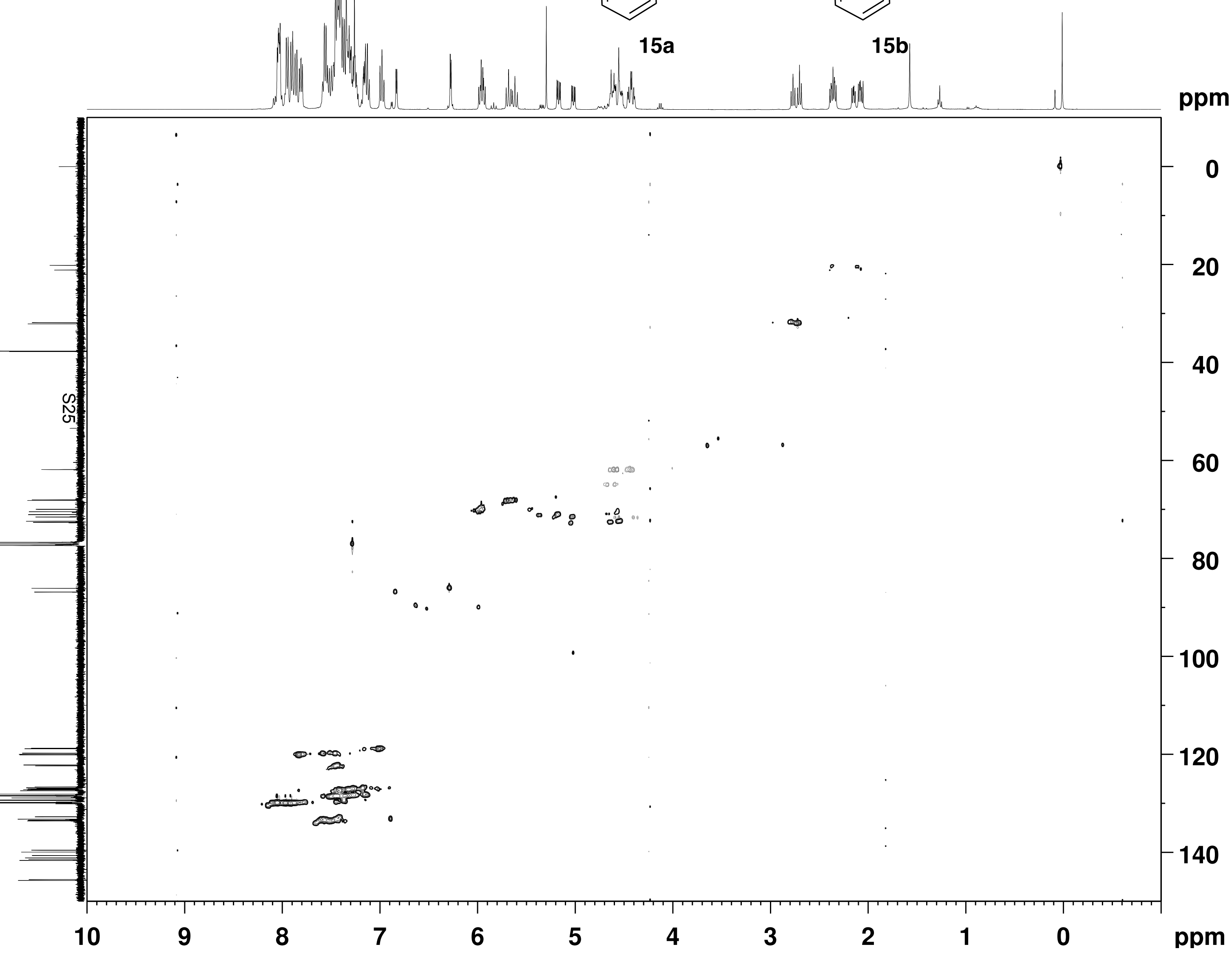


```
Current Data Parameters
NAME       CHW-VIII-157_hsqc
EXPNO                     1
PROCNO                    1

F2 - Acquisition Parameters
Date_          20230222
Time               9.45 h
INSTRUM           spect
PROBHD   Z150354_0001 (
PULPROG  hsqcedetgpsisp2.3
TD                 2048
SOLVENT           CDCl3
NS                    2
DS                   32
SWH            5208.333 Hz
FIDRES         5.086263 Hz
AQ            0.1966080 sec
RG               207.85
DW               96.000 usec
DE                30.00 usec
TE                298.0 K
CNST2       145.0000000
CNST17       -0.5000000
D0           0.00000300 sec
D1           1.50000000 sec
D4           0.00172414 sec
D11          0.03000000 sec
D16          0.00020000 sec
D21          0.00344800 sec
D24          0.00086200 sec
IN0          0.00003010 sec
TDav                  1
SFO1        400.3018814 MHz
NUC1                 1H
P1                12.00 usec
P2                24.00 usec
P28                   0 usec
PLW1         4.64209986 W
SFO2        100.6630642 MHz
NUC2                13C
CPDPRG[2          garp4
P3                10.00 usec
P14              500.00 usec
P24             2000.00 usec
P31             2119.00 usec
PCPD2             65.00 usec
PLW0                  0 W
PLW2        17.99699974 W
PLW12        0.42594999 W
SPNAM[3] Crp60,0.5,20.1
SPOAL3            0.500
SPOFFS3               0 Hz
SPW3         2.74970007 W
SPNAM[7]    Crp60comp.4
SPOAL7            0.500
SPOFFS7               0 Hz
SPW7         2.74970007 W
SPNAM[18  Crp60_xfilt.2
SPOAL18           0.500
SPOFFS18              0 Hz
SPW18        0.52970999 W
GPNAM[1]     SMSQ10.100
GPZ1              80.00 %
GPNAM[2]     SMSQ10.100
GPZ2              20.10 %
GPNAM[3]     SMSQ10.100
GPZ3              11.00 %
GPNAM[4]     SMSQ10.100
GPZ4              -5.00 %
P16             1000.00 usec
P19              600.00 usec

F1 - Acquisition parameters
TD                  256
SFO1           100.6631 MHz
FIDRES       129.775742 Hz
SW              165.019 ppm
FnMODE     Echo-Antiecho

F2 - Processing parameters
SI                 1024
SF          400.3000000 MHz
WDW               QSINE
SSB                   2
LB                    0 Hz
GB                    0
PC                 1.40

F1 - Processing parameters
SI                 1024
MC2        echo-antiecho
SF          100.6555151 MHz
WDW               QSINE
SSB                   2
LB                    0 Hz
GB                    0
```

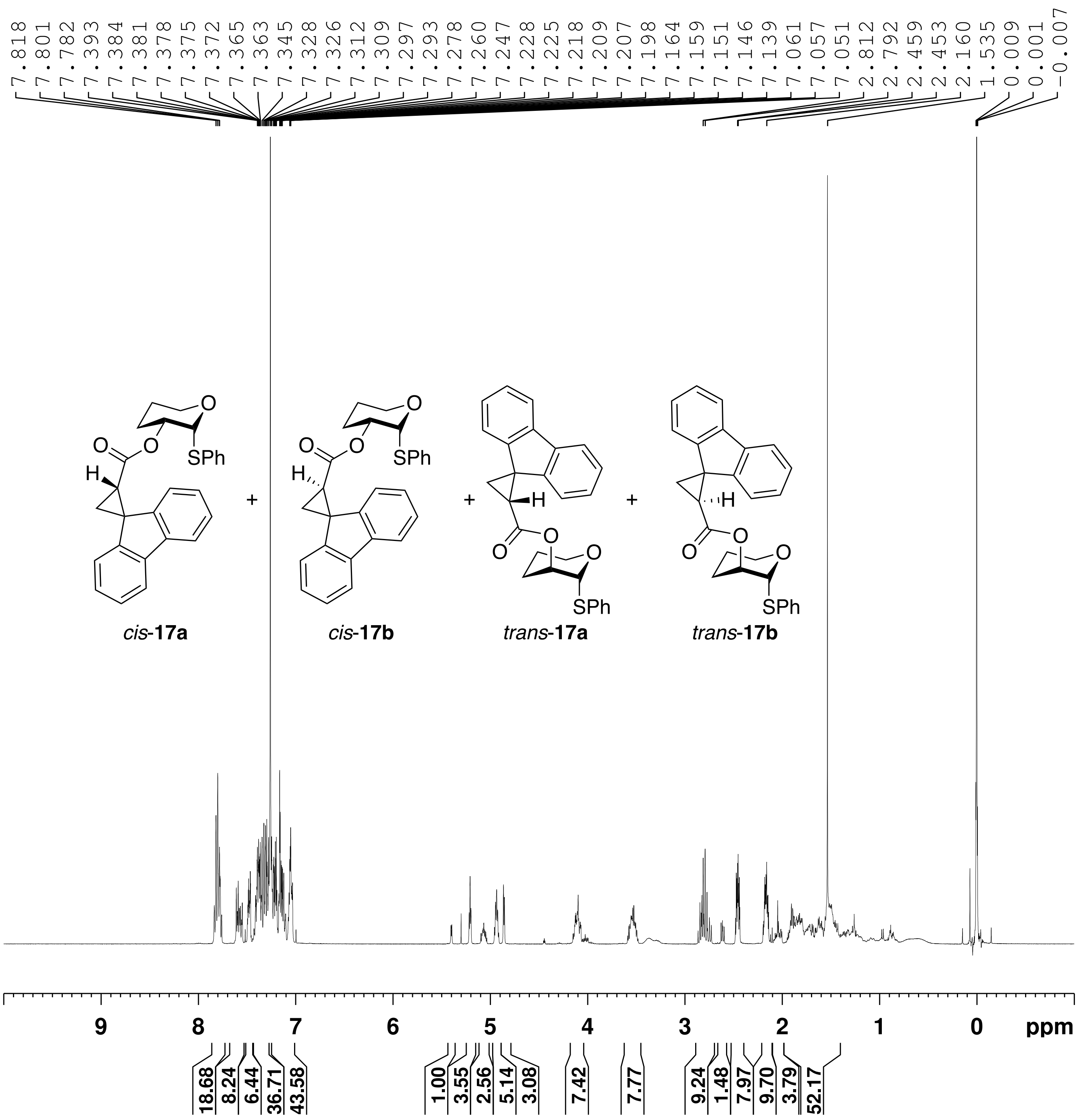


```
Current Data Parameters
NAME      CHW-VIII-5_purePROTON
EXPNO                     1
PROCNO                    1

F2 - Acquisition Parameters
Date_          20230213
Time              13.55 h
INSTRUM           spect
PROBHD    Z150354_0001 (
PULPROG            zg30
TD                65536
SOLVENT           CDCl3
NS                   16
DS                    2
SWH            8012.820 Hz
FIDRES         0.244532 Hz
AQ            4.0894465 sec
RG               207.85
DW               62.400 usec
DE                30.00 usec
TE                298.0 K
D1           1.00000000 sec
TD0                   1
SFO1        400.3024719 MHz
NUC1                 1H
P1                12.00 usec
PLW1         4.64209986 W

F2 - Processing parameters
SI                65536
SF          400.3000094 MHz
WDW                  EM
SSB                   0
LB                 0.30 Hz
GB                    0
PC                 1.00
```

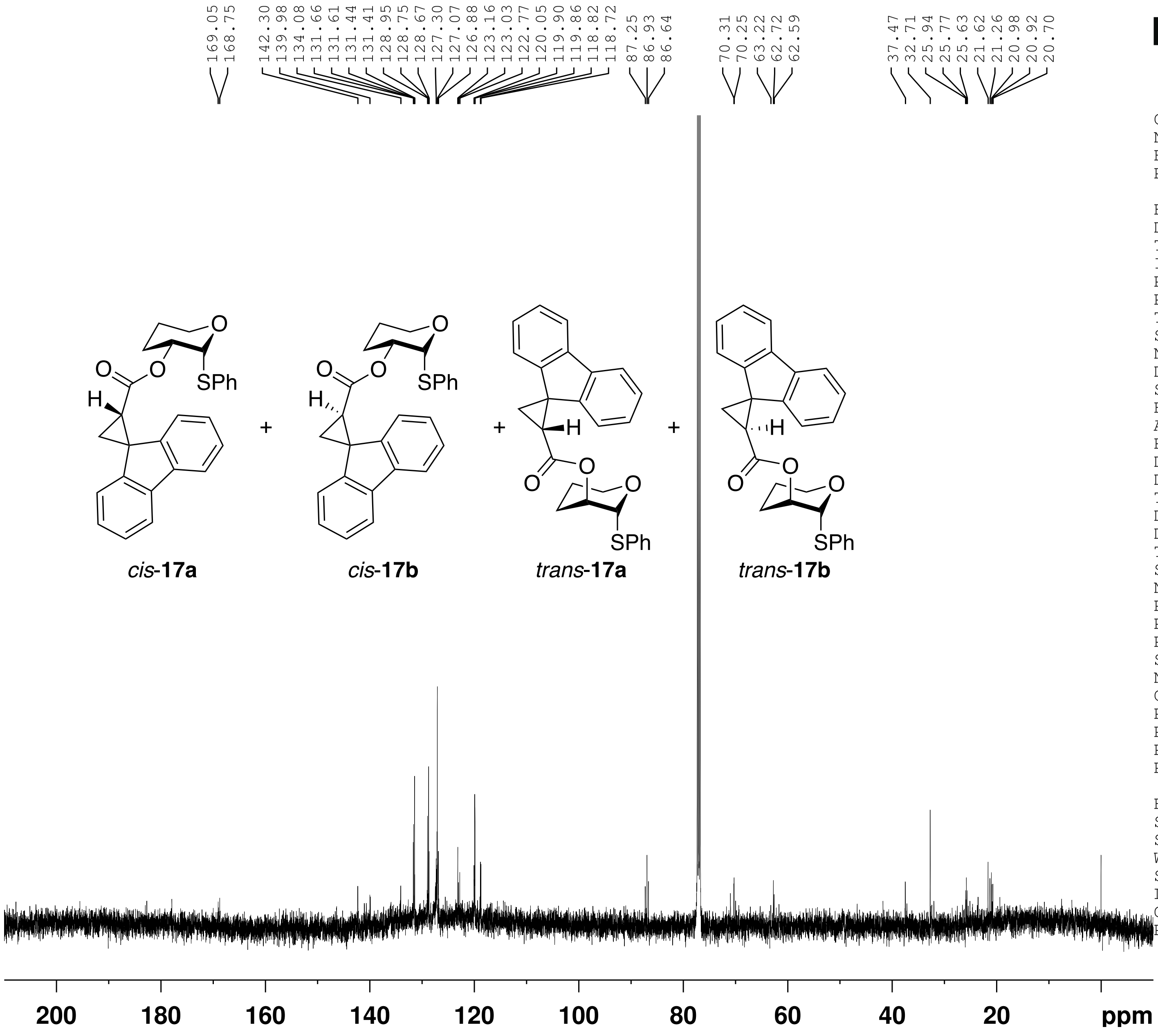

Current Data Parameters
NAME CHW-VIII-5_carbon2
EXPNO 12
PROCNO 1

F2 - Acquisition Parameters
Date_ 20230301
Time 7.41 h
INSTRUM Avance Neo 500
PROBHD Z167894_0001 (
PULPROG zgpg30
TD 65536
SOLVENT CDCl3
NS 128
DS 4
SWH 30120.482 Hz
FIDRES 0.919204 Hz
AQ 1.0878977 sec
RG 14.0086
DW 16.600 usec
DE 28.00 usec
TE 298.1 K
D1 2.00000000 sec
D11 0.03000000 sec
TD0 1
SFO1 125.7879676 MHz
NUC1 13C
P0 3.33 usec
P1 10.00 usec
PLW1 50.10900116 W
SFO2 500.2020008 MHz
NUC2 1H
CPDPRG[2 waltz65
PCPD2 80.00 usec
PLW2 12.03499985 W
PLW12 0.23987190 W
PLW13 0.12022380 W

F2 - Processing parameters
SI 32768
SF 125.7753900 MHz
WDW EM
SSB 0
LB 1.00 Hz
GB 0
PC 1.40

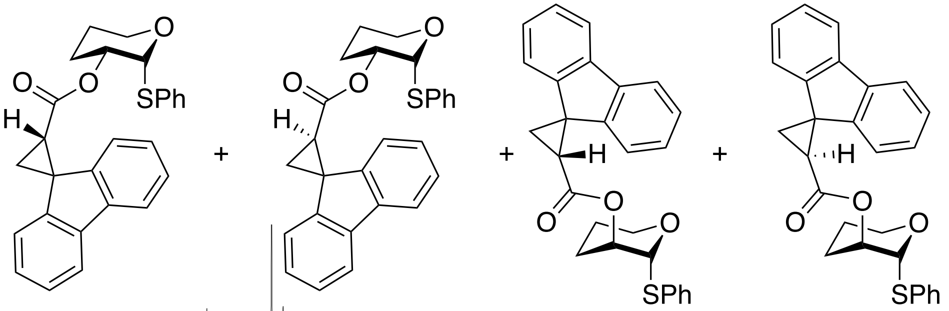

cis-**17a** + *cis*-**17b** + *trans*-**17a** + *trans*-**17b**

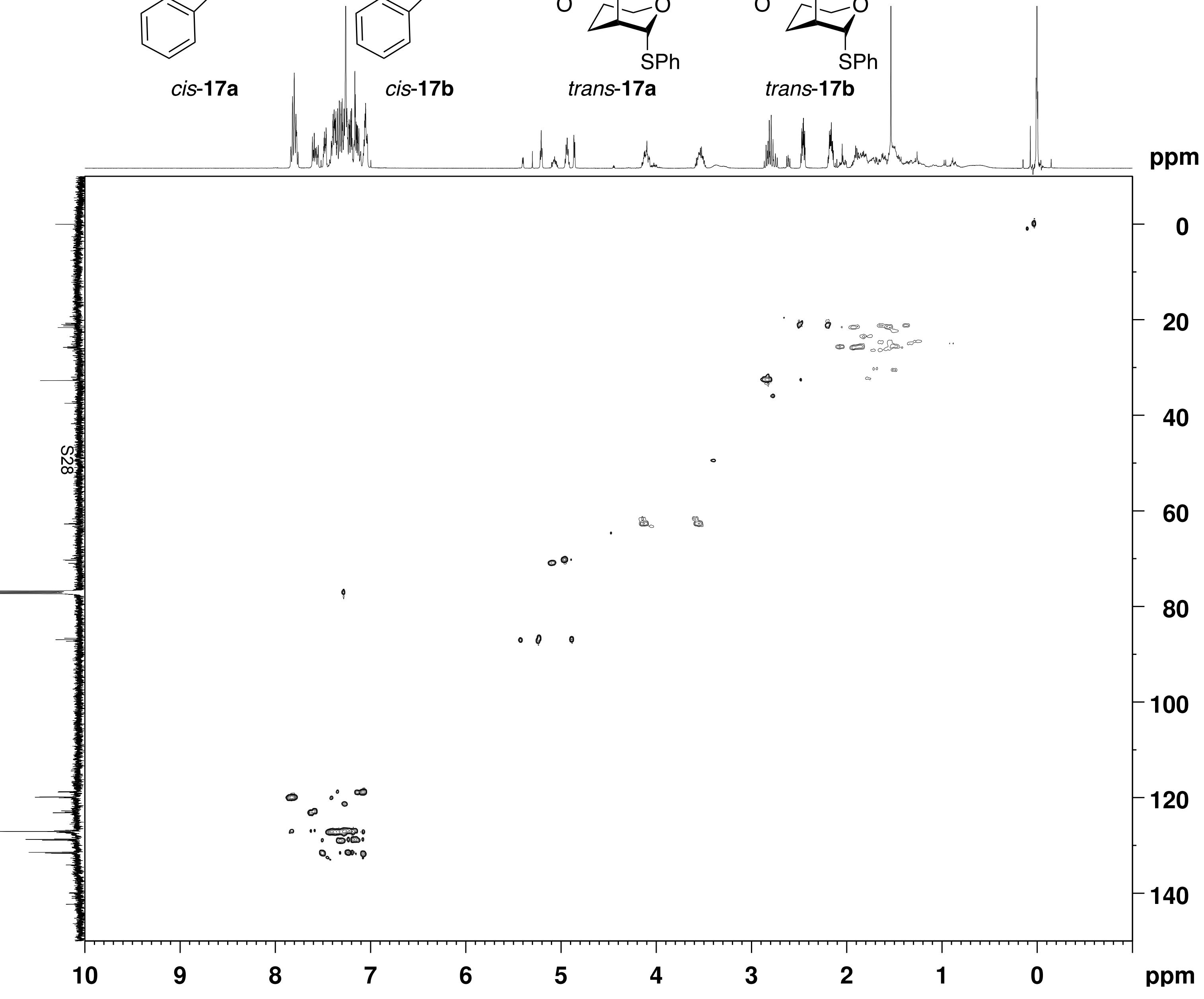


```
Current Data Parameters
NAME     CHW-VIII-5_hsqc
EXPNO              1
PROCNO             1

F2 - Acquisition Parameters
Date_          20230223
Time               7.53 h
INSTRUM           spect
PROBHD   Z150354_0001 (
PULPROG  hsqcedetgpsisp2.3
TD                 2048
SOLVENT           CDCl3
NS                    2
DS                   32
SWH            5208.333 Hz
FIDRES         5.086263 Hz
AQ            0.1966080 sec
RG               207.85
DW               96.000 usec
DE                30.00 usec
TE                298.0 K
CNST2       145.0000000
CNST17       -0.5000000
D0           0.00000300 sec
D1           1.50000000 sec
D4           0.00172414 sec
D11          0.03000000 sec
D16          0.00020000 sec
D21          0.00344800 sec
D24          0.00086200 sec
IN0          0.00003010 sec
TDav                  1
SFO1        400.3018814 MHz
NUC1                 1H
P1                12.00 usec
P2                24.00 usec
P28                   0 usec
PLW1         4.64209986 W
SFO2        100.6630642 MHz
NUC2                13C
CPDPRG[2          garp4
P3                10.00 usec
P14              500.00 usec
P24             2000.00 usec
P31             2119.00 usec
PCPD2             65.00 usec
PLW0                  0 W
PLW2        17.99699974 W
PLW12        0.42594999 W
SPNAM[3] Crp60,0.5,20.1
SPOAL3            0.500
SPOFFS3               0 Hz
SPW3         2.74970007 W
SPNAM[7]    Crp60comp.4
SPOAL7            0.500
SPOFFS7               0 Hz
SPW7         2.74970007 W
SPNAM[18  Crp60_xfilt.2
SPOAL18           0.500
SPOFFS18              0 Hz
SPW18        0.52970999 W
GPNAM[1]     SMSQ10.100
GPZ1              80.00 %
GPNAM[2]     SMSQ10.100
GPZ2              20.10 %
GPNAM[3]     SMSQ10.100
GPZ3              11.00 %
GPNAM[4]     SMSQ10.100
GPZ4              -5.00 %
P16             1000.00 usec
P19              600.00 usec

F1 - Acquisition parameters
TD                  256
SFO1           100.6631 MHz
FIDRES       129.775742 Hz
SW              165.019 ppm
FnMODE     Echo-Antiecho

F2 - Processing parameters
SI                 1024
SF          400.3000000 MHz
WDW               QSINE
SSB                   2
LB                    0 Hz
GB                    0
PC                 1.40

F1 - Processing parameters
SI                 1024
MC2        echo-antiecho
SF          100.6555151 MHz
WDW               QSINE
SSB                   2
LB                    0 Hz
GB                    0
```

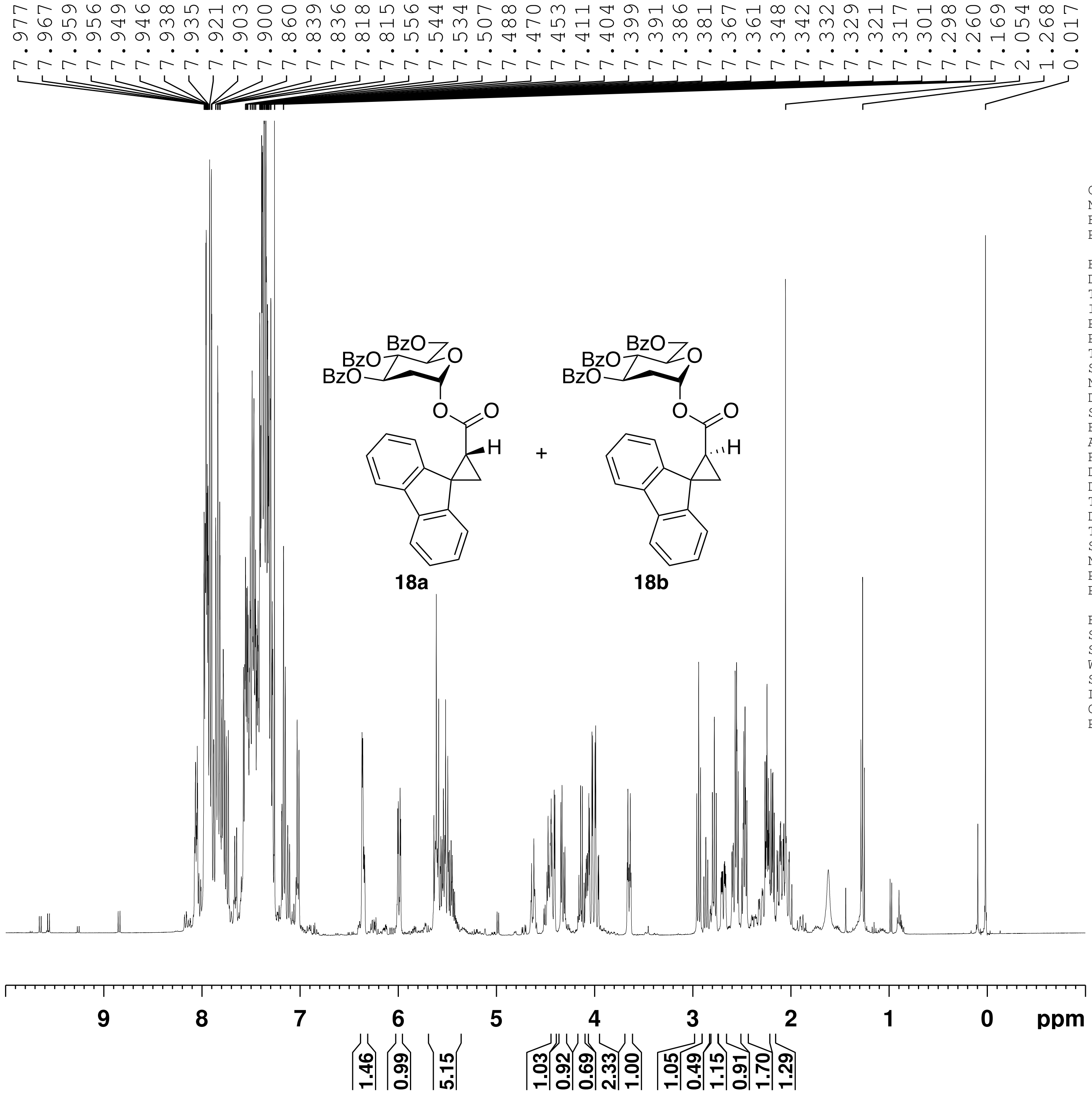


```
Current Data Parameters
NAME     CHW-VII-173_purePROTON2
EXPNO                 1
PROCNO                1

F2 - Acquisition Parameters
Date_          20230130
Time               8.29 h
INSTRUM           spect
PROBHD   Z150354_0001 (
PULPROG            zg30
TD                65536
SOLVENT           CDCl3
NS                   16
DS                    2
SWH            8012.820 Hz
FIDRES         0.244532 Hz
AQ            4.0894465 sec
RG                58.09
DW               62.400 usec
DE                30.00 usec
TE                298.0 K
D1           1.00000000 sec
TD0                   1
SFO1        400.3024719 MHz
NUC1                 1H
P1                12.00 usec
PLW1         4.64209986 W

F2 - Processing parameters
SI                65536
SF          400.3000092 MHz
WDW                  EM
SSB                   0
LB                 0.30 Hz
GB                    0
PC                 1.00
```

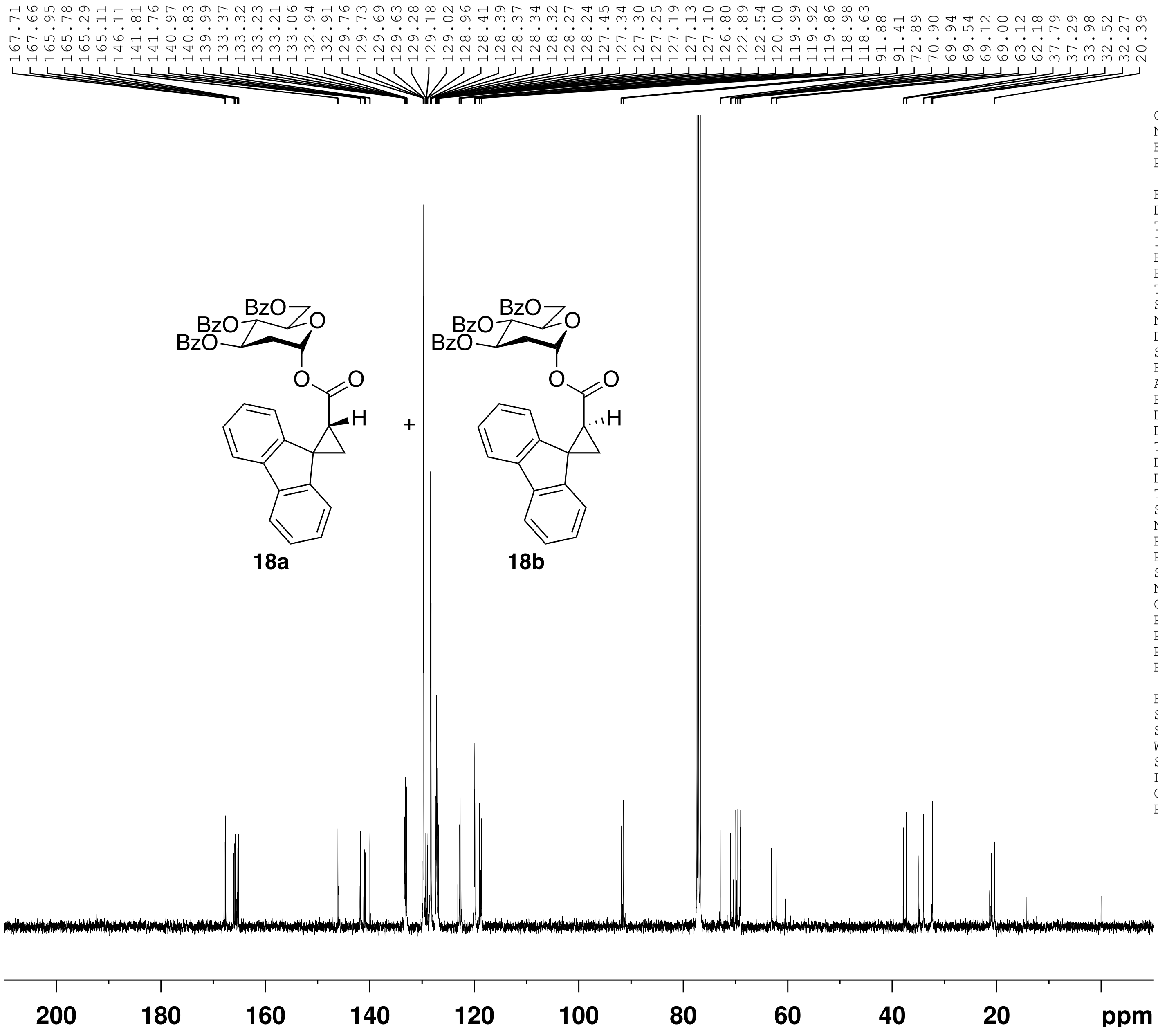


```
Current Data Parameters
NAME     CHW-VII-173_pureCARBON2
EXPNO                 1
PROCNO                1

F2 - Acquisition Parameters
Date_          20230130
Time               8.38 h
INSTRUM           spect
PROBHD   Z150354_0001 (
PULPROG         zgpg30
TD               65356
SOLVENT           CDCl3
NS                 128
DS                   4
SWH          24038.461 Hz
FIDRES        0.735616 Hz
AQ           1.3594048 sec
RG               29.21
DW              20.800 usec
DE               25.00 usec
TE               298.0 K
D1          2.00000000 sec
D11         0.03000000 sec
TD0                  1
SFO1       100.6655806 MHz
NUC1               13C
P1               10.00 usec
PLW1       17.99699974 W
SFO2       400.3016012 MHz
NUC2                1H
CPDPRG[2        waltz16
PCPD2            80.00 usec
PLW2        4.64209986 W
PLW12       0.10445000 W
PLW13       0.05245300 W

F2 - Processing parameters
SI               32768
SF         100.6555190 MHz
WDW                 EM
SSB                  0
LB                1.00 Hz
GB                   0
PC                1.40
```

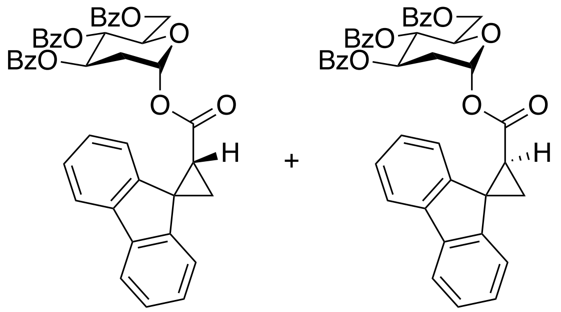

18a **18b**

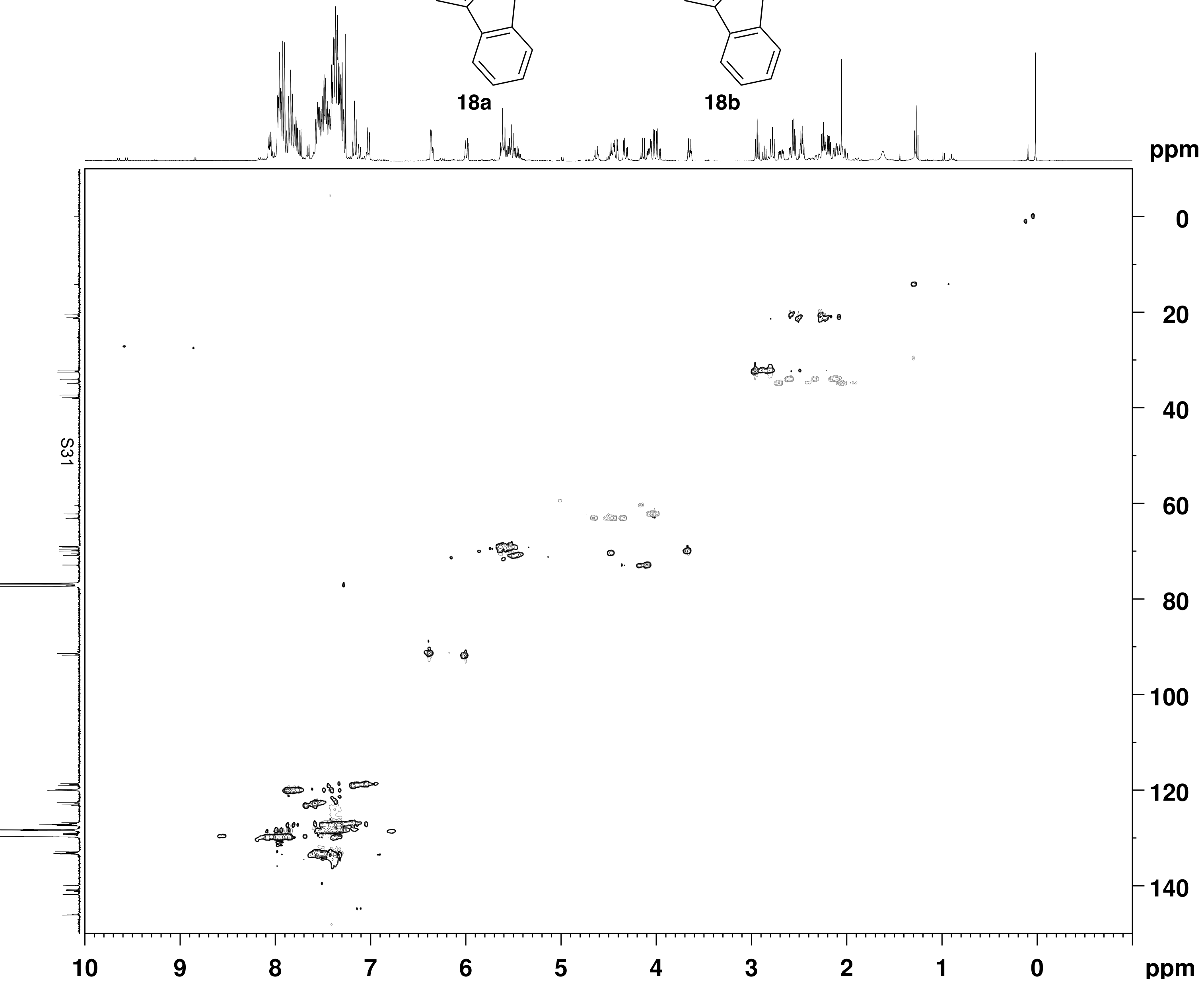


```
Current Data Parameters
NAME      CHW-VII-173_pureHSQC2
EXPNO                 1
PROCNO                1

F2 - Acquisition Parameters
Date_           20230130
Time                8.40 h
INSTRUM            spect
PROBHD   Z150354_0001 (
PULPROG  hsqcedetgpsisp2.3
TD                  2048
SOLVENT            CDCl3
NS                     2
DS                    32
SWH             5208.333 Hz
FIDRES          5.086263 Hz
AQ             0.1966080 sec
RG                207.85
DW                96.000 usec
DE                 30.00 usec
TE                 298.0 K
CNST2        145.0000000
CNST17        -0.5000000
D0            0.00000300 sec
D1            1.50000000 sec
D4            0.00172414 sec
D11           0.03000000 sec
D16           0.00020000 sec
D21           0.00344800 sec
D24           0.00086200 sec
IN0           0.00003010 sec
TDav                   1
SFO1         400.3018814 MHz
NUC1                  1H
P1                 12.00 usec
P2                 24.00 usec
P28                    0 usec
PLW1          4.64209986 W
SFO2         100.6630642 MHz
NUC2                 13C
CPDPRG[2            garp4
P3                 10.00 usec
P14               500.00 usec
P24              2000.00 usec
P31              2119.00 usec
PCPD2              65.00 usec
PLW0                   0 W
PLW2         17.99699974 W
PLW12         0.42594999 W
SPNAM[3] Crp60,0.5,20.1
SPOAL3             0.500
SPOFFS3                0 Hz
SPW3          2.74970007 W
SPNAM[7]     Crp60comp.4
SPOAL7             0.500
SPOFFS7                0 Hz
SPW7          2.74970007 W
SPNAM[18  Crp60_xfilt.2
SPOAL18            0.500
SPOFFS18               0 Hz
SPW18         0.52970999 W
GPNAM[1]      SMSQ10.100
GPZ1               80.00 %
GPNAM[2]      SMSQ10.100
GPZ2               20.10 %
GPNAM[3]      SMSQ10.100
GPZ3               11.00 %
GPNAM[4]      SMSQ10.100
GPZ4               -5.00 %
P16              1000.00 usec
P19               600.00 usec

F1 - Acquisition parameters
TD                   256
SFO1            100.6631 MHz
FIDRES        129.775742 Hz
SW               165.019 ppm
FnMODE      Echo-Antiecho

F2 - Processing parameters
SI                  1024
SF           400.3000000 MHz
WDW                QSINE
SSB                    2
LB                     0 Hz
GB                     0
PC                  1.40

F1 - Processing parameters
SI                  1024
MC2         echo-antiecho
SF           100.6555151 MHz
WDW                QSINE
SSB                    2
LB                     0 Hz
GB                     0
```

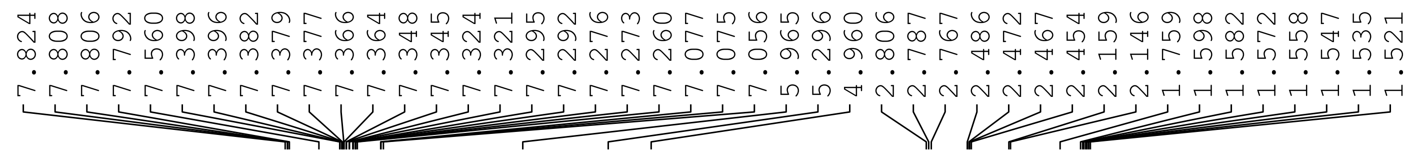

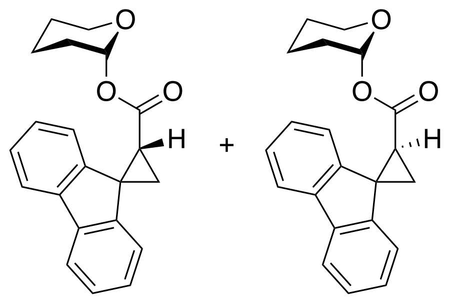

20a + **20b**

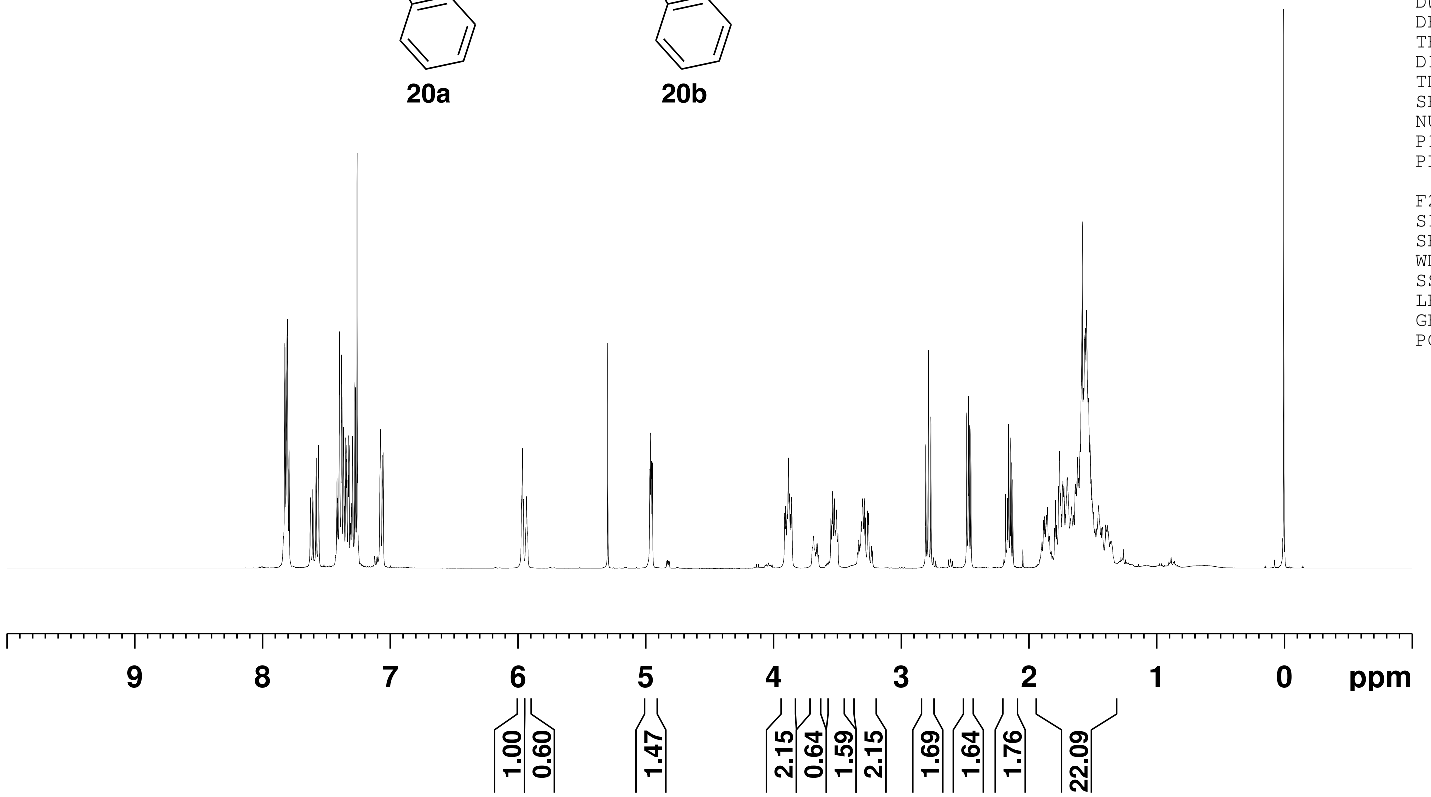


```
Current Data Parameters
NAME     CHW-VIII-13_purePROTON
EXPNO                 1
PROCNO                1

F2 - Acquisition Parameters
Date_          20230217
Time              14.08 h
INSTRUM           spect
PROBHD   Z150354_0001 (
PULPROG            zg30
TD                65536
SOLVENT           CDCl3
NS                   16
DS                    2
SWH            8012.820 Hz
FIDRES         0.244532 Hz
AQ            4.0894465 sec
RG                81.45
DW               62.400 usec
DE                30.00 usec
TE                298.0 K
D1           1.00000000 sec
TD0                   1
SFO1        400.3024719 MHz
NUC1                 1H
P1                12.00 usec
PLW1         4.64209986 W

F2 - Processing parameters
SI                65536
SF          400.3000095 MHz
WDW                  EM
SSB                   0
LB                 0.30 Hz
GB                    0
PC                 1.00
```

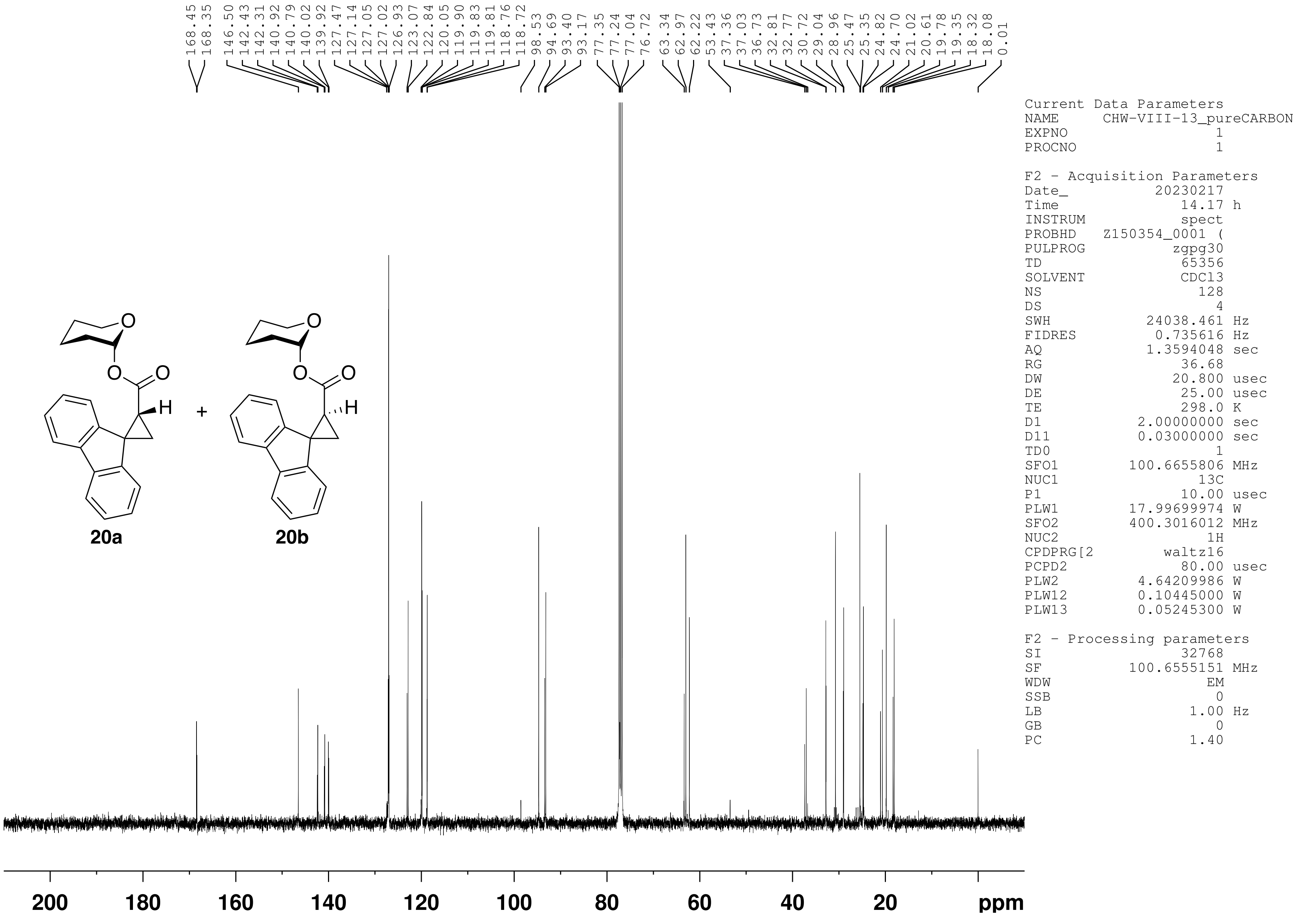
20a
+
20b
168.45
168.35
146.50
142.43
142.31
140.92
140.79
140.02
139.92
127.47
127.14
127.05
127.02
126.93
123.07
122.84
120.05
119.90
119.83
119.81
118.76
118.72
98.53
94.69
93.40
93.17
77.35
77.24
77.04
76.72
63.34
62.97
62.22
53.43
37.36
37.03
36.73
32.81
32.77
30.72
29.04
28.96
25.47
25.35
24.82
24.70
21.02
20.61
19.78
19.35
18.32
18.08
0.01
200
180
160
140
120
100
80
60
40
20
ppm
Current Data Parameters
NAME CHW-VIII-13_pureCARBON
EXPNO 1
PROCNO 1
F2 - Acquisition Parameters
Date_ 20230217
Time 14.17 h
INSTRUM spect
PROBHD Z150354_0001 (
PULPROG zgpg30
TD 65356
SOLVENT CDCl3
NS 128
DS 4
SWH 24038.461 Hz
FIDRES 0.735616 Hz
AQ 1.3594048 sec
RG 36.68
DW 20.800 usec
DE 25.00 usec
TE 298.0 K
D1 2.00000000 sec
D11 0.03000000 sec
TD0 1
SFO1 100.6655806 MHz
NUC1 13C
P1 10.00 usec
PLW1 17.99699974 W
SFO2 400.3016012 MHz
NUC2 1H
CPDPRG[2 waltz16
PCPD2 80.00 usec
PLW2 4.64209986 W
PLW12 0.10445000 W
PLW13 0.05245300 W
F2 - Processing parameters
SI 32768
SF 100.6555151 MHz
WDW EM
SSB 0
LB 1.00 Hz
GB 0
PC 1.40

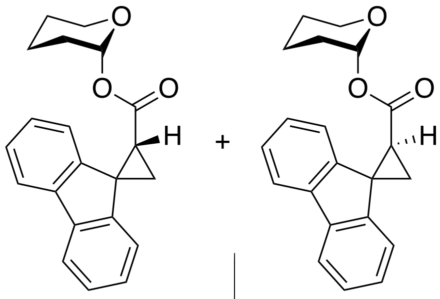

20a + **20b**

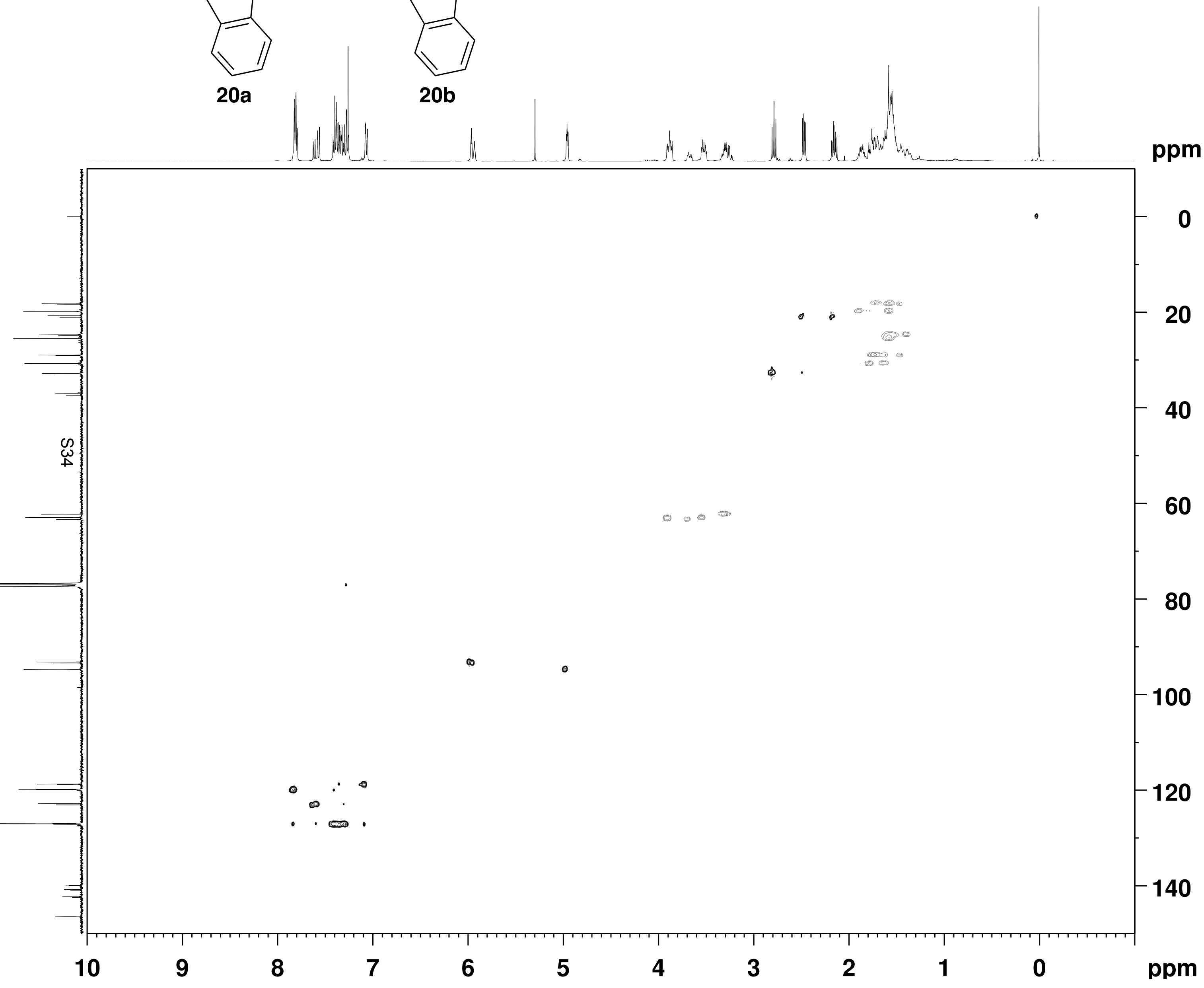


```
Current Data Parameters
NAME      CHW-VIII-13_hsqc
EXPNO                1
PROCNO               1

F2 - Acquisition Parameters
Date_          20230217
Time              14.19 h
INSTRUM           spect
PROBHD   Z150354_0001 (
PULPROG  hsqcedetgpsisp2.3
TD                 2048
SOLVENT           CDCl3
NS                    2
DS                   32
SWH            5208.333 Hz
FIDRES         5.086263 Hz
AQ            0.1966080 sec
RG               207.85
DW               96.000 usec
DE                30.00 usec
TE                298.0 K
CNST2       145.0000000
CNST17       -0.5000000
D0           0.00000300 sec
D1           1.50000000 sec
D4           0.00172414 sec
D11          0.03000000 sec
D16          0.00020000 sec
D21          0.00344800 sec
D24          0.00086200 sec
IN0          0.00003010 sec
TDav                  1
SFO1        400.3018814 MHz
NUC1                 1H
P1                12.00 usec
P2                24.00 usec
P28                   0 usec
PLW1         4.64209986 W
SFO2        100.6630642 MHz
NUC2                13C
CPDPRG[2          garp4
P3                10.00 usec
P14              500.00 usec
P24             2000.00 usec
P31             2119.00 usec
PCPD2             65.00 usec
PLW0                  0 W
PLW2        17.99699974 W
PLW12        0.42594999 W
SPNAM[3] Crp60,0.5,20.1
SPOAL3            0.500
SPOFFS3               0 Hz
SPW3         2.74970007 W
SPNAM[7]    Crp60comp.4
SPOAL7            0.500
SPOFFS7               0 Hz
SPW7         2.74970007 W
SPNAM[18  Crp60_xfilt.2
SPOAL18           0.500
SPOFFS18              0 Hz
SPW18        0.52970999 W
GPNAM[1]     SMSQ10.100
GPZ1              80.00 %
GPNAM[2]     SMSQ10.100
GPZ2              20.10 %
GPNAM[3]     SMSQ10.100
GPZ3              11.00 %
GPNAM[4]     SMSQ10.100
GPZ4              -5.00 %
P16             1000.00 usec
P19              600.00 usec

F1 - Acquisition parameters
TD                  256
SFO1           100.6631 MHz
FIDRES       129.775742 Hz
SW              165.019 ppm
FnMODE     Echo-Antiecho

F2 - Processing parameters
SI                 1024
SF          400.3000000 MHz
WDW               QSINE
SSB                   2
LB                    0 Hz
GB                    0
PC                 1.40

F1 - Processing parameters
SI                 1024
MC2        echo-antiecho
SF          100.6555151 MHz
WDW               QSINE
SSB                   2
LB                    0 Hz
GB                    0
```

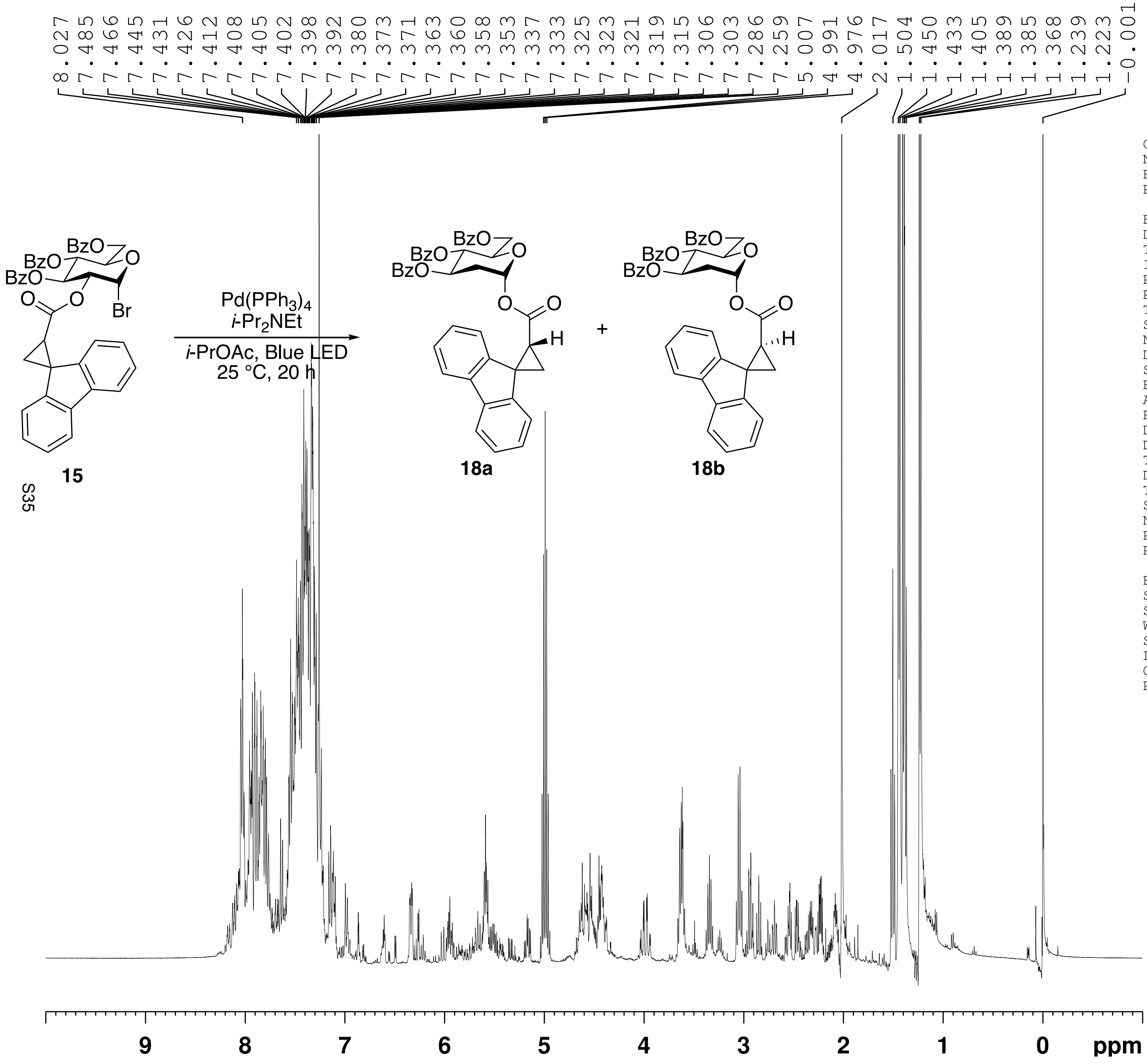

15 **18a** **18b**

```
Current Data Parameters
NAME      CHW-VII-161_crudePROTON
EXPNO                  1
PROCNO                 1

F2 - Acquisition Parameters
Date_            20221213
Time                9.10 h
INSTRUM            spect
PROBHD   Z150354_0001 (
PULPROG             zg30
TD                 65536
SOLVENT            CDCl3
NS                    16
DS                     2
SWH             8012.820 Hz
FIDRES          0.244532 Hz
AQ             4.0894465 sec
RG                117.47
DW                62.400 usec
DE                 30.00 usec
TE                 298.0 K
D1           1.00000000 sec
TD0                    1
SFO1         400.3024719 MHz
NUC1                  1H
P1                 12.00 usec
PLW1          4.64209986 W

F2 - Processing parameters
SI                 65536
SF           400.3000100 MHz
WDW                   EM
SSB                    0
LB                  0.30 Hz
GB                     0
PC                  1.00
```

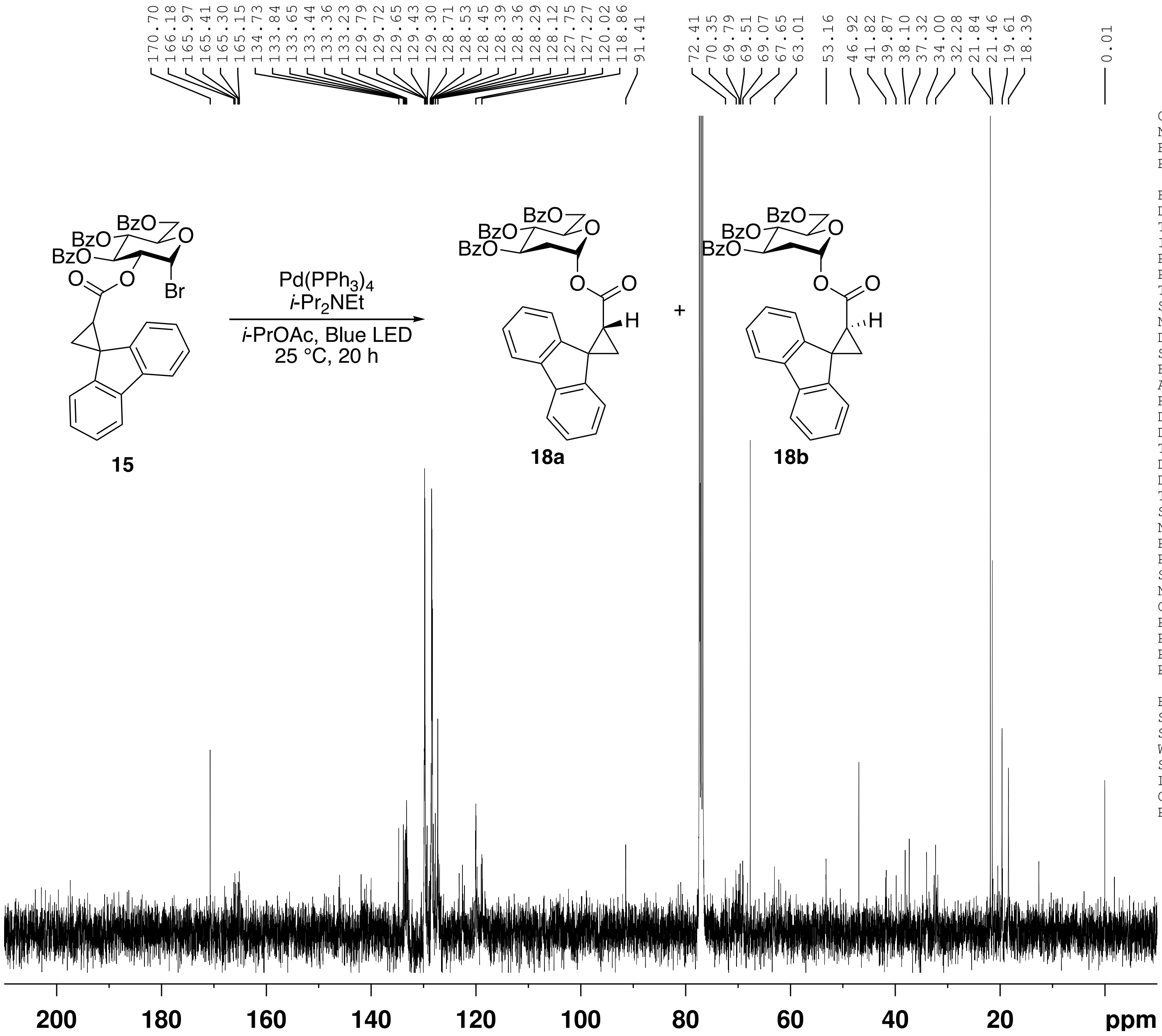


```
Current Data Parameters
NAME      CHW-VII-161_crudeCARBON
EXPNO                    1
PROCNO                   1

F2 - Acquisition Parameters
Date_             20221213
Time                  9.19 h
INSTRUM              spect
PROBHD   Z150354_0001 (
PULPROG             zgpg30
TD                   65356
SOLVENT              CDCl3
NS                     128
DS                       4
SWH            24038.461 Hz
FIDRES          0.735616 Hz
AQ             1.3594048 sec
RG                   29.21
DW                  20.800 usec
DE                   25.00 usec
TE                   298.0 K
D1            2.00000000 sec
D11           0.03000000 sec
TD0                      1
SFO1        100.6655806 MHz
NUC1                   13C
P1                   10.00 usec
PLW1         17.99699974 W
SFO2        400.3016012 MHz
NUC2                    1H
CPDPRG[2          waltz16
PCPD2                80.00 usec
PLW2          4.64209986 W
PLW12         0.10445000 W
PLW13         0.05245300 W

F2 - Processing parameters
SI                   32768
SF          100.6555151 MHz
WDW                     EM
SSB                      0
LB                    1.00 Hz
GB                       0
PC                    1.40
```

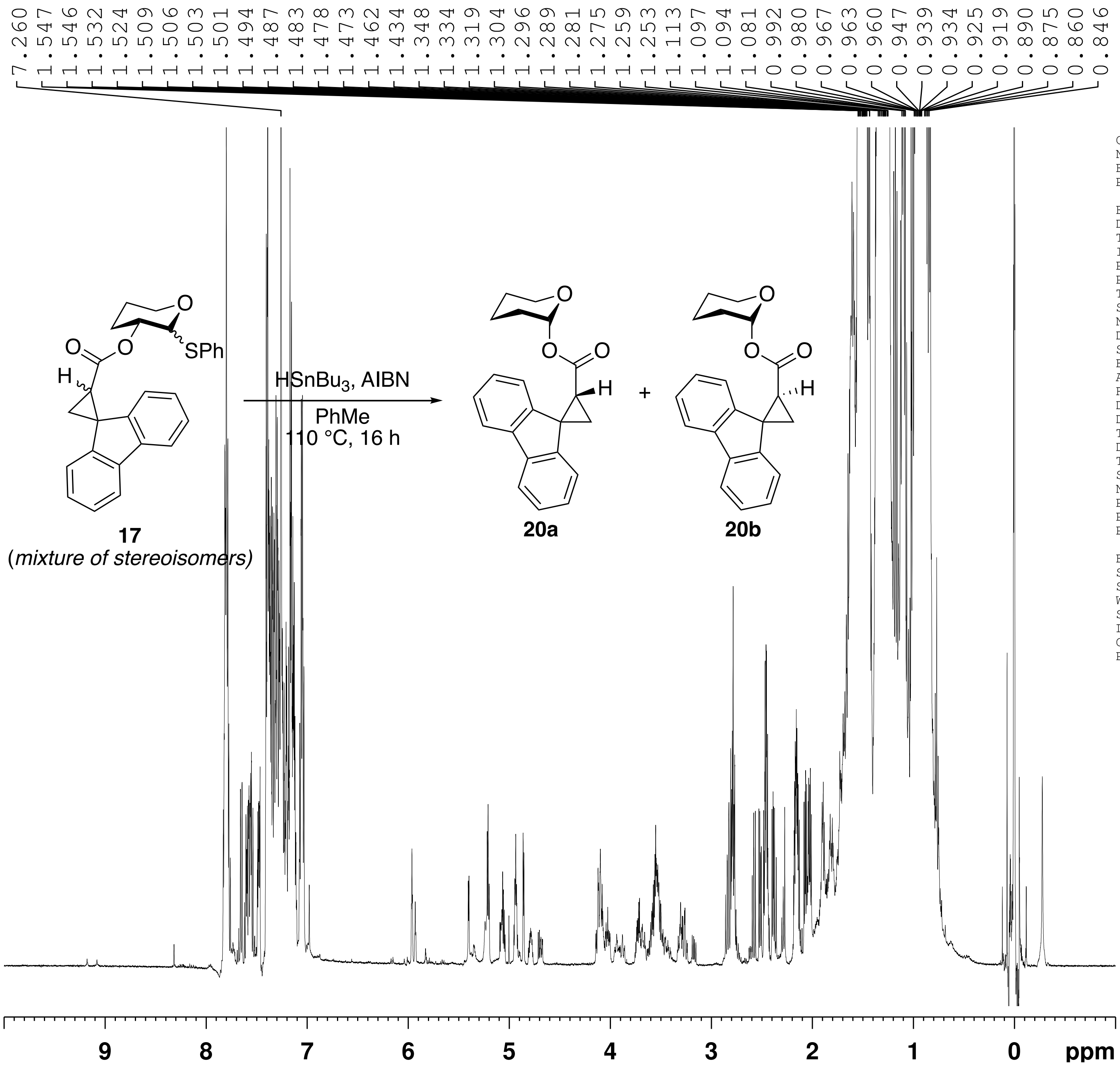


```
Current Data Parameters
NAME     CHW-VIII-6_crudePROTON_500
EXPNO                 10
PROCNO                 1

F2 - Acquisition Parameters
Date_           20230214
Time               10.16 h
INSTRUM  Avance Neo 500
PROBHD   Z167894_0001 (
PULPROG            zg30
TD                65536
SOLVENT           CDCl3
NS                   16
DS                    2
SWH           10000.000 Hz
FIDRES         0.305176 Hz
AQ            3.2767999 sec
RG                   32
DW               50.000 usec
DE                29.20 usec
TE                298.0 K
D1           1.00000000 sec
TD0                   1
SFO1        500.2030887 MHz
NUC1                 1H
P0                 4.00 usec
P1                12.00 usec
PLW1        12.03499985 W

F2 - Processing parameters
SI                65536
SF          500.2000122 MHz
WDW                  EM
SSB                   0
LB                 0.30 Hz
GB                    0
PC                 1.00
```
